# Metagenomic Analysis of the Pygmy Loris Fecal Microbiome Reveals Unique Functional Capacity Related to Metabolism of Aromatic Compounds

**DOI:** 10.1371/journal.pone.0056565

**Published:** 2013-02-15

**Authors:** Bo Xu, Weijiang Xu, Fuya Yang, Junjun Li, Yunjuan Yang, Xianghua Tang, Yuelin Mu, Junpei Zhou, Zunxi Huang

**Affiliations:** 1 School of Life Science, Yunnan Normal University, Kunming, China; 2 Engineering Research Center of Sustainable Development and Utilization of Biomass Energy, Ministry of Education, Kunming, China; 3 Key Laboratory of Yunnan for Biomass Energy and Biotechnology of Environment, Kunming, China; 4 Key Laboratory of Enzyme Engineering, Yunnan Normal University, Kunming, China; Teagasc Food Research Centre, Ireland

## Abstract

The animal gastrointestinal tract contains a complex community of microbes, whose composition ultimately reflects the co-evolution of microorganisms with their animal host. An analysis of 78,619 pyrosequencing reads generated from pygmy loris fecal DNA extracts was performed to help better understand the microbial diversity and functional capacity of the pygmy loris gut microbiome. The taxonomic analysis of the metagenomic reads indicated that pygmy loris fecal microbiomes were dominated by Bacteroidetes and Proteobacteria phyla. The hierarchical clustering of several gastrointestinal metagenomes demonstrated the similarities of the microbial community structures of pygmy loris and mouse gut systems despite their differences in functional capacity. The comparative analysis of function classification revealed that the metagenome of the pygmy loris was characterized by an overrepresentation of those sequences involved in aromatic compound metabolism compared with humans and other animals. The key enzymes related to the benzoate degradation pathway were identified based on the Kyoto Encyclopedia of Genes and Genomes pathway assignment. These results would contribute to the limited body of primate metagenome studies and provide a framework for comparative metagenomic analysis between human and non-human primates, as well as a comparative understanding of the evolution of humans and their microbiome. However, future studies on the metagenome sequencing of pygmy loris and other prosimians regarding the effects of age, genetics, and environment on the composition and activity of the metagenomes are required.

## Introduction

The gastrointestinal tract of animals harbors a complex microbial community, and the composition of this community ultimately reflects the co-evolution of microorganisms with their animal host and the diet adopted by the host [1]. As a result of the issues related to health and disease, the structure and function of the gut microbial community of humans has received significant attention from researchers.

Previous studies have proven that the microbiomes of non-human primates (NHPs) exhibit a much higher similarity with those of primates than with other animals [1]. Therefore, the study of the microbiota from these NHPs provides important insights into the reflection of their features in humans. However, only a few reported culture-independent studies on fecal microbiota of non-human primates [2]–[9] are available, leading to limited comparative data on the intestinal microbiota of primates, either in captivity or in the wild. More extensive surveys of primate gastrointestinal microbiomes, particularly prosimian primates, about which little research work has been done [7], combined with comparative analyses of their microbiomes with those of humans are necessary to better understand the evolution of humans and their microbiome.

The pygmy loris (*Nycticebus pygmaeus*) is a small rare nocturnal prosimian primate found mainly in Vietnam, Laos, and China. Being nocturnal, the prosimians are less known than other primates, but are nonetheless important. Given that previous culture-independent 16S rRNA gene-based analyses have revealed impressive microbial diversity in the pygmy loris feces [7], these analyses offer limited information on the physiological role of microbial consortia within a given gut environment. Random sequencing of the metagenomes has allowed scientists to reveal significant differences in metabolic potential within different environments [10].

Recently, next-generation sequencing technologies have been used to characterize the microbial diversity and functional capacity of a range of microbial communities in the gastrointestinal tracts of humans [11]–[14] as well as in several animal species [15]–[18]. The most important advantages of this cloning-independent approach are the avoidance of cloning bias and the bias introduced by PCR amplification. To best of our knowledge, this study was the first to apply a random sample pyrosequencing approach to analyze the metagenome of the pygmy loris to better understand how microbiomes relate to NHPs ecological and evolutionary diversity.

## Materials and Methods

### Fecal Sample Collection

Fresh fecal samples from two pygmy loris were collected from the Daweishan Nature Reserve of Pingbian, Yunnan Province, China, with the permission of the authorities of the Daweishan Nature Reserve of Pingbian. We tracked the two pygmy loris until they defecated; the fecal samples were immediately collected aseptically. The fresh fecal samples were transported to the laboratory on dry ice within 24 hours of collection, and then stored at −80°C until DNA extraction. We brought no toxic substance that would have adverse effects on the biotic community to minimize disturbance in the animal habitats. The research complied with the protocols established by the China Wildlife Conservation Association and adhered to the American Society of Primatologists (ASP) Principles for the Ethical Treatment of Non-Human Primates as well as the legal requirements of China.

### DNA Extraction and Shotgun Pyrosequencing

Genomic DNA were extracted from the fecal samples with the QIAamp DNA stool mini kit (Qiagen, Valencia, CA, USA) following the protocol provided by the supplier (0.25 g of each fecal sample). The quality and quantity of the DNA were determined with a nanodrop (ND-1000) spectrophotometer (Nanodrop Technologies, Wilmington, DE, USA) through agarose gel electrophoresis. The DNA samples from the two pygmy loris were pooled on an equimolar basis. DNA samples were stored frozen (−20°C) until use.

A total of 500 ng of pooled DNA was subjected to library preparation and shotgun pyrosequencing using the Roche 454 GS FLX Titanium System (Roche, Basel, Switzerland). The obtained reads were uploaded to a Metagenome Rapid Annotation Using Subsystem Technology (MG-RAST) [19] under the name WFH_Metagenome and were assigned the Metagenome ID: 4476304.3. The MG-RAST v.3.0 online server quality control pipeline was utilized to remove reads of short length and poor quality before annotation and the analysis of metagenomic data [19]. The pipeline parameters were kept at default settings. The raw sequencing reads were submitted to the Joint Genome Institute's IMG/M-ER annotation pipeline [20].

### Bioinformatics and Statistical Analysis

Comparative metagenomic analysis was performed with both the MG-RAST and IMG/M pipelines. The metagenomic runs from the pygmy loris data were compared with the current publicly available gut metagenomes in each of the two databases. In the MG-RAST metagenomic annotation pipeline, the pygmy loris fecal metagenomic datasets were compared with nine public sets of data from animals, including chicken cecum A (CCA 4440283), chicken cecum B (CCB 4440284), two dog metagenome data sets (K9C 4444164 and K9BP 4444165), lean mouse cecum (LMC 4440463), obese mouse cecum (OMC 4440464), cow rumen (CRP 4441682), human stool metagenome (HSM 4444130), and human F1-S feces metagenome (F1S 4440939). The organisms in MG-RAST were classified through the M5NR protein database (http://tools.metagenomics.anl.gov/m5nr/). The functional annotation and classification relied on the SEED subsystem ([21]; http://www.theseed.org/wiki/Home_of_the_SEED) databases. The maximum e-value of 1e-5, minimum percent identity of 60, and minimum alignment length of 30 were applied as the parameter settings in the analysis. The taxonomic and functional profiles were normalized to determine the differences in the sequencing coverage by calculating the percent distribution prior to downstream statistical analysis. Clustering was performed using Ward's minimum variance with unscaled Manhattan distances [22]. Heat maps were drawn by hierarchal clustering performed with NCSS 2007 (Kaysville, Utah).

Within the IMG/M pipeline, the pygmy loris metagenomic runs were compared with three lean mouse (*Mus musculus* strain C57BL/6J) cecal metagenomes (metagenome names: Mouse Gut Community lean1–3), two obese mouse (*Mus musculus* strain C57BL/6J) cecal metagenomes (metagenome names: Mouse Gut Community ob1–2), and two healthy human fecal metagenomes (metagenome names: Human Gut Community Subject 7–8). Descriptive information about these mouse and human metagenomes can be found in the GOLD database, under the Gm00071 and Gm00052 GOLD IDs, respectively. Within the IMG/M pipeline, the “Compare Genomes” tool was utilized for hierarchical clustering based on the COG protein profiles.

Annotations based on the carbohydrate-active enzymes database ([23]; http://www.cazy.org) were provided for all the reads that passed the MG-RAST QC filter at an E value restriction of 1×10^−6^.

### KEGG Pathway Assignment

Pathway assignments were established using the Kyoto Encyclopedia of Genes and Genomes (KEGG) mapping method [24]. Enzyme commission (EC) numbers were assigned to unique sequences with BLASTX scores in the default parameters determined by searching the protein databases. The sequences were mapped into the KEGG metabolic pathways according to the EC distribution in the pathway database.

## Results and Discussion

The analysis of the reads yielded a high percentage of species identification in complex metagenomes and even higher in less complex samples. Long sequence reads from 454 GS FLX Titanium pyrosequencing provided the high specificity needed to compare the sequenced reads with the DNA or protein databases and allowed the unambiguous assignment of closely related species. The initial pyrosequencing runs yielded 78,619 reads containing 34,473,384 bases of sequence, with an average read length of 438 bp. Prior to further processing, the raw read data were subjected to the MG-RAST v.3.0 online server quality control pipeline [19] to remove duplicate and low quality reads (Table S1). The filtering step removed 22.1% of reads in the sample. The unique sequence reads that passed the QC filtering step were then subjected to further analysis focusing on biodiversity and functional annotation. All reads were deposited in the National Center for Biotechnology Information (NCBI) and can be accessed in the Short Read Archive (SRA) under the accession number SRX160437.

### Phylogenetic Analysis of Pygmy Loris Fecal Bacteria, Eukaryota, Archaea, and Viruses

**Figure 1:**
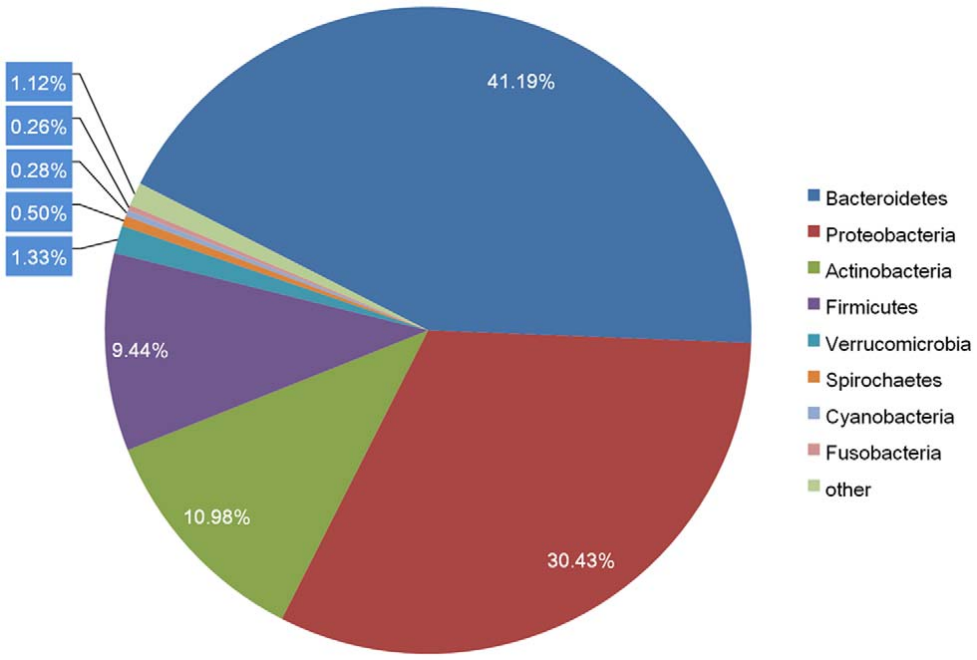
Bacterial phylum profiles of the pygmy loris microbiome. The percentage of the pygmy loris fecal metagenomic sequences assigned to M5NR database is shown. Through the “Organism Abundance” tool in MG-RAST, the pygmy loris fecal sequencing runs were determined from the M5NR database with the BLASTx algorithm. The e-value cutoff for the metagenomic sequence matches to the M5NR database was 1×10^−5^, with a minimum alignment length of 30 bp.

The overview of the phylogenetic computations provided 95.54% bacteria, 3.8% eukaryota, 0.39% archaea, and 0.12% viruses. In the pygmy loris intestinal metagenome, Bacteroidetes was the most predominant phylum (∼41%), followed by Proteobacteria (∼30%), Actinobacteria (∼11%), and Firmicutes (∼9%) (Figure 1). Compared with the previous 16S rRNA gene-based data [7], significantly lower percentages of Firmicutes and higher percentages of Bacteroidetes in the pygmy loris intestinal metagenome were observed. This discrepancy may have been caused by the biases associated with the primers, PCR reaction conditions, or selection of clones [25].

The relative paucity of the Firmicutes sequences is in conflict with data from the studies of humans [1], [11], [12], [26] and other higher primates [2], [3], [4], [6], [8], [9]. The reasons for the variation are difficult to identify because of the biases involved in the fecal lysis and DNA extraction methods [27]; inter-individual variability may also contribute to this divergence. Given that 70% of the phylotypes existing in the human gastro-intestinal microbiome are subject-specific and no phylotype is present at more than 0.5% abundance in all subjects [13], the gastro-intestinal microbiota of each individual has been shown to consist of a subject specific complement of hundreds of genera and thousands of species. As a prosimian, the pygmy loris is less like a primate than others with the same intestinal microbiome composition. However, this claim needs to be proved by further research on fecal samples of more pygmy loris and prosimians.

Within the Bacteroidetes group, Bacteroidales were the most predominant, among which *Bacteroides*, *Prevotella*, and *Parabacteroides* were consistently overrepresented (Table S2). Organisms belonging to the genus *Bacteroides* represent one of the most abundant microbial taxa in human intestinal microbiota [11], [28]. *Bacteroides fragilis* comprises about 5.8% of the reads analyzed; therefore, it is considered the predominant species in the pygmy loris metagenome. *B. fragilis* is a ubiquitous Gram-negative anaerobic bacterium that inhabits the lower GI tract of most mammals [1]. Recent findings have revealed that this organism possesses the ability to direct the cellular and physical maturation of the host immune system and protect its host from experimental colitis [29]–[31]. Natural habitats of *Prevotella* sp. include the rumen and hindgut of cattle, sheep, and humans, where they help break down protein [32] and carbohydrate [33]. However, some species of this genus are known to be opportunistic pathogens to humans [34].

Proteobacteria were the second predominant phylum in the pygmy loris gastrointestinal tract with Pseudomonadales as the primary contributor to the Proteobacteria populations, followed by Enterobacteriales and Burkholderiales. The major genus in the Proteobacteria phylum is *Pseudomonas*, which is consistent with previous 16S rRNA gene-based data [7]. Several microbes belonging to the genus of *Pseudomonas* have a very diverse metabolism, including the ability to degrade organic solvents such as toluene [35] and phenol [36], [37]. This ability may benefit the pygmy loris, given that they consume several toxic and pungent insects. *Pseudomonas fluorescens* was the predominant species among the *Pseudomonas* in the pygmy loris metagenome. *P. fluorescens* is a common Gram-negative bacterium that can be found in the low section of the human digestive tract [38].

Similarly, Clostridia and Bacilli are the primary contributors to the Firmicutes populations. However, various genera were found in the pygmy loris metagenome than in the 16S rRNA gene [7] (Table S2). Clostridiales is the dominant order in Clostridia, which includes well-known gut bacteria, *Faecalibacterium prausnitzii*. *F. prausnitzii* is the most important n-butyrate producing gut bacterium with well-known effects on host energy metabolism and mucosal integrity [39].

**Figure 2:**
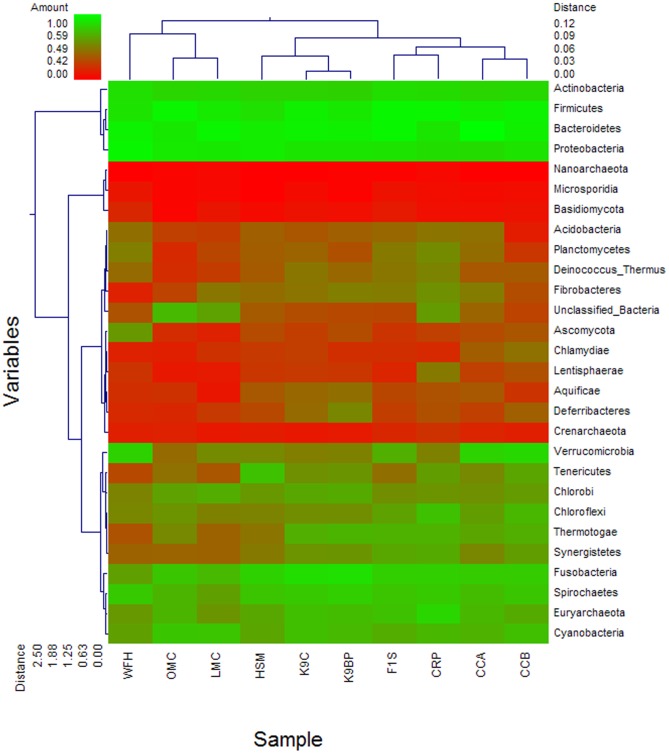
Phylogenetic clustering of pygmy loris, human, mouse, canine, cow, and chicken gastrointestinal metagenomes. A double hierarchical dendrogram was established through weight-pair group clustering methods based on the non-scaling Manhattan distance. The dendrogram shows the phylogenetic distribution of the microorganisms among the ten metagenomes from the six different hosts, including pygmy loris (WFH), human (HSM and F1S), mouse (LMC and OMC), dog (K9C and K9BP), cow (CRP), and chicken (CCA and CCB). The linkages of the dendrogram do not show the phylogenetic relationship of the bacterial phylum and are based on the relative abundance of taxonomic profiles. The heat map depicts the relative percentage of each phylum of microorganism (variables clustering on the y axis) in each sample (x axis clustering). The heat map color represents the relative percentage of the microbial descriptions in each sample, with the legend indicated at the upper left corner. Branch length indicates the Manhattan distances of the samples along the x axis (scale at the upper right corner) and of the microbial phyla along the y axis (scale at the lower left corner).

A distinctive feature of the pygmy loris metagenome is the abundance of phylum Verrucomicrobia, particularly the members of the genus *Akkermansia*; this abundance was unexpected and far greater than in humans (Table S2, Figure 2). The dominant species in the Verrucomicrobia phylum was *Akkermansia muciniphila*, which are common members of the human gut microbiota evident in human infants [40]. These mucin-degrading bacteria are related to normal mucosa development. Moreover, the *Akkermansia* species may have a role in maintaining intestinal integrity.

Eukaryota were a minor constituent (∼4.0%) in the pygmy loris metagenome. Species of *Blastocystis* were also represented in small quantities (<0.01%) in the pygmy loris metagenome. These species have been reported as the most commonly occurring micro-eukaryote in human feces [41], [42]. In addition, the presence of *Blastocystis* has been linked to a number of gut-related diseases. Some of these diseases could be the outcome of the predation of beneficial bacteria by *Blastocystis* in light of the similar observations in ruminant cattle and their communalistic protozoa [43].

Fungi have very low abundance sequences (0.5%), with Eukaryota with Dikarya being the primary contributor, followed by Microsporidia (Table S3). Fungi in the intestinal ecosystem of NHPs have not yet been studied extensively. Through culture-independent methods, Scanlan and Marchesi revealed that more diverse fungi species are found in the human distal gut, including *Saccharomyces*, *Gloeotinia*, *Penicillium*, *Candida*, and *Galactomyces* [41]. Similar phylotypes of fungi (*Candida*, *Cladosporium*, *Penicillium*, and *Saccharomyces*) have also been identified in stool samples from patients with human inflammatory bowel disease and from healthy control subjects [44]. Compared with humans, more diverse fungi species belonging to 29 different genera exist in the pygmy loris metagenome (Table S3). The most abundant fungi genera in the pygmy loris metagenome were *Aspergillus* (0.04%), *Neurospora* (0.03%), *Gibberella* (0.03%), and *Neosartorya* (0.03%). Three fungi species (*Gibberella zeae*, *Neurospora crassa*, and *Saccharomyces cerevisiae*) have been identified in the pygmy loris metagenome, which were also identified in feline [18], canine (K9C and K9BP) [16], and mouse metagenomes (OMC) [45].

*Enterocytozoon bieneusi* is the only species found in Microsporidia. Microsporidia are enteric protozoan parasites recognized as important pathogens in immunocompromised persons and malnourished children. *E. bieneusi* is the most common microsporidian species in humans, infecting enterocytes and other epithelial cells [46]. Interestingly, our study demonstrated a low percentage of fungi with high diversity among the fecal microflora. Future studies on the fungal diversity in the pygmy loris gastrointestinal tract would benefit from this next-generation sequencing.

Archaea are a minor component of the pygmy loris metagenome, comprising ∼0.4% of the total sequencing reads. Archaea consist of two phyla, Euryarchaeota and Crenarchaeota, which diverged into seven classes and eight orders (Table S4). Among the groups of archaea, methanogenic archaea is the most predominant and diverse group. Methanogenic archaea is also widespread in a variety of vertebrates, such as felines, dogs, humans, mice, and chicken [15], [16], [18], [45], [47]. In the pygmy loris fecal metagenome, *Methanocorpusculum labreanum* is the major component of archaea, having a percentage of 0.03% in all the analytic sequences (Table S4). Although archaea have been observed in NHPs previously [5], the diversity of this domain has not been elucidated yet.

Archaea are considered commensals; however, they contribute to pathogeny in humans because of mutual interactions with other microorganisms [48]. For instance, methanogens consume hydrogen and create an environment that enhances the growth of polysaccharide fermenting bacteria, leading to higher energy utilization. Higher numbers of methanogenic archaea have been observed in obese humans [49]. However, the prevalence and medical importance of archaea in NHPs need to be determined. Despite the ability to carry methanogens in the intestinal tracts of animals linked to phylogeny [50], the gut archaea in the pygmy loris metagenome are not similar to human ones than to those of other species.

Only ∼0.1% of the total reads have viral origin, with only the order Caudovirales being identified. Two families were observed (Myoviridae and Siphoviridae) within the Caudovirales order, and all sequences were classified as bacteriophages (Table S5). Bacteriophages influence food digestion by regulating microbial communities in the human GI tract through lytic and lysogenic replication [51]. Bacteriophages also contribute to human health by controlling invading pathogens [52]. Recent metagenomic analyses of the DNA viruses from human feces have revealed that the majority of DNA viruses in human feces are novel, and most of the recognizable sequences also belong to bacteriophages [53]. The close phylogenetic relationship between humans and NHPs, coupled with the exponential expansion of human populations and human activities within the primate habitats, has resulted in the exceptionally high possibility of pathogen exchange [54]. Therefore, studies on the viral community of NHPs and the potential for cross-transmission between humans and NHPs are needed. Given the type of methodology (shotgun DNA pyrosequencing approach) that we utilized, our study could only determine the dsDNA virus. Future studies need to provide a richer understanding of both RNA and dsDNA viruses to complete human knowledge of the viral intestinal ecosystem.

Studies on cow [55], cats [56], mice [57], rats [58] as well as humans [59] and NHPs [5] revealed a correlation between host diet and microbial community composition. However, the characterization of the dietary-induced changes in NHP microbiomes through high-throughput sequencing technologies has not been performed thus far. Hence, more attention should be given to it in future experiments.

### Metabolic Profiles of the Pygmy Loris Metagenome

**Figure 3:**
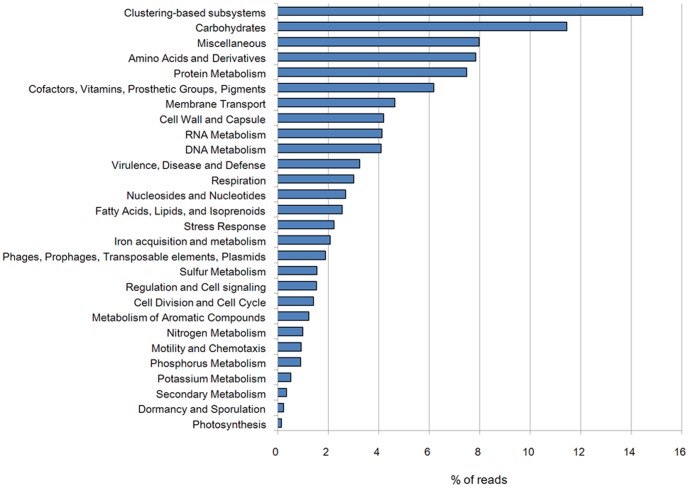
Functional composition of the pygmy loris microbiome. The percentage of the pygmy loris fecal metagenomic sequences assigned to the general SEED subsystems is shown. Through the “Functional Abundance” tool in MG-RAST, the pygmy loris fecal sequencing runs were determined from the SEED database with the BLASTx algorithm. The e-value cutoff for the metagenomic sequence matches to the SEED subsystem database was 1×10^−5^ with a minimum alignment length of 30 bp.

Carbohydrate metabolism is the most abundant functional category, representing 11.45% of the pygmy loris fecal metagenomes (Figure 3). Genes associated with amino acids and derivatives, protein metabolism, cofactors (vitamins, prosthetic groups, pigments), membrane transport, cell wall and capsule. RNA metabolism and DNA metabolism are also very abundant in the pygmy loris metagenomes. Approximately 14.44% of the annotated reads from the pygmy loris fecal metagenomes were categorized within the clustering-based subsystems, most of which have unknown or putative functions.

We subjected our samples to the carbohydrate-active enzymes database (CAZy; http://www.cazy.org), as described by Cantarel et al. [23], to obtain a more in-depth view of the carbohydrate enzymes present in our data set. The comparison of the 61,281 metagenome reads post-QC processing based on the CAZy database provided 2260 hits at an E value restriction of 1×10^−6^. Candidate sequences that belong to the glycosyl transferase families GT2 (148) are the most abundant, followed by members of the glycoside hydrolase (GH) families GH3 (142) and GH2 (134) (Figure S1 and Table S6).

GHs are a prominent group of enzymes that hydrolyze the glycosidic bond among the carbohydrate molecules. The most frequently occurring GH families in the pygmy loris metagenome were GH3 (142; 8.96% of the total GH matches), 2 (134; 8.45%), and 43 (103; 6.5%) (Figure S1 and Table S6). The most common activities of GH3 include β-D-glucosidases, α-L-arabinofuranosidases, β-D-xylopyranosidases, and N-acetyl-β-D-glucosaminidases [60]. In several cases, the enzymes have dual or broad substrate specificities with respect to monosaccharide residue, linkage position, and chain length of the substrate, such as α-L-arabinofuranosidase and β-D-xylopyranosidase [61]. GH2 components are β-D-galactosidases, β-glucuronidases, β-D-mannosidases, and exo-β-glucosaminidases. GH43 shows β-xylosidase, β-1,3-xylosidase, α-L-arabinofuranosidase, arabinanase, xylanase, and galactan 1,3-β-galactosidase activity (www.cazy.org).

Glycosyl transferases are ubiquitous enzymes that catalyze the attachment of sugars to a glycone [62]. Candidate genes that belong to the glycosyl transferase families GT2 (148; 35.24% of the total GT matches) and GT4 (96; 22.86%) are the most abundant (Figure S1).

### Comparative Metagenomic Analysis

Despite the extensive variation among individuals, the gut microbiota of members of the same species are often more similar to one another compared with those of other species. Both humans and the pygmy loris are primates; however, the latter are prosimians and are different from humans in terms of primate evolution. The human gut is a natural habitat for various communities of microorganisms that have co-evolved with humans. Thus, it is important to provide a comparison between the gastrointestinal microbiomes of primates and those of other animals.

The results of this study were compared with data sets from different animals and even humans in the MG-RAST database. Paired data from other studies were chosen, such as lean (LMC) and obese (OMC) mouse cecal metagenomes [45], two chicken cecal metagenomes (CCA, CCB) [15], two canine intestinal metagenomes (K9C, K9BP) [16], and two human fecal metagenomes (F1S, HSM). F1S was a healthy human fecal metagenome [47], whereas HSM was defined as human feces from a malnourished subject. A single cow rumen metagenome (4441682.3) was also utilized for comparison. The comprehensive overview of the ten data sets is shown in Table S7.

**Figure 4:**
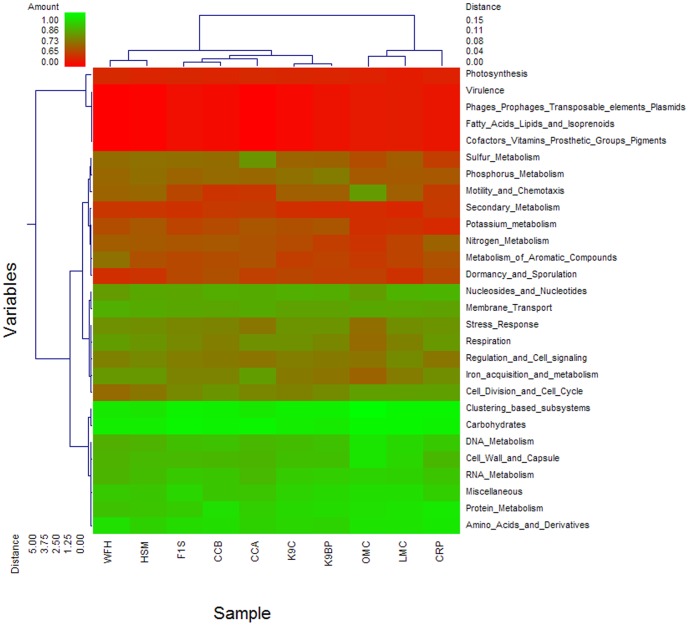
Metabolic clustering of pygmy loris, human, mouse, canine, cow, and chicken gastrointestinal metagenomes. A double hierarchical dendrogram was established through a weight-pair group clustering method based on the non-scaling Manhattan distance. The dendrogram shows the distribution of the functional categories among the ten metagenomes from the six different hosts, including pygmy loris (WFH), humans (HSM and F1S), murine (LMC and OMC), canine (K9C and K9BP), cow (CRP), and chicken (CCA and CCB). The linkages of the dendrogram are based on the relative abundance of metabolic profiles. The heat map depicts the relative percentage of each category of function (variables clustering on the y axis) in each sample (x axis clustering). The heat map color represents the relative percentage of functional categories in each sample, with the legend indicated at the upper left corner. Branch length indicates the Manhattan distances of the samples along the x axis (scale at the upper right corner) and of the microbial classes along the y axis (scale at the lower left corner).

Clustering the metagenomes was carried out with unscaled Manhattan variance distances and presented through a double hierarchical dendrogram. The clustering-based comparisons were demonstrated at the phylogenetic level (Figure 2) and the metabolic level (Figure 4). In the phylogenetic comparison, the pygmy loris samples clustered with the two mouse metagenomes (OMC and LMC) and separated from those of the other animals and humans. The results do not correspond with previous studies, indicating that microbial community composition is more similar among primates than in other animals [1]. Although pygmy loris belong to primates,they are prosimians, or pre-monkeys and are different from humans in terms of primate evolution. Therefore, the gut microbiomes of the pygmy loris may show an obvious difference compared with human gastrointestinal microbiomes. Meanwhile, those of mice have a more exact similarity with the human genome; more than 90% of the mouse genome is similar to the human genome [63]. The microorganism composition of the animal gastrointestinal tract reflects the constant co-evolution of the animal with its host [28]. The clustering of the pygmy loris metagenome with that of the mouse metagenome may be a result of similar bacterial diversity influenced by co-evolution with the host. Similar clustering of mouse and human data from the IMG/M ER database was performed. Figure S2 demonstrates that the pygmy loris samples clustered with the mouse gut samples.

In all the samples, the Bacteroidetes, Firmicutes, Actinobacteria, and Proteobacteria were the most abundant. The pygmy loris metagenome was most distinguished by the greater prevalence of Verrucomicrobia compared with mice and other animals, as shown in Figure 2. The heat map also demonstrates that the pygmy loris fecal metagenome contains lower Fibrobacteres, an important phylum of cellulose-degrading bacteria.

**Figure 5:**
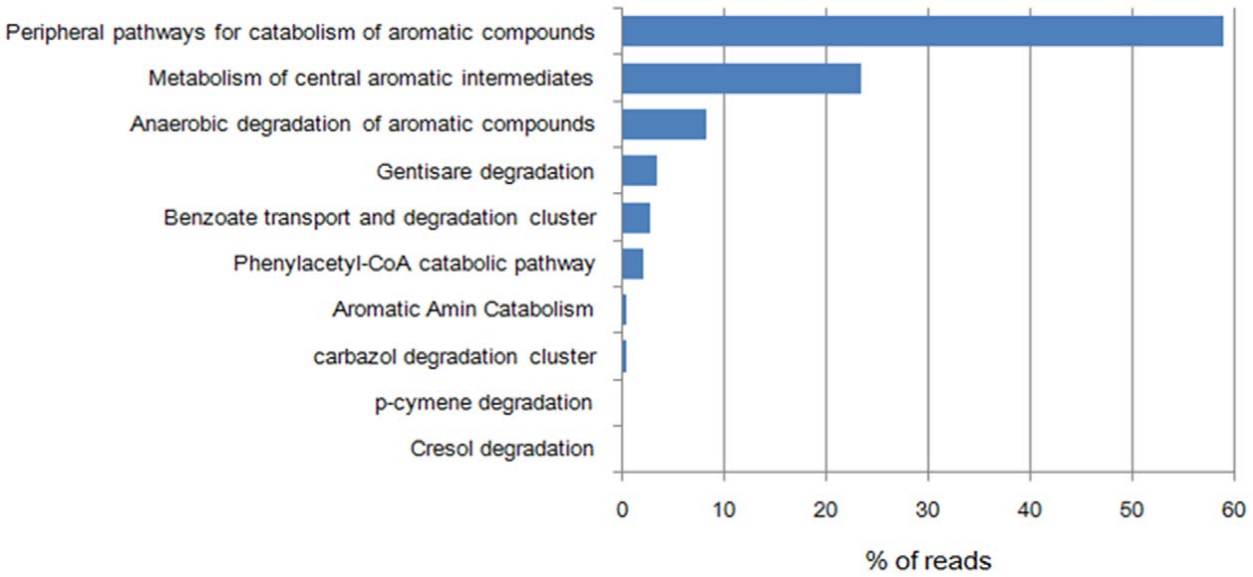
Percentage of sequences associated with the metabolism of aromatic compounds in the pygmy loris microbiome. Through the “Metabolic Analysis” tool in MG-RAST, the pygmy loris fecal sequencing runs were determined from the SEED database with the BLASTx algorithm. The e-value cutoff for the metagenomic sequence matches to the SEED subsystem database was 1×10^−5^ with a minimum alignment length of 30 bp.

Metabolism-based hierarchical clustering demonstrates that the pygmy loris, human, chicken, and dog samples clustered together. The mice and cow samples were the least similar samples to the pygmy loris (Figure 4). The similarity of function among pygmy loris, humans, chicken, and dogs is not surprising, considering the fact that they are all omnivores with similar digestive tract structures and functions. Similar clustering of mouse and human data from the IMG/M ER database was performed. Figure S3 demonstrates that the pygmy loris samples clustered with the human gut samples. Interestingly, the two mouse samples that clustered together were most similar to the cow rumen samples, that is, those of an herbivore. As expected, all the gut metagenomes were dominated by carbohydrate metabolism subsystems with amino acids, protein, and cell wall and capsule; the DNA and RNA subsystems were represented in relatively high abundance as well. An interesting result was observed in terms of the metabolism of aromatic compounds, which accounted for a higher number of reads in the pygmy loris fecal metagenome than in other animals (Figure 4). The profile of the metabolism of aromatic compounds in the pygmy loris fecal metagenomes was dominated by proteins annotated as subsystems of peripheral pathways for catabolism of aromatic compounds, metabolism of central aromatic intermediates, and anaerobic degradation of aromatic compounds (Figure 5).

### KEGG Pathway Assignment

Pathway assignment was performed based on the Kyoto Encyclopedia of Genes and Genomes (KEGG). First, the 78,619 reads were compared using BLASTX with the default parameters from the KEGG database. A total of 18,410 reads with corresponding enzyme commission (EC) numbers were assigned to the metabolic pathways. Given that the sequences related to the metabolism of aromatic compounds were more abundant in the pygmy loris fecal metagenomes compared with other animals in terms of subsystem, we focused our attention on xenobiotic biodegradation and metabolism.

**Figure 6:**
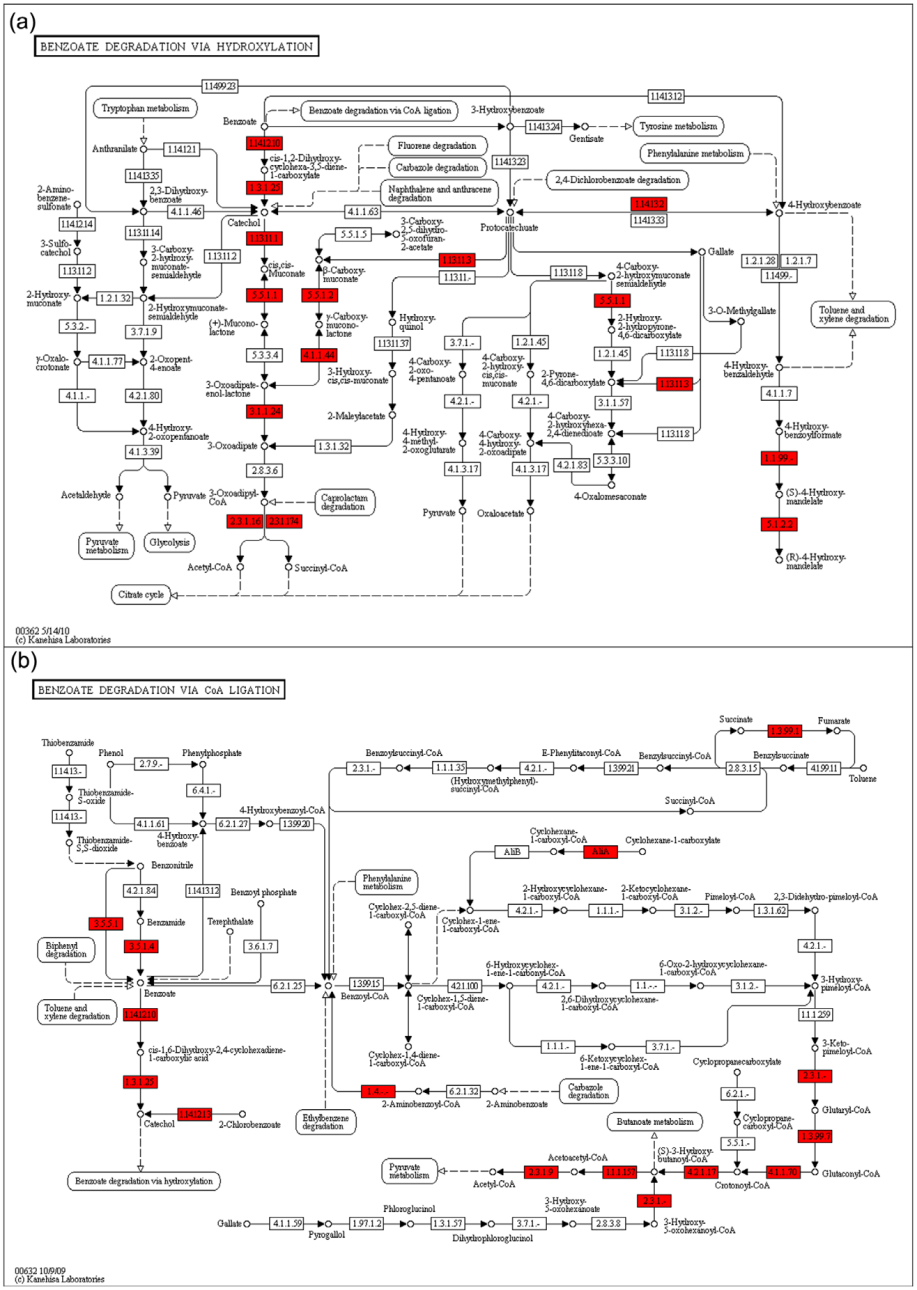
Reference pathway of benzoate degradation. a. Benzoate degradation by the hydroxylation reference pathway. b. Benzoate degradation by the CoA ligation reference pathway. The boxes colored red represent the enzymes identified from the pygmy loris fecal metagenomes based on KEGG.

A high number of sequences in the benzoate degradation pathway was observed, which is coherent with the fact that benzoate is a central intermediary compound in the anaerobic and aerobic metabolism of various aromatic compounds, such as toluence, xylene, fluorine, carbazole, and biphenyl [64]. The key enzymes involved in benzoate degradation via hydroxylation, such as catechol 1,2-dioxygenase (EC 1.13.11.1), and protocatechuate 3,4-dioxygenase (EC 1.13.11.3) were identified in the pygmy loris fecal metagenomes (Figure 6a). The two usual methods of aerobic benzoate metabolism are dioxygenation to form catechol, utilized by some bacteria such as *Pseudomonas putida* and *Acinetobacter calcoaceticus* [65], and monooxygenation to form protocatechuate, mostly by *Aspergillus niger* [66]. Almost all the enzymes involved in the two methods of aerobic benzoate metabolism in the KEGG pathway (Figure 6a). The main organisms, *P. putida*, *A. calcoaceticus*, and *A. niger*, involved in the course of metabolism were all represented in the pygmy loris fecal microbiome (Table S2 and S3).

Although several key enzymes such as benzoyl-CoA reductase (EC 1.3.99.15) were missing in the method of anaerobic benzoate metabolism via CoA ligation, partial enzymes were identified (Figure 6b). This particular result may be due to the fact that the pathway of anaerobic benzoate metabolism in the pygmy loris was a little different. These results suggest that the fecal microbiota of the pygmy loris under study have a potential to degrade phenol and derivatives by the aerobic and anaerobic pathway. Moreover, these pathways may interchange because of the cross-regulation between the anaerobic and aerobic pathways for the catabolism of aromatic compounds, which may reflect a biological strategy to increase cell fitness in organisms that survive in environments subject to changing oxygen concentrations [67].

Aromatic compounds comprise one-quarter of the Earth's biomass and are the second most widely distributed class of organic compounds in nature, next to carbohydrates. Benzoate is produced from the microbial degradation of dietary aromatic compounds in the intestine [68] and is also naturally present in most berries, fruits, and fermented dairy products [69]. Normal patterns of personal dietary intake alters the phenolic substrates supplied to the intestinal bacteria and the aromatic metabolites formed, resulting in possible fluctuations in the microflora population [70]. The pygmy loris is an insectivorous species that also eats fruits, birds' eggs, chicks, geckos, and arboreal small mammals [71]. Such diet rich in aromatic compounds could result in the relative abundance of sequence-encoding enzymes and microbiota involved in benzoate degradation. Therefore, more attention should be given to the potential new genes and pathways generated by the metabolism of aromatic compounds in the pygmy loris fecal microbiome.

## Conclusions

We presented for the first-time the application of the shotgun metagenomic pyrosequencing approach to study the fecal microbiome of the pygmy loris. The overall goal of this study was to characterize the species composition and the functional capacity of the pygmy loris fecal microbiome. Taxonomic analysis of the metagenomic reads showed similarities among the gut microbiomes of the pygmy loris, humans, and other animals. Four phyla dominated the microbiomes, namely, Bacteroidetes, Proteobacteria, Actinobacteria, and Firmicutes. However, the relative proportion of the phyla was different; most of the less abundant phyla such as Proteobacteria and Actinobacteria were more prevalent, and most of the more abundant phyla such as Firmicutes were fewer in the pygmy loris fecal microbiome than in humans and other animals. At the genus-level taxonomic resolution, *Bacteroides* species were the most abundant, most of which were represented by *B. fragilis*. The organisms belonging to the said genus also represent one of the most abundant microbial taxa in the human intestinal microbiota [11], [28]. The pygmy loris faecal samples contained more bacteria belonging to the phylum Verrucomicrobia, most of which were represented by the mucin-degrading bacterium *A. muciniphilia*. The high amount of *A. muciniphilia* present in the pygmy loris feces indicates a high turnover of mucins in these prosimians. Archaea, fungi, and viruses are minor constituents of the pygmy loris fecal microbiome. All archaea are members of Crenarchaeota and Euryarchaeota, with methanogens being the most abundant and diverse. Three fungi phylotypes were present in the pygmy loris fecal microbiome, namely, Ascomycota, Basidiomycota, and Microsporidia. Only about 0.1% of sequences were of viral origin, and all sequences were classified as bacteriophages.

The hierarchical clustering of the gut metagenomic data from pygmy loris, humans, dogs, mice, chickens, and cows demonstrated the similarity of the microbial community structures of the pygmy loris and mouse gut systems despite the differences in functional capacity. The comparison of the fecal microbiota of NHPs with the microbiota of humans and other animals obtained in previous studies revealed that the gut microbiota of the pygmy loris are distinct and reflect host phylogeny. The comparative metagenomic analyses identified unique and/or overabundant taxonomic and functional elements in the pygmy loris distal gut microbiomes. Relatively abundant and diverse metabolic subsystems of aromatic compounds were found in the pygmy loris metagenome compared with all the other gut metagenomes.

These results contribute to the limited body of primate metagenome studies and provide a framework for the comparative metagenomic analysis of the human and NHP metagenome, as well as a comparative understanding of the evolution of humans and their microbiome. More studies involving the deeper sequencing of metagenomes are required to fully characterize the gastrointestinal microbiome of the pygmy loris and other prosimians in healthy and diseased states, of varying ages or genetic backgrounds, and in the wild or in captivity.

## Supporting Information

Figure S1 **Number of occurrences for the 25 most dominant carbohydrate active enzyme families.** In total, these families contain 1468 sequences, representing 64.6% of the putative carbohydrate active enzyme family.
(TIF)

Figure S2 **Bacterial clustering of pygmy loris, human, and mouse gastrointestinal metagenomes.** A dendrogram, constructed using IMG/M ER.
(TIF)

Figure S3 **Hierarchical clustering of functional genes from pygmy loris, human, and mouse gastrointestinal metagenomes.** A matrix consisting of the number of reads assigned to COGs was generated using the “Compare Genomes” tool in IMG/M ER.
(TIF)

Table S1 **Information regarding the sequence datas.**
(DOCX)

Table S2 **Phylogenetic classification of bacteria in the pygmy loris metagenome.**
(DOCX)

Table S3 **Phylogenetic classification of fungi in the pygmy loris metagenome.**
(DOCX)

Table S4 **Phylogenetic classification of archaea in the pygmy loris metagenome.**
(DOCX)

Table S5 **Phylogenetic classification of viruses in the pygmy loris metagenome.**
(DOCX)

Table S6 **Presence of carbohydrate active enzyme families in the pygmy loris metagenome.**
(DOCX)

Table S7 **Overview of the MG-RAST metagenomes chosen for comparison.**
(DOCX)
