# Supplementary figures and images for: Metagenomic Analysis of the Pygmy Loris Fecal Microbiome Reveals Unique Functional Capacity Related to Metabolism of Aromatic Compounds
Source: PLoS One. 2013 Feb 15;8(2):e56565. doi: 10.1371/journal.pone.0056565 (PMC3574064; doi:10.1371/journal.pone.0056565)

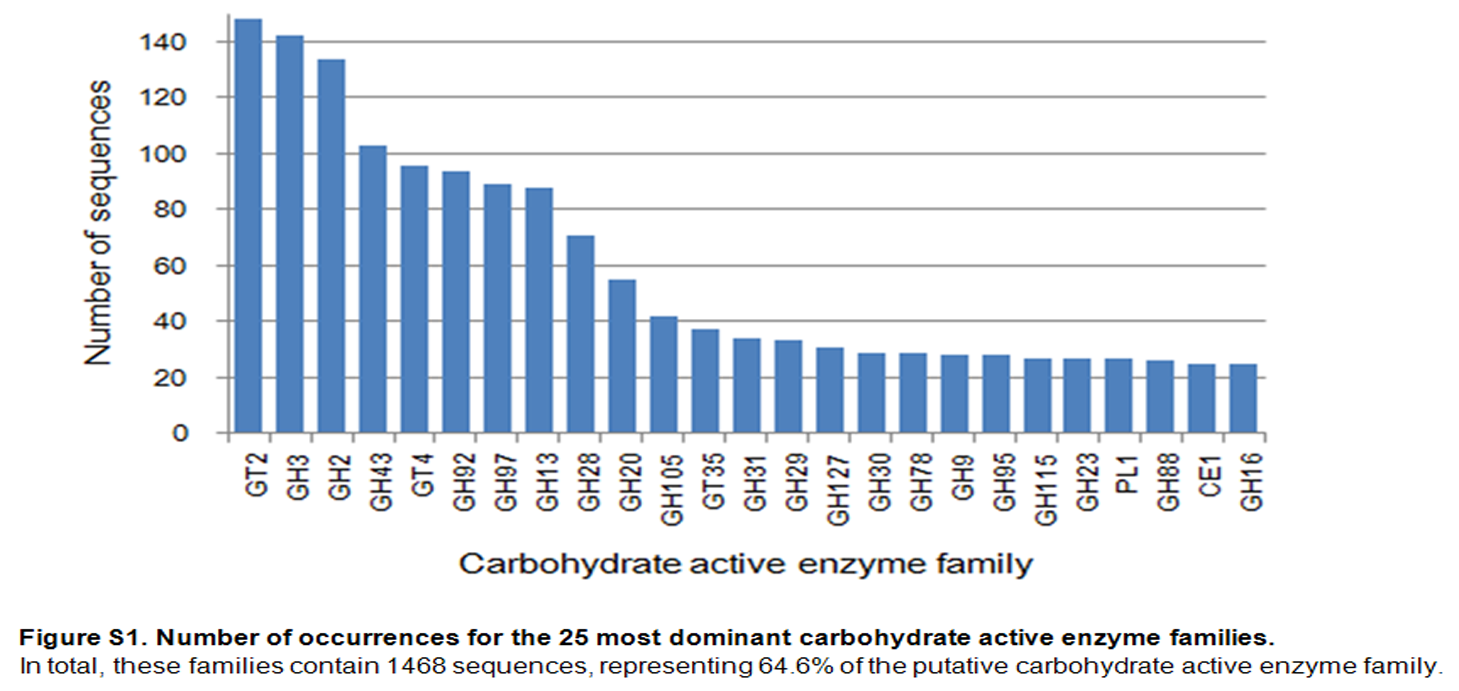

Supplement: Figure S1 — Number of occurrences for the 25 most dominant carbohydrate active enzyme families. In total, these families contain 1468 sequences, representing 64.6% of the putative carbohydrate active enzyme family. (TIF) [file pone.0056565.s001.tif]

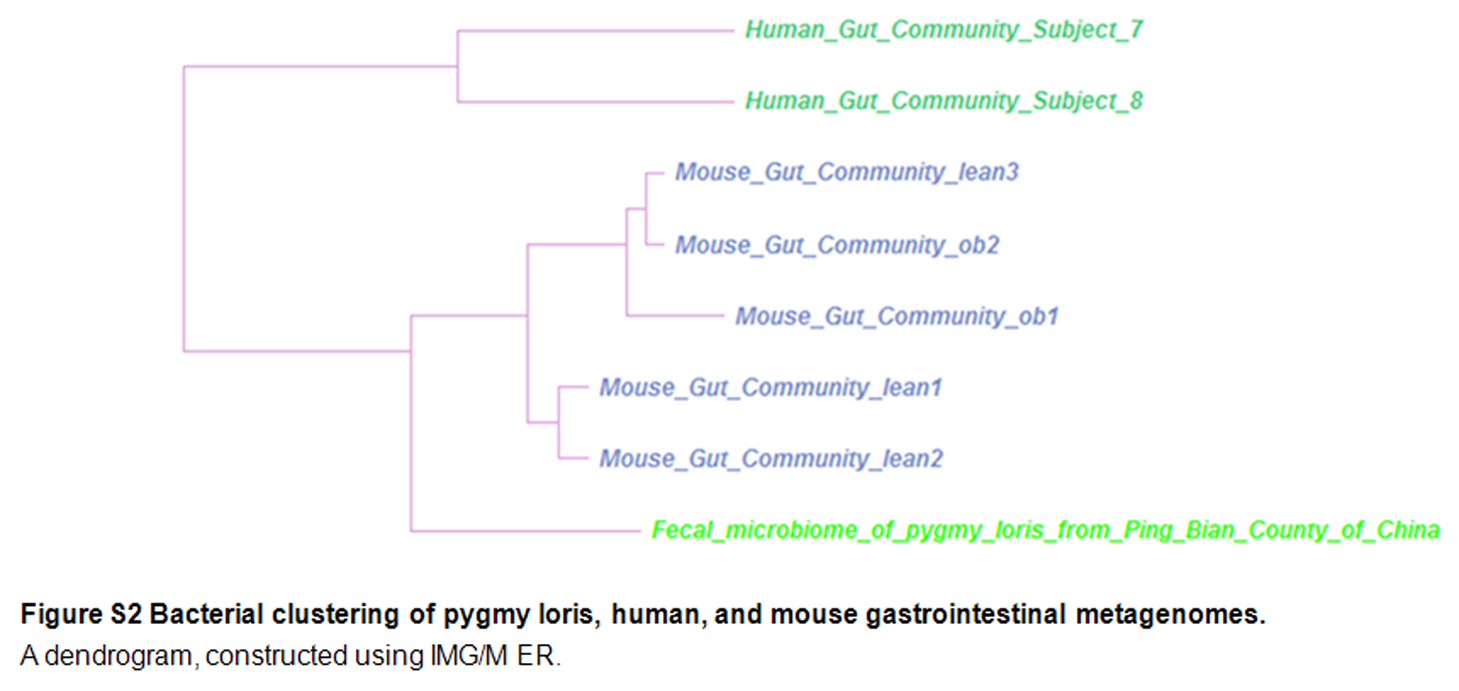

Supplement: Figure S2 — Bacterial clustering of pygmy loris, human, and mouse gastrointestinal metagenomes. A dendrogram, constructed using IMG/M ER. (TIF) [file pone.0056565.s002.tif]

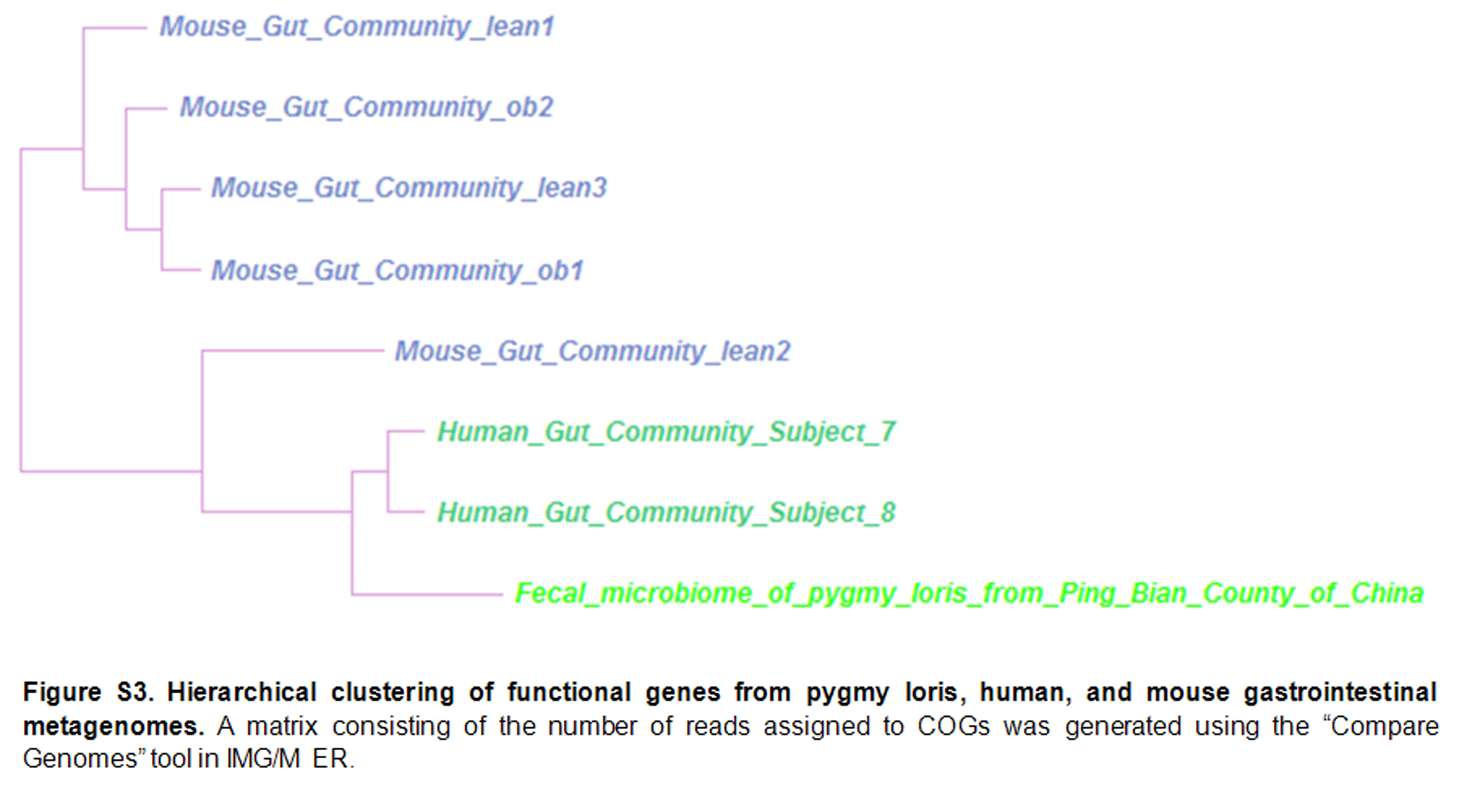

Supplement: Figure S3 — Hierarchical clustering of functional genes from pygmy loris, human, and mouse gastrointestinal metagenomes. A matrix consisting of the number of reads assigned to COGs was generated using the “Compare Genomes” tool in IMG/M ER. (TIF) [file pone.0056565.s003.tif]
