# Supplementary material for: Metagenomic Analysis of the Pygmy Loris Fecal Microbiome Reveals Unique Functional Capacity Related to Metabolism of Aromatic Compounds
Source: PLoS One. 2013 Feb 15;8(2):e56565. doi: 10.1371/journal.pone.0056565 (PMC3574064; doi:10.1371/journal.pone.0056565)
Supplement: Table S1 — Information regarding the sequence datas. (DOCX) [file pone.0056565.s004.docx]

Table S1. Information regarding the sequence datas

|  | Pygmy loris metagenome |
| --- | --- |
| Raw data |  |
| Total # of reads | 78,619 |
| Total bp | 34,473,384 |
| Mean length (bp) | 438 ± 122 |
| Mean GC % | 53 ± 9 % |
| MG-RAST Analysis |  |
| Total # of reads | 78,619 |
| Total bp | 34,473,384 |
| Mean length (bp) | 438 ± 122 |
| Mean GC % | 53 ± 9 % |
| Total # of reads post QC | 61,281 |
| Total bp post QC | 27,187,688 |
| Mean length post QC (bp) | 443 ± 121 |
| Mean GC % post QC | 53 ± 9 % |
| Processed predicted protein features | 61,685 |
| Processed predicted rRNA features | 11,471 |

QC: MG-RAST 3.0 applied quality control of the reads.
