# Supplementary material for: Metagenomic Analysis of the Pygmy Loris Fecal Microbiome Reveals Unique Functional Capacity Related to Metabolism of Aromatic Compounds
Source: PLoS One. 2013 Feb 15;8(2):e56565. doi: 10.1371/journal.pone.0056565 (PMC3574064; doi:10.1371/journal.pone.0056565)
Supplement: Table S2 — Phylogenetic classification of bacteria in the pygmy loris metagenome. (DOCX) [file pone.0056565.s005.docx]

**Table S2. Phylogenetic classification of bacteria in the pygmy loris metagenome**

| phylum | order | genus | species | WFH (%) |
| --- | --- | --- | --- | --- |
| Acidobacteria | Acidobacteriales | Acidobacterium | Acidobacterium capsulatum | 0.03 |
| Acidobacteria | Acidobacteriales | Acidobacterium | Acidobacterium sp. MP5ACTX8 | 0.01 |
| Acidobacteria | Acidobacteriales | Acidobacterium | Acidobacterium sp. MP5ACTX9 | 0.01 |
| Acidobacteria | Acidobacteriales | Terriglobus | Terriglobus saanensis | 0.02 |
| Acidobacteria | Solibacterales | Candidatus Solibacter | Candidatus Solibacter usitatus | 0.08 |
| Acidobacteria | unclassified (derived from Acidobacteria) | Candidatus Koribacter | Candidatus Koribacter versatilis | 0.05 |
| Actinobacteria | Acidimicrobiales | Acidimicrobium | Acidimicrobium ferrooxidans | 0.01 |
| Actinobacteria | Actinomycetales | Acidothermus | Acidothermus cellulolyticus | 0.08 |
| Actinobacteria | Actinomycetales | Actinomyces | Actinomyces coleocanis | 0.02 |
| Actinobacteria | Actinomycetales | Actinomyces | Actinomyces odontolyticus | 0.05 |
| Actinobacteria | Actinomycetales | Actinomyces | Actinomyces sp. | 0.03 |
| Actinobacteria | Actinomycetales | Actinomyces | Actinomyces sp. oral taxon 171 | 0.03 |
| Actinobacteria | Actinomycetales | Actinomyces | Actinomyces sp. oral taxon 178 | 0.02 |
| Actinobacteria | Actinomycetales | Actinomyces | Actinomyces sp. oral taxon 180 | 0.02 |
| Actinobacteria | Actinomycetales | Actinomyces | Actinomyces sp. oral taxon 848 | 0.03 |
| Actinobacteria | Actinomycetales | Actinomyces | Actinomyces urogenitalis | 0.03 |
| Actinobacteria | Actinomycetales | Actinomyces | Actinomyces viscosus | 0.03 |
| Actinobacteria | Actinomycetales | Arcanobacterium | Arcanobacterium haemolyticum | 0.03 |
| Actinobacteria | Actinomycetales | Mobiluncus | Mobiluncus curtisii | 0.03 |
| Actinobacteria | Actinomycetales | Mobiluncus | Mobiluncus mulieris | 0.03 |
| Actinobacteria | Actinomycetales | Actinosynnema | Actinosynnema mirum | 0.11 |
| Actinobacteria | Actinomycetales | Beutenbergia | Beutenbergia cavernae | 0.17 |
| Actinobacteria | Actinomycetales | Brevibacterium | Brevibacterium linens | 0.50 |
| Actinobacteria | Actinomycetales | Brevibacterium | Brevibacterium mcbrellneri | 0.20 |
| Actinobacteria | Actinomycetales | Catenulispora | Catenulispora acidiphila | 0.11 |
| Actinobacteria | Actinomycetales | Cellulomonas | Cellulomonas flavigena | 0.17 |
| Actinobacteria | Actinomycetales | Corynebacterium | Corynebacterium accolens | 0.07 |
| Actinobacteria | Actinomycetales | Corynebacterium | Corynebacterium ammoniagenes | 0.10 |
| Actinobacteria | Actinomycetales | Corynebacterium | Corynebacterium amycolatum | 0.12 |
| Actinobacteria | Actinomycetales | Corynebacterium | Corynebacterium aurimucosum | 0.14 |
| Actinobacteria | Actinomycetales | Corynebacterium | Corynebacterium diphtheriae | 0.20 |
| Actinobacteria | Actinomycetales | Corynebacterium | Corynebacterium efficiens | 0.45 |
| Actinobacteria | Actinomycetales | Corynebacterium | Corynebacterium genitalium | 0.08 |
| Actinobacteria | Actinomycetales | Corynebacterium | Corynebacterium glucuronolyticum | 0.05 |
| Actinobacteria | Actinomycetales | Corynebacterium | Corynebacterium glutamicum | 0.60 |
| Actinobacteria | Actinomycetales | Corynebacterium | Corynebacterium jeikeium | 0.25 |
| Actinobacteria | Actinomycetales | Corynebacterium | Corynebacterium kroppenstedtii | 0.11 |
| Actinobacteria | Actinomycetales | Corynebacterium | Corynebacterium lipophiloflavum | 0.07 |
| Actinobacteria | Actinomycetales | Corynebacterium | Corynebacterium matruchotii | 0.07 |
| Actinobacteria | Actinomycetales | Corynebacterium | Corynebacterium pseudogenitalium | 0.05 |
| Actinobacteria | Actinomycetales | Corynebacterium | Corynebacterium pseudotuberculosis | 0.13 |
| Actinobacteria | Actinomycetales | Corynebacterium | Corynebacterium resistens | 0.03 |
| Actinobacteria | Actinomycetales | Corynebacterium | Corynebacterium striatum | 0.10 |
| Actinobacteria | Actinomycetales | Corynebacterium | Corynebacterium tuberculostearicum | 0.06 |
| Actinobacteria | Actinomycetales | Corynebacterium | Corynebacterium urealyticum | 0.13 |
| Actinobacteria | Actinomycetales | Brachybacterium | Brachybacterium faecium | 0.70 |
| Actinobacteria | Actinomycetales | Dermacoccus | Dermacoccus sp. Ellin185 | 0.03 |
| Actinobacteria | Actinomycetales | Kytococcus | Kytococcus sedentarius | 0.09 |
| Actinobacteria | Actinomycetales | Dietzia | Dietzia cinnamea | 0.14 |
| Actinobacteria | Actinomycetales | Frankia | Frankia alni | 0.09 |
| Actinobacteria | Actinomycetales | Frankia | Frankia sp. | 0.14 |
| Actinobacteria | Actinomycetales | Frankia | Frankia sp. CcI3 | 0.07 |
| Actinobacteria | Actinomycetales | Frankia | Frankia sp. EAN1pec | 0.07 |
| Actinobacteria | Actinomycetales | Frankia | Frankia sp. EUN1f | 0.02 |
| Actinobacteria | Actinomycetales | Frankia | Frankia sp. EuI1c | 0.04 |
| Actinobacteria | Actinomycetales | Frankia | Frankia symbiont of Datisca glomerata | 0.02 |
| Actinobacteria | Actinomycetales | Geodermatophilus | Geodermatophilus obscurus | 0.09 |
| Actinobacteria | Actinomycetales | Stackebrandtia | Stackebrandtia nassauensis | 0.12 |
| Actinobacteria | Actinomycetales | Gordonia | Gordonia bronchialis | 0.15 |
| Actinobacteria | Actinomycetales | Intrasporangium | Intrasporangium calvum | 0.06 |
| Actinobacteria | Actinomycetales | Janibacter | Janibacter sp. HTCC2649 | 0.10 |
| Actinobacteria | Actinomycetales | Jonesia | Jonesia denitrificans | 0.12 |
| Actinobacteria | Actinomycetales | Kineococcus | Kineococcus radiotolerans | 0.33 |
| Actinobacteria | Actinomycetales | Clavibacter | Clavibacter michiganensis | 0.32 |
| Actinobacteria | Actinomycetales | Leifsonia | Leifsonia xyli | 0.16 |
| Actinobacteria | Actinomycetales | Arthrobacter | Arthrobacter arilaitensis | 0.19 |
| Actinobacteria | Actinomycetales | Arthrobacter | Arthrobacter aurescens | 0.38 |
| Actinobacteria | Actinomycetales | Arthrobacter | Arthrobacter chlorophenolicus | 0.16 |
| Actinobacteria | Actinomycetales | Arthrobacter | Arthrobacter phenanthrenivorans | 0.07 |
| Actinobacteria | Actinomycetales | Arthrobacter | Arthrobacter sp. | 0.21 |
| Actinobacteria | Actinomycetales | Arthrobacter | Arthrobacter sp. FB24 | 0.41 |
| Actinobacteria | Actinomycetales | Kocuria | Kocuria rhizophila | 0.23 |
| Actinobacteria | Actinomycetales | Micrococcus | Micrococcus luteus | 0.24 |
| Actinobacteria | Actinomycetales | Renibacterium | Renibacterium salmoninarum | 0.20 |
| Actinobacteria | Actinomycetales | Rothia | Rothia dentocariosa | 0.08 |
| Actinobacteria | Actinomycetales | Rothia | Rothia mucilaginosa | 0.08 |
| Actinobacteria | Actinomycetales | Micromonospora | Micromonospora aurantiaca | 0.06 |
| Actinobacteria | Actinomycetales | Micromonospora | Micromonospora sp. | 0.05 |
| Actinobacteria | Actinomycetales | Micromonospora | Micromonospora sp. ATCC 39149 | 0.03 |
| Actinobacteria | Actinomycetales | Micromonospora | Micromonospora sp. L5 | 0.05 |
| Actinobacteria | Actinomycetales | Salinispora | Salinispora arenicola | 0.03 |
| Actinobacteria | Actinomycetales | Salinispora | Salinispora tropica | 0.12 |
| Actinobacteria | Actinomycetales | Mycobacterium | Mycobacterium abscessus | 0.05 |
| Actinobacteria | Actinomycetales | Mycobacterium | Mycobacterium avium | 0.12 |
| Actinobacteria | Actinomycetales | Mycobacterium | Mycobacterium bovis | 0.05 |
| Actinobacteria | Actinomycetales | Mycobacterium | Mycobacterium gilvum | 0.09 |
| Actinobacteria | Actinomycetales | Mycobacterium | Mycobacterium intracellulare | 0.03 |
| Actinobacteria | Actinomycetales | Mycobacterium | Mycobacterium kansasii | 0.02 |
| Actinobacteria | Actinomycetales | Mycobacterium | Mycobacterium leprae | 0.03 |
| Actinobacteria | Actinomycetales | Mycobacterium | Mycobacterium marinum | 0.05 |
| Actinobacteria | Actinomycetales | Mycobacterium | Mycobacterium microti | 0.01 |
| Actinobacteria | Actinomycetales | Mycobacterium | Mycobacterium parascrofulaceum | 0.02 |
| Actinobacteria | Actinomycetales | Mycobacterium | Mycobacterium smegmatis | 0.19 |
| Actinobacteria | Actinomycetales | Mycobacterium | Mycobacterium sp. | 0.07 |
| Actinobacteria | Actinomycetales | Mycobacterium | Mycobacterium sp. JLS | 0.10 |
| Actinobacteria | Actinomycetales | Mycobacterium | Mycobacterium sp. KMS | 0.07 |
| Actinobacteria | Actinomycetales | Mycobacterium | Mycobacterium sp. MCS | 0.08 |
| Actinobacteria | Actinomycetales | Mycobacterium | Mycobacterium sp. Spyr1 | 0.02 |
| Actinobacteria | Actinomycetales | Mycobacterium | Mycobacterium tuberculosis | 0.11 |
| Actinobacteria | Actinomycetales | Mycobacterium | Mycobacterium ulcerans | 0.05 |
| Actinobacteria | Actinomycetales | Mycobacterium | Mycobacterium vanbaalenii | 0.12 |
| Actinobacteria | Actinomycetales | Nakamurella | Nakamurella multipartita | 0.08 |
| Actinobacteria | Actinomycetales | Nocardia | Nocardia farcinica | 0.22 |
| Actinobacteria | Actinomycetales | Rhodococcus | Rhodococcus equi | 0.12 |
| Actinobacteria | Actinomycetales | Rhodococcus | Rhodococcus erythropolis | 0.19 |
| Actinobacteria | Actinomycetales | Rhodococcus | Rhodococcus jostii | 0.33 |
| Actinobacteria | Actinomycetales | Rhodococcus | Rhodococcus opacus | 0.07 |
| Actinobacteria | Actinomycetales | Aeromicrobium | Aeromicrobium marinum | 0.02 |
| Actinobacteria | Actinomycetales | Kribbella | Kribbella flavida | 0.13 |
| Actinobacteria | Actinomycetales | Nocardioides | Nocardioides sp. | 0.07 |
| Actinobacteria | Actinomycetales | Nocardioides | Nocardioides sp. JS614 | 0.17 |
| Actinobacteria | Actinomycetales | Nocardiopsis | Nocardiopsis dassonvillei | 0.16 |
| Actinobacteria | Actinomycetales | Thermobifida | Thermobifida fusca | 0.15 |
| Actinobacteria | Actinomycetales | Xylanimonas | Xylanimonas cellulosilytica | 0.15 |
| Actinobacteria | Actinomycetales | Propionibacterium | Propionibacterium acidifaciens | 0.04 |
| Actinobacteria | Actinomycetales | Propionibacterium | Propionibacterium acnes | 0.12 |
| Actinobacteria | Actinomycetales | Propionibacterium | Propionibacterium freudenreichii | 0.02 |
| Actinobacteria | Actinomycetales | Amycolatopsis | Amycolatopsis mediterranei | 0.09 |
| Actinobacteria | Actinomycetales | Saccharomonospora | Saccharomonospora viridis | 0.08 |
| Actinobacteria | Actinomycetales | Saccharopolyspora | Saccharopolyspora erythraea | 0.28 |
| Actinobacteria | Actinomycetales | Thermobispora | Thermobispora bispora | 0.07 |
| Actinobacteria | Actinomycetales | Sanguibacter | Sanguibacter keddieii | 0.15 |
| Actinobacteria | Actinomycetales | Segniliparus | Segniliparus rotundus | 0.04 |
| Actinobacteria | Actinomycetales | Segniliparus | Segniliparus rugosus | 0.01 |
| Actinobacteria | Actinomycetales | Kitasatospora | Kitasatospora setae | 0.03 |
| Actinobacteria | Actinomycetales | Streptomyces | Streptomyces albus | 0.08 |
| Actinobacteria | Actinomycetales | Streptomyces | Streptomyces ambofaciens | 0.01 |
| Actinobacteria | Actinomycetales | Streptomyces | Streptomyces avermitilis | 0.40 |
| Actinobacteria | Actinomycetales | Streptomyces | Streptomyces bingchenggensis | 0.12 |
| Actinobacteria | Actinomycetales | Streptomyces | Streptomyces clavuligerus | 0.09 |
| Actinobacteria | Actinomycetales | Streptomyces | Streptomyces coelicolor | 0.45 |
| Actinobacteria | Actinomycetales | Streptomyces | Streptomyces filamentosus | 0.10 |
| Actinobacteria | Actinomycetales | Streptomyces | Streptomyces flavogriseus | 0.08 |
| Actinobacteria | Actinomycetales | Streptomyces | Streptomyces ghanaensis | 0.10 |
| Actinobacteria | Actinomycetales | Streptomyces | Streptomyces griseoflavus | 0.09 |
| Actinobacteria | Actinomycetales | Streptomyces | Streptomyces griseus | 0.28 |
| Actinobacteria | Actinomycetales | Streptomyces | Streptomyces hygroscopicus | 0.15 |
| Actinobacteria | Actinomycetales | Streptomyces | Streptomyces lividans | 0.14 |
| Actinobacteria | Actinomycetales | Streptomyces | Streptomyces pristinaespiralis | 0.11 |
| Actinobacteria | Actinomycetales | Streptomyces | Streptomyces scabiei | 0.25 |
| Actinobacteria | Actinomycetales | Streptomyces | Streptomyces sp. | 0.37 |
| Actinobacteria | Actinomycetales | Streptomyces | Streptomyces sp. AA4 | 0.08 |
| Actinobacteria | Actinomycetales | Streptomyces | Streptomyces sp. ACT-1 | 0.08 |
| Actinobacteria | Actinomycetales | Streptomyces | Streptomyces sp. ACTE | 0.07 |
| Actinobacteria | Actinomycetales | Streptomyces | Streptomyces sp. C | 0.06 |
| Actinobacteria | Actinomycetales | Streptomyces | Streptomyces sp. Mg1 | 0.06 |
| Actinobacteria | Actinomycetales | Streptomyces | Streptomyces sp. SPB74 | 0.05 |
| Actinobacteria | Actinomycetales | Streptomyces | Streptomyces sp. SPB78 | 0.06 |
| Actinobacteria | Actinomycetales | Streptomyces | Streptomyces sp. e14 | 0.06 |
| Actinobacteria | Actinomycetales | Streptomyces | Streptomyces sviceus | 0.09 |
| Actinobacteria | Actinomycetales | Streptomyces | Streptomyces violaceusniger | 0.10 |
| Actinobacteria | Actinomycetales | Streptomyces | Streptomyces viridochromogenes | 0.12 |
| Actinobacteria | Actinomycetales | Streptosporangium | Streptosporangium roseum | 0.14 |
| Actinobacteria | Actinomycetales | Thermomonospora | Thermomonospora curvata | 0.09 |
| Actinobacteria | Actinomycetales | Tsukamurella | Tsukamurella paurometabola | 0.10 |
| Actinobacteria | Actinomycetales | Tropheryma | Tropheryma whipplei | 0.02 |
| Actinobacteria | Bifidobacteriales | Bifidobacterium | Bifidobacterium adolescentis | 0.17 |
| Actinobacteria | Bifidobacteriales | Bifidobacterium | Bifidobacterium angulatum | 0.03 |
| Actinobacteria | Bifidobacteriales | Bifidobacterium | Bifidobacterium animalis | 0.05 |
| Actinobacteria | Bifidobacteriales | Bifidobacterium | Bifidobacterium bifidum | 0.07 |
| Actinobacteria | Bifidobacteriales | Bifidobacterium | Bifidobacterium breve | 0.02 |
| Actinobacteria | Bifidobacteriales | Bifidobacterium | Bifidobacterium catenulatum | 0.01 |
| Actinobacteria | Bifidobacteriales | Bifidobacterium | Bifidobacterium dentium | 0.05 |
| Actinobacteria | Bifidobacteriales | Bifidobacterium | Bifidobacterium gallicum | 0.01 |
| Actinobacteria | Bifidobacteriales | Bifidobacterium | Bifidobacterium longum | 0.28 |
| Actinobacteria | Bifidobacteriales | Bifidobacterium | Bifidobacterium pseudocatenulatum | 0.02 |
| Actinobacteria | Bifidobacteriales | Bifidobacterium | Bifidobacterium sp. 12_1_47BFAA | 0.02 |
| Actinobacteria | Bifidobacteriales | Gardnerella | Gardnerella vaginalis | 0.03 |
| Actinobacteria | Bifidobacteriales | Scardovia | Scardovia inopinata | 0.01 |
| Actinobacteria | Coriobacteriales | Atopobium | Atopobium parvulum | 0.12 |
| Actinobacteria | Coriobacteriales | Atopobium | Atopobium rimae | 0.04 |
| Actinobacteria | Coriobacteriales | Atopobium | Atopobium vaginae | 0.04 |
| Actinobacteria | Coriobacteriales | Collinsella | Collinsella aerofaciens | 0.08 |
| Actinobacteria | Coriobacteriales | Collinsella | Collinsella intestinalis | 0.11 |
| Actinobacteria | Coriobacteriales | Collinsella | Collinsella stercoris | 0.09 |
| Actinobacteria | Coriobacteriales | Cryptobacterium | Cryptobacterium curtum | 0.07 |
| Actinobacteria | Coriobacteriales | Eggerthella | Eggerthella lenta | 0.16 |
| Actinobacteria | Coriobacteriales | Eggerthella | Eggerthella sp. 1_3_56FAA | 0.02 |
| Actinobacteria | Coriobacteriales | Gordonibacter | Gordonibacter pamelaeae | 0.06 |
| Actinobacteria | Coriobacteriales | Olsenella | Olsenella uli | 0.10 |
| Actinobacteria | Coriobacteriales | Slackia | Slackia exigua | 0.05 |
| Actinobacteria | Coriobacteriales | Slackia | Slackia heliotrinireducens | 0.12 |
| Actinobacteria | Rubrobacterales | Rubrobacter | Rubrobacter xylanophilus | 0.09 |
| Actinobacteria | Solirubrobacterales | Conexibacter | Conexibacter woesei | 0.05 |
| Actinobacteria | unclassified (derived from Actinobacteria (class)) | unclassified (derived from Actinobacteria (class)) | marine actinobacterium PHSC20C1 | 0.09 |
| Aquificae | Aquificales | Aquifex | Aquifex aeolicus | 0.02 |
| Aquificae | Aquificales | Thermovibrio | Thermovibrio ammonificans | 0.01 |
| Aquificae | Aquificales | Sulfurihydrogenibium | Sulfurihydrogenibium azorense | 0.01 |
| Aquificae | Aquificales | Sulfurihydrogenibium | Sulfurihydrogenibium sp. YO3AOP1 | 0.01 |
| Aquificae | Aquificales | Sulfurihydrogenibium | Sulfurihydrogenibium yellowstonense | 0.01 |
| Bacteroidetes | Bacteroidales | Bacteroides | Bacteroides caccae | 0.93 |
| Bacteroidetes | Bacteroidales | Bacteroides | Bacteroides capillosus | 0.16 |
| Bacteroidetes | Bacteroidales | Bacteroides | Bacteroides cellulosilyticus | 3.00 |
| Bacteroidetes | Bacteroidales | Bacteroides | Bacteroides coprocola | 0.66 |
| Bacteroidetes | Bacteroidales | Bacteroides | Bacteroides coprophilus | 0.47 |
| Bacteroidetes | Bacteroidales | Bacteroides | Bacteroides dorei | 0.93 |
| Bacteroidetes | Bacteroidales | Bacteroides | Bacteroides eggerthii | 1.23 |
| Bacteroidetes | Bacteroidales | Bacteroides | Bacteroides finegoldii | 0.90 |
| Bacteroidetes | Bacteroidales | Bacteroides | Bacteroides fragilis | 5.79 |
| Bacteroidetes | Bacteroidales | Bacteroides | Bacteroides helcogenes | 1.97 |
| Bacteroidetes | Bacteroidales | Bacteroides | Bacteroides intestinalis | 2.08 |
| Bacteroidetes | Bacteroidales | Bacteroides | Bacteroides ovatus | 2.22 |
| Bacteroidetes | Bacteroidales | Bacteroides | Bacteroides pectinophilus | 0.04 |
| Bacteroidetes | Bacteroidales | Bacteroides | Bacteroides plebeius | 0.67 |
| Bacteroidetes | Bacteroidales | Bacteroides | Bacteroides sp. | 7.64 |
| Bacteroidetes | Bacteroidales | Bacteroides | Bacteroides sp. 1_1_14 | 0.66 |
| Bacteroidetes | Bacteroidales | Bacteroides | Bacteroides sp. 1_1_6 | 1.05 |
| Bacteroidetes | Bacteroidales | Bacteroides | Bacteroides sp. 20_3 | 0.34 |
| Bacteroidetes | Bacteroidales | Bacteroides | Bacteroides sp. 2_1_16 | 1.02 |
| Bacteroidetes | Bacteroidales | Bacteroides | Bacteroides sp. 2_1_22 | 0.74 |
| Bacteroidetes | Bacteroidales | Bacteroides | Bacteroides sp. 2_1_33B | 0.34 |
| Bacteroidetes | Bacteroidales | Bacteroides | Bacteroides sp. 2_1_7 | 0.35 |
| Bacteroidetes | Bacteroidales | Bacteroides | Bacteroides sp. 2_2_4 | 0.80 |
| Bacteroidetes | Bacteroidales | Bacteroides | Bacteroides sp. 3_1_19 | 0.40 |
| Bacteroidetes | Bacteroidales | Bacteroides | Bacteroides sp. 3_1_23 | 0.45 |
| Bacteroidetes | Bacteroidales | Bacteroides | Bacteroides sp. 3_1_33FAA | 0.49 |
| Bacteroidetes | Bacteroidales | Bacteroides | Bacteroides sp. 3_1_40A | 0.51 |
| Bacteroidetes | Bacteroidales | Bacteroides | Bacteroides sp. 3_2_5 | 1.10 |
| Bacteroidetes | Bacteroidales | Bacteroides | Bacteroides sp. 4_1_36 | 0.62 |
| Bacteroidetes | Bacteroidales | Bacteroides | Bacteroides sp. 4_3_47FAA | 0.57 |
| Bacteroidetes | Bacteroidales | Bacteroides | Bacteroides sp. 9_1_42FAA | 0.46 |
| Bacteroidetes | Bacteroidales | Bacteroides | Bacteroides sp. D1 | 0.68 |
| Bacteroidetes | Bacteroidales | Bacteroides | Bacteroides sp. D2 | 0.73 |
| Bacteroidetes | Bacteroidales | Bacteroides | Bacteroides sp. D20 | 0.99 |
| Bacteroidetes | Bacteroidales | Bacteroides | Bacteroides sp. D22 | 0.43 |
| Bacteroidetes | Bacteroidales | Bacteroides | Bacteroides stercoris | 1.05 |
| Bacteroidetes | Bacteroidales | Bacteroides | Bacteroides thetaiotaomicron | 3.45 |
| Bacteroidetes | Bacteroidales | Bacteroides | Bacteroides uniformis | 1.28 |
| Bacteroidetes | Bacteroidales | Bacteroides | Bacteroides vulgatus | 3.23 |
| Bacteroidetes | Bacteroidales | Bacteroides | Bacteroides xylanisolvens | 1.14 |
| Bacteroidetes | Bacteroidales | Paludibacter | Paludibacter propionicigenes | 0.28 |
| Bacteroidetes | Bacteroidales | Parabacteroides | Parabacteroides distasonis | 2.09 |
| Bacteroidetes | Bacteroidales | Parabacteroides | Parabacteroides johnsonii | 0.55 |
| Bacteroidetes | Bacteroidales | Parabacteroides | Parabacteroides merdae | 0.55 |
| Bacteroidetes | Bacteroidales | Parabacteroides | Parabacteroides sp. D13 | 0.50 |
| Bacteroidetes | Bacteroidales | Porphyromonas | Porphyromonas asaccharolytica | 0.03 |
| Bacteroidetes | Bacteroidales | Porphyromonas | Porphyromonas endodontalis | 0.05 |
| Bacteroidetes | Bacteroidales | Porphyromonas | Porphyromonas gingivalis | 0.39 |
| Bacteroidetes | Bacteroidales | Porphyromonas | Porphyromonas uenonis | 0.05 |
| Bacteroidetes | Bacteroidales | Prevotella | Prevotella amnii | 0.09 |
| Bacteroidetes | Bacteroidales | Prevotella | Prevotella bergensis | 0.17 |
| Bacteroidetes | Bacteroidales | Prevotella | Prevotella bivia | 0.10 |
| Bacteroidetes | Bacteroidales | Prevotella | Prevotella bryantii | 0.18 |
| Bacteroidetes | Bacteroidales | Prevotella | Prevotella buccae | 0.30 |
| Bacteroidetes | Bacteroidales | Prevotella | Prevotella buccalis | 0.22 |
| Bacteroidetes | Bacteroidales | Prevotella | Prevotella copri | 0.27 |
| Bacteroidetes | Bacteroidales | Prevotella | Prevotella disiens | 0.12 |
| Bacteroidetes | Bacteroidales | Prevotella | Prevotella marshii | 0.17 |
| Bacteroidetes | Bacteroidales | Prevotella | Prevotella melaninogenica | 0.63 |
| Bacteroidetes | Bacteroidales | Prevotella | Prevotella oralis | 0.13 |
| Bacteroidetes | Bacteroidales | Prevotella | Prevotella oris | 0.32 |
| Bacteroidetes | Bacteroidales | Prevotella | Prevotella ruminicola | 0.71 |
| Bacteroidetes | Bacteroidales | Prevotella | Prevotella salivae | 0.10 |
| Bacteroidetes | Bacteroidales | Prevotella | Prevotella sp. | 0.37 |
| Bacteroidetes | Bacteroidales | Prevotella | Prevotella sp. oral taxon 299 | 0.09 |
| Bacteroidetes | Bacteroidales | Prevotella | Prevotella sp. oral taxon 317 | 0.16 |
| Bacteroidetes | Bacteroidales | Prevotella | Prevotella sp. oral taxon 472 | 0.17 |
| Bacteroidetes | Bacteroidales | Prevotella | Prevotella tannerae | 0.09 |
| Bacteroidetes | Bacteroidales | Prevotella | Prevotella timonensis | 0.18 |
| Bacteroidetes | Bacteroidales | Prevotella | Prevotella veroralis | 0.16 |
| Bacteroidetes | Bacteroidales | Alistipes | Alistipes putredinis | 0.26 |
| Bacteroidetes | Bacteroidales | Alistipes | Alistipes shahii | 0.30 |
| Bacteroidetes | Bacteroidales | Alistipes | Alistipes sp. HGB5 | 0.26 |
| Bacteroidetes | Bacteroidales | Candidatus Azobacteroides | Candidatus Azobacteroides pseudotrichonymphae | 0.02 |
| Bacteroidetes | Cytophagales | Algoriphagus | Algoriphagus sp. PR1 | 0.04 |
| Bacteroidetes | Cytophagales | Cytophaga | Cytophaga hutchinsonii | 0.11 |
| Bacteroidetes | Cytophagales | Dyadobacter | Dyadobacter fermentans | 0.26 |
| Bacteroidetes | Cytophagales | Leadbetterella | Leadbetterella byssophila | 0.12 |
| Bacteroidetes | Cytophagales | Microscilla | Microscilla marina | 0.03 |
| Bacteroidetes | Cytophagales | Spirosoma | Spirosoma linguale | 0.23 |
| Bacteroidetes | Cytophagales | Marivirga | Marivirga tractuosa | 0.04 |
| Bacteroidetes | Flavobacteriales | Capnocytophaga | Capnocytophaga gingivalis | 0.03 |
| Bacteroidetes | Flavobacteriales | Capnocytophaga | Capnocytophaga ochracea | 0.10 |
| Bacteroidetes | Flavobacteriales | Capnocytophaga | Capnocytophaga sputigena | 0.03 |
| Bacteroidetes | Flavobacteriales | Cellulophaga | Cellulophaga algicola | 0.07 |
| Bacteroidetes | Flavobacteriales | Chryseobacterium | Chryseobacterium gleum | 0.07 |
| Bacteroidetes | Flavobacteriales | Croceibacter | Croceibacter atlanticus | 0.05 |
| Bacteroidetes | Flavobacteriales | Dokdonia | Dokdonia donghaensis | 0.05 |
| Bacteroidetes | Flavobacteriales | Flavobacterium | Flavobacterium johnsoniae | 0.31 |
| Bacteroidetes | Flavobacteriales | Flavobacterium | Flavobacterium psychrophilum | 0.11 |
| Bacteroidetes | Flavobacteriales | Gramella | Gramella forsetii | 0.16 |
| Bacteroidetes | Flavobacteriales | Kordia | Kordia algicida | 0.03 |
| Bacteroidetes | Flavobacteriales | Leeuwenhoekiella | Leeuwenhoekiella blandensis | 0.11 |
| Bacteroidetes | Flavobacteriales | Maribacter | Maribacter sp. HTCC2170 | 0.11 |
| Bacteroidetes | Flavobacteriales | Polaribacter | Polaribacter irgensii | 0.04 |
| Bacteroidetes | Flavobacteriales | Polaribacter | Polaribacter sp. MED152 | 0.04 |
| Bacteroidetes | Flavobacteriales | Psychroflexus | Psychroflexus torquis | 0.03 |
| Bacteroidetes | Flavobacteriales | Riemerella | Riemerella anatipestifer | 0.04 |
| Bacteroidetes | Flavobacteriales | Robiginitalea | Robiginitalea biformata | 0.06 |
| Bacteroidetes | Flavobacteriales | Weeksella | Weeksella virosa | 0.01 |
| Bacteroidetes | Flavobacteriales | Zunongwangia | Zunongwangia profunda | 0.14 |
| Bacteroidetes | Flavobacteriales | unclassified (derived from Flavobacteriaceae) | Flavobacteriaceae bacterium 3519-10 | 0.04 |
| Bacteroidetes | Flavobacteriales | unclassified (derived from Flavobacteriales) | Flavobacteria bacterium BAL38 | 0.02 |
| Bacteroidetes | Flavobacteriales | unclassified (derived from Flavobacteriales) | Flavobacteriales bacterium ALC-1 | 0.02 |
| Bacteroidetes | unclassified (derived from Flavobacteria) | unclassified (derived from Flavobacteria) | Flavobacteria bacterium BBFL7 | 0.01 |
| Bacteroidetes | unclassified (derived from Flavobacteria) | unclassified (derived from Flavobacteria) | Flavobacteria bacterium MS024-2A | 0.01 |
| Bacteroidetes | unclassified (derived from Flavobacteria) | unclassified (derived from Flavobacteria) | Flavobacteria bacterium MS024-3C | 0.01 |
| Bacteroidetes | Sphingobacteriales | Rhodothermus | Rhodothermus marinus | 0.06 |
| Bacteroidetes | Sphingobacteriales | Salinibacter | Salinibacter ruber | 0.02 |
| Bacteroidetes | Sphingobacteriales | Mucilaginibacter | Mucilaginibacter paludis | 0.06 |
| Bacteroidetes | Sphingobacteriales | Pedobacter | Pedobacter heparinus | 0.31 |
| Bacteroidetes | Sphingobacteriales | Pedobacter | Pedobacter saltans | 0.07 |
| Bacteroidetes | Sphingobacteriales | Pedobacter | Pedobacter sp. BAL39 | 0.07 |
| Bacteroidetes | Sphingobacteriales | Sphingobacterium | Sphingobacterium spiritivorum | 0.16 |
| Bacteroidetes | Sphingobacteriales | Chitinophaga | Chitinophaga pinensis | 0.30 |
| Bacteroidetes | unclassified (derived from Bacteroidetes) | Candidatus Amoebophilus | Candidatus Amoebophilus asiaticus | 0.01 |
| Bacteroidetes | unclassified (derived from Bacteroidetes) | unclassified (derived from Bacteroidetes) | Bacteroidetes oral taxon 274 | 0.04 |
| Bacteroidetes | unclassified (derived from Bacteroidetes) | unclassified (derived from Bacteroidetes) | unidentified eubacterium SCB49 | 0.02 |
| Chlamydiae | Chlamydiales | Candidatus Protochlamydia | Candidatus Protochlamydia amoebophila | 0.01 |
| Chlamydiae | Chlamydiales | Parachlamydia | Parachlamydia acanthamoebae | 0.01 |
| Chlorobi | Chlorobiales | Chlorobaculum | Chlorobaculum parvum | 0.01 |
| Chlorobi | Chlorobiales | Chlorobaculum | Chlorobaculum tepidum | 0.03 |
| Chlorobi | Chlorobiales | Chlorobium | Chlorobium chlorochromatii | 0.02 |
| Chlorobi | Chlorobiales | Chlorobium | Chlorobium ferrooxidans | 0.01 |
| Chlorobi | Chlorobiales | Chlorobium | Chlorobium limicola | 0.02 |
| Chlorobi | Chlorobiales | Chlorobium | Chlorobium phaeobacteroides | 0.04 |
| Chlorobi | Chlorobiales | Chlorobium | Chlorobium phaeovibrioides | 0.01 |
| Chlorobi | Chlorobiales | Chloroherpeton | Chloroherpeton thalassium | 0.03 |
| Chlorobi | Chlorobiales | Pelodictyon | Pelodictyon luteolum | 0.02 |
| Chlorobi | Chlorobiales | Pelodictyon | Pelodictyon phaeoclathratiforme | 0.03 |
| Chloroflexi | Anaerolineales | Anaerolinea | Anaerolinea thermophila | 0.02 |
| Chloroflexi | Chloroflexales | Chloroflexus | Chloroflexus aggregans | 0.02 |
| Chloroflexi | Chloroflexales | Chloroflexus | Chloroflexus aurantiacus | 0.03 |
| Chloroflexi | Chloroflexales | Chloroflexus | Chloroflexus sp. Y-400-fl | 0.02 |
| Chloroflexi | Chloroflexales | Roseiflexus | Roseiflexus castenholzii | 0.03 |
| Chloroflexi | Chloroflexales | Roseiflexus | Roseiflexus sp. RS-1 | 0.04 |
| Chloroflexi | Chloroflexales | Oscillochloris | Oscillochloris trichoides | 0.01 |
| Chloroflexi | Herpetosiphonales | Herpetosiphon | Herpetosiphon aurantiacus | 0.02 |
| Chloroflexi | unclassified (derived from Dehalococcoidetes) | Dehalococcoides | Dehalococcoides ethenogenes | 0.01 |
| Chloroflexi | Ktedonobacterales | Ktedonobacter | Ktedonobacter racemifer | 0.02 |
| Chloroflexi | Sphaerobacterales | Sphaerobacter | Sphaerobacter thermophilus | 0.04 |
| Chrysiogenetes | Chrysiogenales | Desulfurispirillum | Desulfurispirillum indicum | 0.02 |
| Cyanobacteria | Gloeobacterales | Gloeobacter | Gloeobacter violaceus | 0.02 |
| Cyanobacteria | Chroococcales | Crocosphaera | Crocosphaera watsonii | 0.01 |
| Cyanobacteria | Chroococcales | Cyanothece | Cyanothece sp. ATCC 51142 | 0.01 |
| Cyanobacteria | Chroococcales | Cyanothece | Cyanothece sp. CCY0110 | 0.01 |
| Cyanobacteria | Chroococcales | Cyanothece | Cyanothece sp. PCC 7424 | 0.01 |
| Cyanobacteria | Chroococcales | Cyanothece | Cyanothece sp. PCC 7425 | 0.01 |
| Cyanobacteria | Chroococcales | Cyanothece | Cyanothece sp. PCC 7822 | 0.01 |
| Cyanobacteria | Chroococcales | Cyanothece | Cyanothece sp. PCC 8801 | 0.01 |
| Cyanobacteria | Chroococcales | Cyanothece | Cyanothece sp. PCC 8802 | 0.01 |
| Cyanobacteria | Chroococcales | Microcystis | Microcystis aeruginosa | 0.01 |
| Cyanobacteria | Chroococcales | Synechococcus | Synechococcus elongatus | 0.01 |
| Cyanobacteria | Chroococcales | Synechococcus | Synechococcus sp. | 0.05 |
| Cyanobacteria | Chroococcales | Synechococcus | Synechococcus sp. CC9311 | 0.01 |
| Cyanobacteria | Chroococcales | Synechococcus | Synechococcus sp. JA-2-3B&#39;a(2-13) | 0.01 |
| Cyanobacteria | Chroococcales | Synechococcus | Synechococcus sp. JA-3-3Ab | 0.02 |
| Cyanobacteria | Chroococcales | Synechococcus | Synechococcus sp. RS9917 | 0.01 |
| Cyanobacteria | Chroococcales | Synechococcus | Synechococcus sp. WH 5701 | 0.01 |
| Cyanobacteria | Chroococcales | Synechococcus | Synechococcus sp. WH 8102 | 0.01 |
| Cyanobacteria | Chroococcales | Synechocystis | Synechocystis sp. | 0.01 |
| Cyanobacteria | Chroococcales | Synechocystis | Synechocystis sp. PCC 6803 | 0.01 |
| Cyanobacteria | Chroococcales | Thermosynechococcus | Thermosynechococcus elongatus | 0.01 |
| Cyanobacteria | Nostocales | Anabaena | Anabaena sp. | 0.01 |
| Cyanobacteria | Nostocales | Anabaena | Anabaena variabilis | 0.02 |
| Cyanobacteria | Nostocales | Nodularia | Nodularia spumigena | 0.01 |
| Cyanobacteria | Nostocales | Nostoc | Nostoc punctiforme | 0.02 |
| Cyanobacteria | Nostocales | Nostoc | Nostoc sp. | 0.02 |
| Cyanobacteria | Nostocales | Nostoc | Nostoc sp. PCC 7120 | 0.03 |
| Cyanobacteria | Oscillatoriales | Microcoleus | Microcoleus chthonoplastes | 0.01 |
| Cyanobacteria | Oscillatoriales | Trichodesmium | Trichodesmium erythraeum | 0.03 |
| Cyanobacteria | Prochlorales | Prochlorococcus | Prochlorococcus marinus | 0.02 |
| Cyanobacteria | unclassified (derived from Cyanobacteria) | Acaryochloris | Acaryochloris marina | 0.01 |
| Deferribacteres | Deferribacterales | Calditerrivibrio | Calditerrivibrio nitroreducens | 0.01 |
| Deferribacteres | Deferribacterales | Deferribacter | Deferribacter desulfuricans | 0.01 |
| Deferribacteres | Deferribacterales | Denitrovibrio | Denitrovibrio acetiphilus | 0.03 |
| Deinococcus-Thermus | Deinococcales | Deinococcus | Deinococcus deserti | 0.01 |
| Deinococcus-Thermus | Deinococcales | Deinococcus | Deinococcus geothermalis | 0.03 |
| Deinococcus-Thermus | Deinococcales | Deinococcus | Deinococcus maricopensis | 0.01 |
| Deinococcus-Thermus | Deinococcales | Deinococcus | Deinococcus radiodurans | 0.02 |
| Deinococcus-Thermus | Deinococcales | Truepera | Truepera radiovictrix | 0.01 |
| Deinococcus-Thermus | Thermales | Meiothermus | Meiothermus ruber | 0.01 |
| Deinococcus-Thermus | Thermales | Meiothermus | Meiothermus silvanus | 0.02 |
| Deinococcus-Thermus | Thermales | Oceanithermus | Oceanithermus profundus | 0.01 |
| Deinococcus-Thermus | Thermales | Thermus | Thermus thermophilus | 0.02 |
| Dictyoglomi | Dictyoglomales | Dictyoglomus | Dictyoglomus thermophilum | 0.01 |
| Dictyoglomi | Dictyoglomales | Dictyoglomus | Dictyoglomus turgidum | 0.01 |
| Elusimicrobia | Elusimicrobiales | Elusimicrobium | Elusimicrobium minutum | 0.01 |
| Fibrobacteres | Fibrobacterales | Fibrobacter | Fibrobacter succinogenes | 0.04 |
| Firmicutes | Bacillales | Alicyclobacillus | Alicyclobacillus acidocaldarius | 0.03 |
| Firmicutes | Bacillales | unclassified (derived from Alicyclobacillaceae) | Bacillus tusciae | 0.02 |
| Firmicutes | Bacillales | Anoxybacillus | Anoxybacillus flavithermus | 0.06 |
| Firmicutes | Bacillales | Bacillus | Bacillus amyloliquefaciens | 0.06 |
| Firmicutes | Bacillales | Bacillus | Bacillus anthracis | 0.05 |
| Firmicutes | Bacillales | Bacillus | Bacillus atrophaeus | 0.01 |
| Firmicutes | Bacillales | Bacillus | Bacillus cellulosilyticus | 0.04 |
| Firmicutes | Bacillales | Bacillus | Bacillus cereus | 0.23 |
| Firmicutes | Bacillales | Bacillus | Bacillus clausii | 0.09 |
| Firmicutes | Bacillales | Bacillus | Bacillus coagulans | 0.03 |
| Firmicutes | Bacillales | Bacillus | Bacillus coahuilensis | 0.01 |
| Firmicutes | Bacillales | Bacillus | Bacillus cytotoxicus | 0.04 |
| Firmicutes | Bacillales | Bacillus | Bacillus halodurans | 0.07 |
| Firmicutes | Bacillales | Bacillus | Bacillus licheniformis | 0.10 |
| Firmicutes | Bacillales | Bacillus | Bacillus megaterium | 0.06 |
| Firmicutes | Bacillales | Bacillus | Bacillus mycoides | 0.02 |
| Firmicutes | Bacillales | Bacillus | Bacillus pseudofirmus | 0.04 |
| Firmicutes | Bacillales | Bacillus | Bacillus pseudomycoides | 0.01 |
| Firmicutes | Bacillales | Bacillus | Bacillus pumilus | 0.08 |
| Firmicutes | Bacillales | Bacillus | Bacillus selenitireducens | 0.02 |
| Firmicutes | Bacillales | Bacillus | Bacillus sp. | 0.08 |
| Firmicutes | Bacillales | Bacillus | Bacillus sp. 2_A_57_CT2 | 0.02 |
| Firmicutes | Bacillales | Bacillus | Bacillus sp. B14905 | 0.02 |
| Firmicutes | Bacillales | Bacillus | Bacillus sp. BT1B_CT2 | 0.02 |
| Firmicutes | Bacillales | Bacillus | Bacillus sp. NRRL B-14911 | 0.06 |
| Firmicutes | Bacillales | Bacillus | Bacillus sp. SG-1 | 0.02 |
| Firmicutes | Bacillales | Bacillus | Bacillus sp. m3-13 | 0.01 |
| Firmicutes | Bacillales | Bacillus | Bacillus subtilis | 0.19 |
| Firmicutes | Bacillales | Bacillus | Bacillus thuringiensis | 0.11 |
| Firmicutes | Bacillales | Bacillus | Bacillus weihenstephanensis | 0.02 |
| Firmicutes | Bacillales | Geobacillus | Geobacillus kaustophilus | 0.07 |
| Firmicutes | Bacillales | Geobacillus | Geobacillus sp. C56-T3 | 0.02 |
| Firmicutes | Bacillales | Geobacillus | Geobacillus sp. G11MC16 | 0.02 |
| Firmicutes | Bacillales | Geobacillus | Geobacillus sp. WCH70 | 0.03 |
| Firmicutes | Bacillales | Geobacillus | Geobacillus sp. Y4.1MC1 | 0.03 |
| Firmicutes | Bacillales | Geobacillus | Geobacillus sp. Y412MC52 | 0.04 |
| Firmicutes | Bacillales | Geobacillus | Geobacillus sp. Y412MC61 | 0.03 |
| Firmicutes | Bacillales | Geobacillus | Geobacillus stearothermophilus | 0.03 |
| Firmicutes | Bacillales | Geobacillus | Geobacillus thermodenitrificans | 0.08 |
| Firmicutes | Bacillales | Geobacillus | Geobacillus thermoglucosidasius | 0.02 |
| Firmicutes | Bacillales | Lysinibacillus | Lysinibacillus fusiformis | 0.02 |
| Firmicutes | Bacillales | Lysinibacillus | Lysinibacillus sphaericus | 0.04 |
| Firmicutes | Bacillales | Oceanobacillus | Oceanobacillus iheyensis | 0.07 |
| Firmicutes | Bacillales | Listeria | Listeria grayi | 0.02 |
| Firmicutes | Bacillales | Listeria | Listeria innocua | 0.05 |
| Firmicutes | Bacillales | Listeria | Listeria ivanovii | 0.01 |
| Firmicutes | Bacillales | Listeria | Listeria marthii | 0.01 |
| Firmicutes | Bacillales | Listeria | Listeria monocytogenes | 0.10 |
| Firmicutes | Bacillales | Listeria | Listeria seeligeri | 0.02 |
| Firmicutes | Bacillales | Listeria | Listeria welshimeri | 0.03 |
| Firmicutes | Bacillales | Brevibacillus | Brevibacillus brevis | 0.04 |
| Firmicutes | Bacillales | Paenibacillus | Paenibacillus curdlanolyticus | 0.01 |
| Firmicutes | Bacillales | Paenibacillus | Paenibacillus larvae | 0.03 |
| Firmicutes | Bacillales | Paenibacillus | Paenibacillus polymyxa | 0.07 |
| Firmicutes | Bacillales | Paenibacillus | Paenibacillus sp. | 0.09 |
| Firmicutes | Bacillales | Paenibacillus | Paenibacillus sp. JDR-2 | 0.07 |
| Firmicutes | Bacillales | Paenibacillus | Paenibacillus sp. Y412MC10 | 0.07 |
| Firmicutes | Bacillales | Paenibacillus | Paenibacillus sp. oral taxon 786 | 0.04 |
| Firmicutes | Bacillales | Paenibacillus | Paenibacillus vortex | 0.02 |
| Firmicutes | Bacillales | Planococcus | Planococcus donghaensis | 0.04 |
| Firmicutes | Bacillales | Macrococcus | Macrococcus caseolyticus | 0.04 |
| Firmicutes | Bacillales | Staphylococcus | Staphylococcus aureus | 0.15 |
| Firmicutes | Bacillales | Staphylococcus | Staphylococcus capitis | 0.01 |
| Firmicutes | Bacillales | Staphylococcus | Staphylococcus caprae | 0.01 |
| Firmicutes | Bacillales | Staphylococcus | Staphylococcus carnosus | 0.03 |
| Firmicutes | Bacillales | Staphylococcus | Staphylococcus epidermidis | 0.09 |
| Firmicutes | Bacillales | Staphylococcus | Staphylococcus haemolyticus | 0.07 |
| Firmicutes | Bacillales | Staphylococcus | Staphylococcus hominis | 0.03 |
| Firmicutes | Bacillales | Staphylococcus | Staphylococcus lugdunensis | 0.04 |
| Firmicutes | Bacillales | Staphylococcus | Staphylococcus pseudintermedius | 0.04 |
| Firmicutes | Bacillales | Staphylococcus | Staphylococcus saprophyticus | 0.05 |
| Firmicutes | Bacillales | Staphylococcus | Staphylococcus warneri | 0.02 |
| Firmicutes | Bacillales | Exiguobacterium | Exiguobacterium sibiricum | 0.04 |
| Firmicutes | Bacillales | Exiguobacterium | Exiguobacterium sp. | 0.01 |
| Firmicutes | Bacillales | Exiguobacterium | Exiguobacterium sp. AT1b | 0.01 |
| Firmicutes | Bacillales | Gemella | Gemella haemolysans | 0.01 |
| Firmicutes | Lactobacillales | Abiotrophia | Abiotrophia defectiva | 0.03 |
| Firmicutes | Lactobacillales | Aerococcus | Aerococcus viridans | 0.04 |
| Firmicutes | Lactobacillales | Eremococcus | Eremococcus coleocola | 0.02 |
| Firmicutes | Lactobacillales | Carnobacterium | Carnobacterium sp. | 0.05 |
| Firmicutes | Lactobacillales | Carnobacterium | Carnobacterium sp. AT7 | 0.06 |
| Firmicutes | Lactobacillales | Granulicatella | Granulicatella adiacens | 0.03 |
| Firmicutes | Lactobacillales | Granulicatella | Granulicatella elegans | 0.02 |
| Firmicutes | Lactobacillales | Enterococcus | Enterococcus casseliflavus | 0.05 |
| Firmicutes | Lactobacillales | Enterococcus | Enterococcus faecalis | 0.23 |
| Firmicutes | Lactobacillales | Enterococcus | Enterococcus faecium | 0.25 |
| Firmicutes | Lactobacillales | Enterococcus | Enterococcus gallinarum | 0.03 |
| Firmicutes | Lactobacillales | Enterococcus | Enterococcus italicus | 0.02 |
| Firmicutes | Lactobacillales | Enterococcus | Enterococcus sp. | 0.02 |
| Firmicutes | Lactobacillales | Enterococcus | Enterococcus sp. 7L76 | 0.01 |
| Firmicutes | Lactobacillales | Lactobacillus | Lactobacillus brevis | 0.03 |
| Firmicutes | Lactobacillales | Lactobacillus | Lactobacillus casei | 0.04 |
| Firmicutes | Lactobacillales | Lactobacillus | Lactobacillus delbrueckii | 0.03 |
| Firmicutes | Lactobacillales | Lactobacillus | Lactobacillus fermentum | 0.01 |
| Firmicutes | Lactobacillales | Lactobacillus | Lactobacillus gasseri | 0.01 |
| Firmicutes | Lactobacillales | Lactobacillus | Lactobacillus jensenii | 0.01 |
| Firmicutes | Lactobacillales | Lactobacillus | Lactobacillus johnsonii | 0.02 |
| Firmicutes | Lactobacillales | Lactobacillus | Lactobacillus plantarum | 0.05 |
| Firmicutes | Lactobacillales | Lactobacillus | Lactobacillus reuteri | 0.03 |
| Firmicutes | Lactobacillales | Lactobacillus | Lactobacillus rhamnosus | 0.02 |
| Firmicutes | Lactobacillales | Lactobacillus | Lactobacillus ruminis | 0.01 |
| Firmicutes | Lactobacillales | Lactobacillus | Lactobacillus sakei | 0.01 |
| Firmicutes | Lactobacillales | Lactobacillus | Lactobacillus salivarius | 0.04 |
| Firmicutes | Lactobacillales | Pediococcus | Pediococcus acidilactici | 0.01 |
| Firmicutes | Lactobacillales | Pediococcus | Pediococcus pentosaceus | 0.03 |
| Firmicutes | Lactobacillales | Leuconostoc | Leuconostoc gasicomitatum | 0.01 |
| Firmicutes | Lactobacillales | Leuconostoc | Leuconostoc kimchii | 0.01 |
| Firmicutes | Lactobacillales | Leuconostoc | Leuconostoc mesenteroides | 0.04 |
| Firmicutes | Lactobacillales | Oenococcus | Oenococcus oeni | 0.03 |
| Firmicutes | Lactobacillales | Lactococcus | Lactococcus lactis | 0.07 |
| Firmicutes | Lactobacillales | Streptococcus | Streptococcus agalactiae | 0.04 |
| Firmicutes | Lactobacillales | Streptococcus | Streptococcus dysgalactiae | 0.01 |
| Firmicutes | Lactobacillales | Streptococcus | Streptococcus equi | 0.01 |
| Firmicutes | Lactobacillales | Streptococcus | Streptococcus equinus | 0.01 |
| Firmicutes | Lactobacillales | Streptococcus | Streptococcus gallolyticus | 0.02 |
| Firmicutes | Lactobacillales | Streptococcus | Streptococcus gordonii | 0.03 |
| Firmicutes | Lactobacillales | Streptococcus | Streptococcus mitis | 0.02 |
| Firmicutes | Lactobacillales | Streptococcus | Streptococcus mutans | 0.02 |
| Firmicutes | Lactobacillales | Streptococcus | Streptococcus oralis | 0.01 |
| Firmicutes | Lactobacillales | Streptococcus | Streptococcus parasanguinis | 0.01 |
| Firmicutes | Lactobacillales | Streptococcus | Streptococcus pneumoniae | 0.08 |
| Firmicutes | Lactobacillales | Streptococcus | Streptococcus pyogenes | 0.04 |
| Firmicutes | Lactobacillales | Streptococcus | Streptococcus salivarius | 0.01 |
| Firmicutes | Lactobacillales | Streptococcus | Streptococcus sanguinis | 0.03 |
| Firmicutes | Lactobacillales | Streptococcus | Streptococcus sp. | 0.01 |
| Firmicutes | Lactobacillales | Streptococcus | Streptococcus sp. oral taxon 071 | 0.01 |
| Firmicutes | Lactobacillales | Streptococcus | Streptococcus suis | 0.07 |
| Firmicutes | Lactobacillales | Streptococcus | Streptococcus thermophilus | 0.04 |
| Firmicutes | Lactobacillales | Streptococcus | Streptococcus uberis | 0.02 |
| Firmicutes | Lactobacillales | Streptococcus | Streptococcus vestibularis | 0.01 |
| Firmicutes | Clostridiales | Alkaliphilus | Alkaliphilus metalliredigens | 0.12 |
| Firmicutes | Clostridiales | Alkaliphilus | Alkaliphilus oremlandii | 0.04 |
| Firmicutes | Clostridiales | Clostridium | Clostridium acetobutylicum | 0.08 |
| Firmicutes | Clostridiales | Clostridium | Clostridium asparagiforme | 0.23 |
| Firmicutes | Clostridiales | Clostridium | Clostridium bartlettii | 0.03 |
| Firmicutes | Clostridiales | Clostridium | Clostridium beijerinckii | 0.13 |
| Firmicutes | Clostridiales | Clostridium | Clostridium bolteae | 0.38 |
| Firmicutes | Clostridiales | Clostridium | Clostridium botulinum | 0.30 |
| Firmicutes | Clostridiales | Clostridium | Clostridium butyricum | 0.02 |
| Firmicutes | Clostridiales | Clostridium | Clostridium carboxidivorans | 0.03 |
| Firmicutes | Clostridiales | Clostridium | Clostridium cellulolyticum | 0.10 |
| Firmicutes | Clostridiales | Clostridium | Clostridium cellulovorans | 0.06 |
| Firmicutes | Clostridiales | Clostridium | Clostridium difficile | 0.22 |
| Firmicutes | Clostridiales | Clostridium | Clostridium hathewayi | 0.18 |
| Firmicutes | Clostridiales | Clostridium | Clostridium hiranonis | 0.01 |
| Firmicutes | Clostridiales | Clostridium | Clostridium hylemonae | 0.09 |
| Firmicutes | Clostridiales | Clostridium | Clostridium kluyveri | 0.07 |
| Firmicutes | Clostridiales | Clostridium | Clostridium leptum | 0.07 |
| Firmicutes | Clostridiales | Clostridium | Clostridium ljungdahlii | 0.04 |
| Firmicutes | Clostridiales | Clostridium | Clostridium methylpentosum | 0.04 |
| Firmicutes | Clostridiales | Clostridium | Clostridium nexile | 0.06 |
| Firmicutes | Clostridiales | Clostridium | Clostridium novyi | 0.07 |
| Firmicutes | Clostridiales | Clostridium | Clostridium papyrosolvens | 0.03 |
| Firmicutes | Clostridiales | Clostridium | Clostridium perfringens | 0.12 |
| Firmicutes | Clostridiales | Clostridium | Clostridium phytofermentans | 0.30 |
| Firmicutes | Clostridiales | Clostridium | Clostridium saccharolyticum | 0.41 |
| Firmicutes | Clostridiales | Clostridium | Clostridium scindens | 0.08 |
| Firmicutes | Clostridiales | Clostridium | Clostridium sp. | 0.04 |
| Firmicutes | Clostridiales | Clostridium | Clostridium sp. 7_2_43FAA | 0.01 |
| Firmicutes | Clostridiales | Clostridium | Clostridium sp. HGF2 | 0.04 |
| Firmicutes | Clostridiales | Clostridium | Clostridium sp. L2-50 | 0.03 |
| Firmicutes | Clostridiales | Clostridium | Clostridium sp. M62/1 | 0.08 |
| Firmicutes | Clostridiales | Clostridium | Clostridium sp. SS2/1 | 0.04 |
| Firmicutes | Clostridiales | Clostridium | Clostridium sporogenes | 0.01 |
| Firmicutes | Clostridiales | Clostridium | Clostridium sticklandii | 0.04 |
| Firmicutes | Clostridiales | Clostridium | Clostridium symbiosum | 0.11 |
| Firmicutes | Clostridiales | Clostridium | Clostridium tetani | 0.07 |
| Firmicutes | Clostridiales | Clostridium | Clostridium thermocellum | 0.14 |
| Firmicutes | Clostridiales | Anaerococcus | Anaerococcus hydrogenalis | 0.01 |
| Firmicutes | Clostridiales | Anaerococcus | Anaerococcus lactolyticus | 0.01 |
| Firmicutes | Clostridiales | Anaerococcus | Anaerococcus prevotii | 0.02 |
| Firmicutes | Clostridiales | Anaerococcus | Anaerococcus vaginalis | 0.01 |
| Firmicutes | Clostridiales | Finegoldia | Finegoldia magna | 0.04 |
| Firmicutes | Clostridiales | Peptoniphilus | Peptoniphilus duerdenii | 0.02 |
| Firmicutes | Clostridiales | Peptoniphilus | Peptoniphilus lacrimalis | 0.01 |
| Firmicutes | Clostridiales | Peptoniphilus | Peptoniphilus sp. oral taxon 386 | 0.01 |
| Firmicutes | Clostridiales | unclassified (derived from Clostridiales Family XI. Incertae Sedis) | Parvimonas micra | 0.01 |
| Firmicutes | Clostridiales | Thermaerobacter | Thermaerobacter marianensis | 0.02 |
| Firmicutes | Clostridiales | Thermaerobacter | Thermaerobacter subterraneus | 0.02 |
| Firmicutes | Clostridiales | Symbiobacterium | Symbiobacterium thermophilum | 0.06 |
| Firmicutes | Clostridiales | Anaerofustis | Anaerofustis stercorihominis | 0.05 |
| Firmicutes | Clostridiales | Eubacterium | Eubacterium cellulosolvens | 0.01 |
| Firmicutes | Clostridiales | Eubacterium | Eubacterium eligens | 0.13 |
| Firmicutes | Clostridiales | Eubacterium | Eubacterium hallii | 0.04 |
| Firmicutes | Clostridiales | Eubacterium | Eubacterium limosum | 0.12 |
| Firmicutes | Clostridiales | Eubacterium | Eubacterium rectale | 0.24 |
| Firmicutes | Clostridiales | Eubacterium | Eubacterium saburreum | 0.02 |
| Firmicutes | Clostridiales | Eubacterium | Eubacterium siraeum | 0.05 |
| Firmicutes | Clostridiales | Eubacterium | Eubacterium ventriosum | 0.04 |
| Firmicutes | Clostridiales | Eubacterium | Eubacterium yurii | 0.01 |
| Firmicutes | Clostridiales | Pseudoramibacter | Pseudoramibacter alactolyticus | 0.01 |
| Firmicutes | Clostridiales | Heliobacterium | Heliobacterium modesticaldum | 0.07 |
| Firmicutes | Clostridiales | Anaerostipes | Anaerostipes caccae | 0.06 |
| Firmicutes | Clostridiales | Anaerostipes | Anaerostipes sp. 3_2_56FAA | 0.02 |
| Firmicutes | Clostridiales | Butyrivibrio | Butyrivibrio crossotus | 0.04 |
| Firmicutes | Clostridiales | Butyrivibrio | Butyrivibrio fibrisolvens | 0.02 |
| Firmicutes | Clostridiales | Butyrivibrio | Clostridium proteoclasticum | 0.13 |
| Firmicutes | Clostridiales | Catonella | Catonella morbi | 0.03 |
| Firmicutes | Clostridiales | Cellulosilyticum | Clostridium lentocellum | 0.10 |
| Firmicutes | Clostridiales | Coprococcus | Coprococcus catus | 0.02 |
| Firmicutes | Clostridiales | Coprococcus | Coprococcus comes | 0.06 |
| Firmicutes | Clostridiales | Coprococcus | Coprococcus eutactus | 0.04 |
| Firmicutes | Clostridiales | Dorea | Dorea formicigenerans | 0.05 |
| Firmicutes | Clostridiales | Dorea | Dorea longicatena | 0.05 |
| Firmicutes | Clostridiales | Oribacterium | Oribacterium sinus | 0.04 |
| Firmicutes | Clostridiales | Oribacterium | Oribacterium sp. oral taxon 078 | 0.02 |
| Firmicutes | Clostridiales | Roseburia | Roseburia intestinalis | 0.06 |
| Firmicutes | Clostridiales | Roseburia | Roseburia inulinivorans | 0.08 |
| Firmicutes | Clostridiales | Shuttleworthia | Shuttleworthia satelles | 0.03 |
| Firmicutes | Clostridiales | unclassified (derived from Lachnospiraceae) | Lachnospiraceae bacterium 5_1_63FAA | 0.02 |
| Firmicutes | Clostridiales | unclassified (derived from Lachnospiraceae) | Lachnospiraceae bacterium 8_1_57FAA | 0.03 |
| Firmicutes | Clostridiales | unclassified (derived from Lachnospiraceae) | Marvinbryantia formatexigens | 0.07 |
| Firmicutes | Clostridiales | Candidatus Desulforudis | Candidatus Desulforudis audaxviator | 0.02 |
| Firmicutes | Clostridiales | Desulfitobacterium | Desulfitobacterium hafniense | 0.19 |
| Firmicutes | Clostridiales | Desulfotomaculum | Desulfotomaculum acetoxidans | 0.04 |
| Firmicutes | Clostridiales | Desulfotomaculum | Desulfotomaculum nigrificans | 0.01 |
| Firmicutes | Clostridiales | Desulfotomaculum | Desulfotomaculum reducens | 0.06 |
| Firmicutes | Clostridiales | Pelotomaculum | Pelotomaculum thermopropionicum | 0.06 |
| Firmicutes | Clostridiales | Thermincola | Thermincola potens | 0.03 |
| Firmicutes | Clostridiales | Filifactor | Filifactor alocis | 0.01 |
| Firmicutes | Clostridiales | Peptostreptococcus | Peptostreptococcus anaerobius | 0.02 |
| Firmicutes | Clostridiales | Peptostreptococcus | Peptostreptococcus stomatis | 0.01 |
| Firmicutes | Clostridiales | Acetivibrio | Acetivibrio cellulolyticus | 0.03 |
| Firmicutes | Clostridiales | Anaerotruncus | Anaerotruncus colihominis | 0.15 |
| Firmicutes | Clostridiales | Ethanoligenens | Ethanoligenens harbinense | 0.08 |
| Firmicutes | Clostridiales | Faecalibacterium | Faecalibacterium prausnitzii | 0.16 |
| Firmicutes | Clostridiales | Ruminococcus | Ruminococcus albus | 0.13 |
| Firmicutes | Clostridiales | Ruminococcus | Ruminococcus bromii | 0.03 |
| Firmicutes | Clostridiales | Ruminococcus | Ruminococcus flavefaciens | 0.03 |
| Firmicutes | Clostridiales | Ruminococcus | Ruminococcus gnavus | 0.06 |
| Firmicutes | Clostridiales | Ruminococcus | Ruminococcus lactaris | 0.06 |
| Firmicutes | Clostridiales | Ruminococcus | Ruminococcus obeum | 0.08 |
| Firmicutes | Clostridiales | Ruminococcus | Ruminococcus sp. | 0.06 |
| Firmicutes | Clostridiales | Ruminococcus | Ruminococcus sp. 18P13 | 0.02 |
| Firmicutes | Clostridiales | Ruminococcus | Ruminococcus sp. 5_1_39BFAA | 0.05 |
| Firmicutes | Clostridiales | Ruminococcus | Ruminococcus sp. SR1/5 | 0.02 |
| Firmicutes | Clostridiales | Ruminococcus | Ruminococcus torques | 0.08 |
| Firmicutes | Clostridiales | Subdoligranulum | Subdoligranulum variabile | 0.05 |
| Firmicutes | Clostridiales | unclassified (derived from Ruminococcaceae) | Ruminococcaceae bacterium D16 | 0.06 |
| Firmicutes | Clostridiales | Dethiobacter | Dethiobacter alkaliphilus | 0.01 |
| Firmicutes | Clostridiales | Syntrophomonas | Syntrophomonas wolfei | 0.04 |
| Firmicutes | Clostridiales | Syntrophothermus | Syntrophothermus lipocalidus | 0.01 |
| Firmicutes | Clostridiales | Blautia | Blautia hansenii | 0.07 |
| Firmicutes | Clostridiales | Blautia | Blautia hydrogenotrophica | 0.05 |
| Firmicutes | Clostridiales | Epulopiscium | Epulopiscium sp. | 0.02 |
| Firmicutes | Clostridiales | Epulopiscium | Epulopiscium sp. &#39;N.t. morphotype B&#39; | 0.02 |
| Firmicutes | Clostridiales | unclassified (derived from Clostridiales) | Clostridiales bacterium 1_7_47FAA | 0.14 |
| Firmicutes | Clostridiales | unclassified (derived from Clostridiales) | Clostridiales genomosp. BVAB3 | 0.01 |
| Firmicutes | Clostridiales | unclassified (derived from Clostridiales) | butyrate-producing bacterium SM4/1 | 0.01 |
| Firmicutes | Clostridiales | unclassified (derived from Clostridiales) | butyrate-producing bacterium SS3/4 | 0.04 |
| Firmicutes | Clostridiales | unclassified (derived from Clostridiales) | butyrate-producing bacterium SSC/2 | 0.02 |
| Firmicutes | Halanaerobiales | Halanaerobium | Halanaerobium praevalens | 0.01 |
| Firmicutes | Halanaerobiales | Halanaerobium | Halanaerobium sp. &#39;sapolanicus&#39; | 0.02 |
| Firmicutes | Halanaerobiales | Halothermothrix | Halothermothrix orenii | 0.04 |
| Firmicutes | Halanaerobiales | Acetohalobium | Acetohalobium arabaticum | 0.02 |
| Firmicutes | Natranaerobiales | Natranaerobius | Natranaerobius thermophilus | 0.03 |
| Firmicutes | Thermoanaerobacterales | Caldanaerobacter | Caldanaerobacter subterraneus | 0.09 |
| Firmicutes | Thermoanaerobacterales | Carboxydothermus | Carboxydothermus hydrogenoformans | 0.03 |
| Firmicutes | Thermoanaerobacterales | Moorella | Moorella thermoacetica | 0.06 |
| Firmicutes | Thermoanaerobacterales | Thermoanaerobacter | Thermoanaerobacter brockii | 0.03 |
| Firmicutes | Thermoanaerobacterales | Thermoanaerobacter | Thermoanaerobacter ethanolicus | 0.01 |
| Firmicutes | Thermoanaerobacterales | Thermoanaerobacter | Thermoanaerobacter italicus | 0.02 |
| Firmicutes | Thermoanaerobacterales | Thermoanaerobacter | Thermoanaerobacter mathranii | 0.01 |
| Firmicutes | Thermoanaerobacterales | Thermoanaerobacter | Thermoanaerobacter pseudethanolicus | 0.04 |
| Firmicutes | Thermoanaerobacterales | Thermoanaerobacter | Thermoanaerobacter sp. | 0.04 |
| Firmicutes | Thermoanaerobacterales | Thermoanaerobacter | Thermoanaerobacter sp. X513 | 0.03 |
| Firmicutes | Thermoanaerobacterales | Thermoanaerobacter | Thermoanaerobacter sp. X514 | 0.04 |
| Firmicutes | Thermoanaerobacterales | Thermoanaerobacter | Thermoanaerobacter sp. X561 | 0.03 |
| Firmicutes | Thermoanaerobacterales | Thermoanaerobacter | Thermoanaerobacter wiegelii | 0.01 |
| Firmicutes | Thermoanaerobacterales | Caldicellulosiruptor | Caldicellulosiruptor bescii | 0.02 |
| Firmicutes | Thermoanaerobacterales | Caldicellulosiruptor | Caldicellulosiruptor hydrothermalis | 0.01 |
| Firmicutes | Thermoanaerobacterales | Caldicellulosiruptor | Caldicellulosiruptor kristjanssonii | 0.01 |
| Firmicutes | Thermoanaerobacterales | Caldicellulosiruptor | Caldicellulosiruptor kronotskyensis | 0.01 |
| Firmicutes | Thermoanaerobacterales | Caldicellulosiruptor | Caldicellulosiruptor obsidiansis | 0.01 |
| Firmicutes | Thermoanaerobacterales | Caldicellulosiruptor | Caldicellulosiruptor saccharolyticus | 0.09 |
| Firmicutes | Thermoanaerobacterales | Thermoanaerobacterium | Thermoanaerobacterium thermosaccharolyticum | 0.03 |
| Firmicutes | Thermoanaerobacterales | Thermosediminibacter | Thermosediminibacter oceani | 0.04 |
| Firmicutes | Thermoanaerobacterales | Coprothermobacter | Coprothermobacter proteolyticus | 0.01 |
| Firmicutes | Erysipelotrichales | Bulleidia | Bulleidia extructa | 0.01 |
| Firmicutes | Erysipelotrichales | Catenibacterium | Catenibacterium mitsuokai | 0.03 |
| Firmicutes | Erysipelotrichales | Coprobacillus | Coprobacillus sp. 29_1 | 0.02 |
| Firmicutes | Erysipelotrichales | Coprobacillus | Coprobacillus sp. D7 | 0.04 |
| Firmicutes | Erysipelotrichales | Erysipelothrix | Erysipelothrix rhusiopathiae | 0.01 |
| Firmicutes | Erysipelotrichales | Holdemania | Holdemania filiformis | 0.17 |
| Firmicutes | Erysipelotrichales | Solobacterium | Solobacterium moorei | 0.01 |
| Firmicutes | Erysipelotrichales | Turicibacter | Turicibacter sanguinis | 0.02 |
| Firmicutes | Erysipelotrichales | unclassified (derived from Erysipelotrichaceae) | Clostridium ramosum | 0.04 |
| Firmicutes | Erysipelotrichales | unclassified (derived from Erysipelotrichaceae) | Clostridium spiroforme | 0.02 |
| Firmicutes | Erysipelotrichales | unclassified (derived from Erysipelotrichaceae) | Erysipelotrichaceae bacterium 3_1_53 | 0.01 |
| Firmicutes | Erysipelotrichales | unclassified (derived from Erysipelotrichaceae) | Erysipelotrichaceae bacterium 5_2_54FAA | 0.02 |
| Firmicutes | Erysipelotrichales | unclassified (derived from Erysipelotrichaceae) | Eubacterium biforme | 0.02 |
| Firmicutes | Erysipelotrichales | unclassified (derived from Erysipelotrichaceae) | Eubacterium cylindroides | 0.01 |
| Firmicutes | Erysipelotrichales | unclassified (derived from Erysipelotrichaceae) | Eubacterium dolichum | 0.02 |
| Firmicutes | Selenomonadales | Acidaminococcus | Acidaminococcus fermentans | 0.06 |
| Firmicutes | Selenomonadales | Acidaminococcus | Acidaminococcus sp. D21 | 0.02 |
| Firmicutes | Selenomonadales | Phascolarctobacterium | Phascolarctobacterium sp. YIT 12067 | 0.03 |
| Firmicutes | Selenomonadales | Dialister | Dialister invisus | 0.06 |
| Firmicutes | Selenomonadales | Dialister | Dialister micraerophilus | 0.02 |
| Firmicutes | Selenomonadales | Megamonas | Megamonas hypermegale | 0.07 |
| Firmicutes | Selenomonadales | Megasphaera | Megasphaera genomosp. type_1 | 0.02 |
| Firmicutes | Selenomonadales | Megasphaera | Megasphaera micronuciformis | 0.01 |
| Firmicutes | Selenomonadales | Mitsuokella | Mitsuokella multacida | 0.07 |
| Firmicutes | Selenomonadales | Selenomonas | Selenomonas artemidis | 0.01 |
| Firmicutes | Selenomonadales | Selenomonas | Selenomonas flueggei | 0.03 |
| Firmicutes | Selenomonadales | Selenomonas | Selenomonas noxia | 0.03 |
| Firmicutes | Selenomonadales | Selenomonas | Selenomonas sp. oral taxon 137 | 0.03 |
| Firmicutes | Selenomonadales | Selenomonas | Selenomonas sp. oral taxon 149 | 0.02 |
| Firmicutes | Selenomonadales | Selenomonas | Selenomonas sputigena | 0.06 |
| Firmicutes | Selenomonadales | Thermosinus | Thermosinus carboxydivorans | 0.03 |
| Firmicutes | Selenomonadales | Veillonella | Veillonella atypica | 0.03 |
| Firmicutes | Selenomonadales | Veillonella | Veillonella dispar | 0.01 |
| Firmicutes | Selenomonadales | Veillonella | Veillonella parvula | 0.11 |
| Firmicutes | Selenomonadales | Veillonella | Veillonella sp. 3_1_44 | 0.01 |
| Firmicutes | Selenomonadales | Veillonella | Veillonella sp. 6_1_27 | 0.01 |
| Firmicutes | Selenomonadales | Veillonella | Veillonella sp. oral taxon 158 | 0.01 |
| Fusobacteria | Fusobacteriales | Fusobacterium | Fusobacterium gonidiaformans | 0.01 |
| Fusobacteria | Fusobacteriales | Fusobacterium | Fusobacterium mortiferum | 0.02 |
| Fusobacteria | Fusobacteriales | Fusobacterium | Fusobacterium nucleatum | 0.11 |
| Fusobacteria | Fusobacteriales | Fusobacterium | Fusobacterium periodonticum | 0.01 |
| Fusobacteria | Fusobacteriales | Fusobacterium | Fusobacterium sp. | 0.02 |
| Fusobacteria | Fusobacteriales | Fusobacterium | Fusobacterium sp. 1_1_41FAA | 0.01 |
| Fusobacteria | Fusobacteriales | Fusobacterium | Fusobacterium sp. 7_1 | 0.01 |
| Fusobacteria | Fusobacteriales | Fusobacterium | Fusobacterium sp. D12 | 0.01 |
| Fusobacteria | Fusobacteriales | Fusobacterium | Fusobacterium ulcerans | 0.02 |
| Fusobacteria | Fusobacteriales | Fusobacterium | Fusobacterium varium | 0.04 |
| Fusobacteria | Fusobacteriales | Ilyobacter | Ilyobacter polytropus | 0.02 |
| Fusobacteria | Fusobacteriales | Leptotrichia | Leptotrichia buccalis | 0.02 |
| Fusobacteria | Fusobacteriales | Leptotrichia | Leptotrichia goodfellowii | 0.01 |
| Fusobacteria | Fusobacteriales | Leptotrichia | Leptotrichia hofstadii | 0.01 |
| Fusobacteria | Fusobacteriales | Sebaldella | Sebaldella termitidis | 0.06 |
| Fusobacteria | Fusobacteriales | Streptobacillus | Streptobacillus moniliformis | 0.02 |
| Gemmatimonadetes | Gemmatimonadales | Gemmatimonas | Gemmatimonas aurantiaca | 0.03 |
| Lentisphaerae | Lentisphaerales | Lentisphaera | Lentisphaera araneosa | 0.02 |
| Lentisphaerae | unclassified (derived from Lentisphaerae) | Victivallis | Victivallis vadensis | 0.05 |
| Nitrospirae | Nitrospirales | Leptospirillum | Leptospirillum sp. Group II &#39;5-way CG&#39; | 0.01 |
| Nitrospirae | Nitrospirales | Nitrospira | Candidatus Nitrospira defluvii | 0.01 |
| Planctomycetes | Planctomycetales | Blastopirellula | Blastopirellula marina | 0.03 |
| Planctomycetes | Planctomycetales | Gemmata | Gemmata obscuriglobus | 0.01 |
| Planctomycetes | Planctomycetales | Isosphaera | Isosphaera pallida | 0.02 |
| Planctomycetes | Planctomycetales | Pirellula | Pirellula staleyi | 0.02 |
| Planctomycetes | Planctomycetales | Planctomyces | Planctomyces brasiliensis | 0.01 |
| Planctomycetes | Planctomycetales | Planctomyces | Planctomyces limnophilus | 0.02 |
| Planctomycetes | Planctomycetales | Planctomyces | Planctomyces maris | 0.01 |
| Planctomycetes | Planctomycetales | Rhodopirellula | Rhodopirellula baltica | 0.07 |
| Planctomycetes | Planctomycetales | Candidatus Kuenenia | Candidatus Kuenenia stuttgartiensis | 0.01 |
| Proteobacteria | Caulobacterales | Asticcacaulis | Asticcacaulis excentricus | 0.05 |
| Proteobacteria | Caulobacterales | Brevundimonas | Brevundimonas sp. BAL3 | 0.01 |
| Proteobacteria | Caulobacterales | Brevundimonas | Brevundimonas subvibrioides | 0.03 |
| Proteobacteria | Caulobacterales | Caulobacter | Caulobacter segnis | 0.03 |
| Proteobacteria | Caulobacterales | Caulobacter | Caulobacter sp. | 0.02 |
| Proteobacteria | Caulobacterales | Caulobacter | Caulobacter sp. K31 | 0.03 |
| Proteobacteria | Caulobacterales | Caulobacter | Caulobacter vibrioides | 0.04 |
| Proteobacteria | Caulobacterales | Phenylobacterium | Phenylobacterium zucineum | 0.02 |
| Proteobacteria | Parvularculales | Parvularcula | Parvularcula bermudensis | 0.01 |
| Proteobacteria | Rhizobiales | Aurantimonas | Aurantimonas manganoxydans | 0.03 |
| Proteobacteria | Rhizobiales | Bartonella | Bartonella bacilliformis | 0.01 |
| Proteobacteria | Rhizobiales | Beijerinckia | Beijerinckia indica | 0.02 |
| Proteobacteria | Rhizobiales | Methylocella | Methylocella silvestris | 0.03 |
| Proteobacteria | Rhizobiales | Afipia | Afipia sp. 1NLS2 | 0.01 |
| Proteobacteria | Rhizobiales | Bradyrhizobium | Bradyrhizobium japonicum | 0.09 |
| Proteobacteria | Rhizobiales | Bradyrhizobium | Bradyrhizobium sp. | 0.08 |
| Proteobacteria | Rhizobiales | Bradyrhizobium | Bradyrhizobium sp. BTAi1 | 0.07 |
| Proteobacteria | Rhizobiales | Bradyrhizobium | Bradyrhizobium sp. ORS278 | 0.05 |
| Proteobacteria | Rhizobiales | Nitrobacter | Nitrobacter hamburgensis | 0.02 |
| Proteobacteria | Rhizobiales | Nitrobacter | Nitrobacter sp. Nb-311A | 0.01 |
| Proteobacteria | Rhizobiales | Nitrobacter | Nitrobacter winogradskyi | 0.03 |
| Proteobacteria | Rhizobiales | Oligotropha | Oligotropha carboxidovorans | 0.04 |
| Proteobacteria | Rhizobiales | Rhodopseudomonas | Rhodopseudomonas palustris | 0.23 |
| Proteobacteria | Rhizobiales | Brucella | Brucella abortus | 0.03 |
| Proteobacteria | Rhizobiales | Brucella | Brucella canis | 0.01 |
| Proteobacteria | Rhizobiales | Brucella | Brucella ceti | 0.02 |
| Proteobacteria | Rhizobiales | Brucella | Brucella melitensis | 0.04 |
| Proteobacteria | Rhizobiales | Brucella | Brucella microti | 0.01 |
| Proteobacteria | Rhizobiales | Brucella | Brucella neotomae | 0.01 |
| Proteobacteria | Rhizobiales | Brucella | Brucella ovis | 0.01 |
| Proteobacteria | Rhizobiales | Brucella | Brucella pinnipedialis | 0.01 |
| Proteobacteria | Rhizobiales | Brucella | Brucella sp. | 0.01 |
| Proteobacteria | Rhizobiales | Brucella | Brucella sp. 83/13 | 0.01 |
| Proteobacteria | Rhizobiales | Brucella | Brucella sp. F5/99 | 0.01 |
| Proteobacteria | Rhizobiales | Brucella | Brucella sp. NF 2653 | 0.01 |
| Proteobacteria | Rhizobiales | Brucella | Brucella sp. NVSL 07-0026 | 0.01 |
| Proteobacteria | Rhizobiales | Brucella | Brucella suis | 0.05 |
| Proteobacteria | Rhizobiales | Ochrobactrum | Ochrobactrum anthropi | 0.04 |
| Proteobacteria | Rhizobiales | Ochrobactrum | Ochrobactrum intermedium | 0.01 |
| Proteobacteria | Rhizobiales | Hyphomicrobium | Hyphomicrobium denitrificans | 0.01 |
| Proteobacteria | Rhizobiales | Rhodomicrobium | Rhodomicrobium vannielii | 0.02 |
| Proteobacteria | Rhizobiales | Methylobacterium | Methylobacterium chloromethanicum | 0.02 |
| Proteobacteria | Rhizobiales | Methylobacterium | Methylobacterium extorquens | 0.07 |
| Proteobacteria | Rhizobiales | Methylobacterium | Methylobacterium nodulans | 0.04 |
| Proteobacteria | Rhizobiales | Methylobacterium | Methylobacterium populi | 0.02 |
| Proteobacteria | Rhizobiales | Methylobacterium | Methylobacterium radiotolerans | 0.03 |
| Proteobacteria | Rhizobiales | Methylobacterium | Methylobacterium sp. | 0.03 |
| Proteobacteria | Rhizobiales | Methylobacterium | Methylobacterium sp. 4-46 | 0.04 |
| Proteobacteria | Rhizobiales | Methylocystis | Methylocystis sp. ATCC 49242 | 0.01 |
| Proteobacteria | Rhizobiales | Methylosinus | Methylosinus trichosporium | 0.02 |
| Proteobacteria | Rhizobiales | Chelativorans | Chelativorans sp. BNC1 | 0.04 |
| Proteobacteria | Rhizobiales | Hoeflea | Hoeflea phototrophica | 0.02 |
| Proteobacteria | Rhizobiales | Mesorhizobium | Mesorhizobium ciceri | 0.03 |
| Proteobacteria | Rhizobiales | Mesorhizobium | Mesorhizobium loti | 0.07 |
| Proteobacteria | Rhizobiales | Mesorhizobium | Mesorhizobium opportunistum | 0.02 |
| Proteobacteria | Rhizobiales | Parvibaculum | Parvibaculum lavamentivorans | 0.05 |
| Proteobacteria | Rhizobiales | Agrobacterium | Agrobacterium tumefaciens | 0.09 |
| Proteobacteria | Rhizobiales | Agrobacterium | Agrobacterium vitis | 0.03 |
| Proteobacteria | Rhizobiales | Rhizobium | Rhizobium etli | 0.07 |
| Proteobacteria | Rhizobiales | Rhizobium | Rhizobium leguminosarum | 0.11 |
| Proteobacteria | Rhizobiales | Sinorhizobium | Sinorhizobium fredii | 0.02 |
| Proteobacteria | Rhizobiales | Sinorhizobium | Sinorhizobium medicae | 0.04 |
| Proteobacteria | Rhizobiales | Sinorhizobium | Sinorhizobium meliloti | 0.08 |
| Proteobacteria | Rhizobiales | Azorhizobium | Azorhizobium caulinodans | 0.05 |
| Proteobacteria | Rhizobiales | Starkeya | Starkeya novella | 0.02 |
| Proteobacteria | Rhizobiales | Xanthobacter | Xanthobacter autotrophicus | 0.04 |
| Proteobacteria | Rhodobacterales | Hirschia | Hirschia baltica | 0.01 |
| Proteobacteria | Rhodobacterales | Hyphomonas | Hyphomonas neptunium | 0.05 |
| Proteobacteria | Rhodobacterales | Maricaulis | Maricaulis maris | 0.02 |
| Proteobacteria | Rhodobacterales | Oceanicaulis | Oceanicaulis sp. HTCC2633 | 0.01 |
| Proteobacteria | Rhodobacterales | Ahrensia | Ahrensia sp. R2A130 | 0.01 |
| Proteobacteria | Rhodobacterales | Citreicella | Citreicella sp. SE45 | 0.01 |
| Proteobacteria | Rhodobacterales | Dinoroseobacter | Dinoroseobacter shibae | 0.03 |
| Proteobacteria | Rhodobacterales | Jannaschia | Jannaschia sp. CCS1 | 0.02 |
| Proteobacteria | Rhodobacterales | Ketogulonicigenium | Ketogulonicigenium vulgare | 0.02 |
| Proteobacteria | Rhodobacterales | Labrenzia | Labrenzia aggregata | 0.02 |
| Proteobacteria | Rhodobacterales | Labrenzia | Labrenzia alexandrii | 0.02 |
| Proteobacteria | Rhodobacterales | Loktanella | Loktanella vestfoldensis | 0.01 |
| Proteobacteria | Rhodobacterales | Maritimibacter | Maritimibacter alkaliphilus | 0.03 |
| Proteobacteria | Rhodobacterales | Oceanibulbus | Oceanibulbus indolifex | 0.01 |
| Proteobacteria | Rhodobacterales | Oceanicola | Oceanicola batsensis | 0.03 |
| Proteobacteria | Rhodobacterales | Oceanicola | Oceanicola granulosus | 0.01 |
| Proteobacteria | Rhodobacterales | Octadecabacter | Octadecabacter antarcticus | 0.02 |
| Proteobacteria | Rhodobacterales | Paracoccus | Paracoccus denitrificans | 0.11 |
| Proteobacteria | Rhodobacterales | Pelagibaca | Pelagibaca bermudensis | 0.02 |
| Proteobacteria | Rhodobacterales | Phaeobacter | Phaeobacter gallaeciensis | 0.02 |
| Proteobacteria | Rhodobacterales | Pseudovibrio | Pseudovibrio sp. JE062 | 0.01 |
| Proteobacteria | Rhodobacterales | Rhodobacter | Rhodobacter capsulatus | 0.04 |
| Proteobacteria | Rhodobacterales | Rhodobacter | Rhodobacter sp. | 0.02 |
| Proteobacteria | Rhodobacterales | Rhodobacter | Rhodobacter sp. SW2 | 0.02 |
| Proteobacteria | Rhodobacterales | Rhodobacter | Rhodobacter sphaeroides | 0.13 |
| Proteobacteria | Rhodobacterales | Roseibium | Roseibium sp. TrichSKD4 | 0.01 |
| Proteobacteria | Rhodobacterales | Roseobacter | Roseobacter denitrificans | 0.03 |
| Proteobacteria | Rhodobacterales | Roseobacter | Roseobacter litoralis | 0.01 |
| Proteobacteria | Rhodobacterales | Roseobacter | Roseobacter sp. GAI101 | 0.01 |
| Proteobacteria | Rhodobacterales | Roseobacter | Roseobacter sp. MED193 | 0.02 |
| Proteobacteria | Rhodobacterales | Roseobacter | Roseobacter sp. SK209-2-6 | 0.01 |
| Proteobacteria | Rhodobacterales | Roseovarius | Roseovarius nubinhibens | 0.01 |
| Proteobacteria | Rhodobacterales | Roseovarius | Roseovarius sp. 217 | 0.01 |
| Proteobacteria | Rhodobacterales | Roseovarius | Roseovarius sp. TM1035 | 0.01 |
| Proteobacteria | Rhodobacterales | Ruegeria | Ruegeria lacuscaerulensis | 0.01 |
| Proteobacteria | Rhodobacterales | Ruegeria | Ruegeria pomeroyi | 0.04 |
| Proteobacteria | Rhodobacterales | Ruegeria | Ruegeria sp. TM1040 | 0.03 |
| Proteobacteria | Rhodobacterales | Ruegeria | Silicibacter sp. TrichCH4B | 0.01 |
| Proteobacteria | Rhodobacterales | Sagittula | Sagittula stellata | 0.01 |
| Proteobacteria | Rhodobacterales | Sulfitobacter | Sulfitobacter sp. EE-36 | 0.01 |
| Proteobacteria | Rhodobacterales | Sulfitobacter | Sulfitobacter sp. NAS-14.1 | 0.02 |
| Proteobacteria | Rhodobacterales | Thalassobium | Thalassiobium sp. R2A62 | 0.01 |
| Proteobacteria | Rhodobacterales | unclassified (derived from Rhodobacteraceae) | Rhodobacteraceae bacterium HTCC2150 | 0.01 |
| Proteobacteria | Rhodobacterales | unclassified (derived from Rhodobacterales) | Rhodobacterales bacterium HTCC2255 | 0.01 |
| Proteobacteria | Rhodobacterales | unclassified (derived from Rhodobacterales) | Rhodobacterales bacterium Y4I | 0.01 |
| Proteobacteria | Rhodospirillales | Acetobacter | Acetobacter pasteurianus | 0.02 |
| Proteobacteria | Rhodospirillales | Acidiphilium | Acidiphilium cryptum | 0.03 |
| Proteobacteria | Rhodospirillales | Gluconacetobacter | Gluconacetobacter hansenii | 0.01 |
| Proteobacteria | Rhodospirillales | Gluconobacter | Gluconobacter oxydans | 0.02 |
| Proteobacteria | Rhodospirillales | Granulibacter | Granulibacter bethesdensis | 0.03 |
| Proteobacteria | Rhodospirillales | Roseomonas | Roseomonas cervicalis | 0.02 |
| Proteobacteria | Rhodospirillales | Azospirillum | Azospirillum sp. | 0.03 |
| Proteobacteria | Rhodospirillales | Azospirillum | Azospirillum sp. B510 | 0.03 |
| Proteobacteria | Rhodospirillales | Magnetospirillum | Magnetospirillum gryphiswaldense | 0.03 |
| Proteobacteria | Rhodospirillales | Magnetospirillum | Magnetospirillum magneticum | 0.06 |
| Proteobacteria | Rhodospirillales | Magnetospirillum | Magnetospirillum magnetotacticum | 0.05 |
| Proteobacteria | Rhodospirillales | Rhodospirillum | Rhodospirillum centenum | 0.03 |
| Proteobacteria | Rhodospirillales | Rhodospirillum | Rhodospirillum rubrum | 0.07 |
| Proteobacteria | Rickettsiales | Rickettsia | Rickettsia prowazekii | 0.02 |
| Proteobacteria | Sphingomonadales | Erythrobacter | Erythrobacter litoralis | 0.02 |
| Proteobacteria | Sphingomonadales | Erythrobacter | Erythrobacter sp. | 0.01 |
| Proteobacteria | Sphingomonadales | Erythrobacter | Erythrobacter sp. NAP1 | 0.01 |
| Proteobacteria | Sphingomonadales | Erythrobacter | Erythrobacter sp. SD-21 | 0.01 |
| Proteobacteria | Sphingomonadales | Novosphingobium | Novosphingobium aromaticivorans | 0.05 |
| Proteobacteria | Sphingomonadales | Sphingobium | Sphingobium chlorophenolicum | 0.02 |
| Proteobacteria | Sphingomonadales | Sphingobium | Sphingobium japonicum | 0.02 |
| Proteobacteria | Sphingomonadales | Sphingomonas | Sphingomonas sp. | 0.02 |
| Proteobacteria | Sphingomonadales | Sphingomonas | Sphingomonas sp. SKA58 | 0.02 |
| Proteobacteria | Sphingomonadales | Sphingomonas | Sphingomonas wittichii | 0.08 |
| Proteobacteria | Sphingomonadales | Sphingopyxis | Sphingopyxis alaskensis | 0.03 |
| Proteobacteria | Sphingomonadales | Zymomonas | Zymomonas mobilis | 0.03 |
| Proteobacteria | unclassified (derived from Alphaproteobacteria) | Candidatus Puniceispirillum | Candidatus Puniceispirillum marinum | 0.01 |
| Proteobacteria | unclassified (derived from Alphaproteobacteria) | unclassified (derived from Alphaproteobacteria) | alpha proteobacterium BAL199 | 0.01 |
| Proteobacteria | Burkholderiales | Achromobacter | Achromobacter piechaudii | 0.10 |
| Proteobacteria | Burkholderiales | Achromobacter | Achromobacter xylosoxidans | 0.19 |
| Proteobacteria | Burkholderiales | Bordetella | Bordetella avium | 0.10 |
| Proteobacteria | Burkholderiales | Bordetella | Bordetella bronchiseptica | 0.14 |
| Proteobacteria | Burkholderiales | Bordetella | Bordetella parapertussis | 0.10 |
| Proteobacteria | Burkholderiales | Bordetella | Bordetella pertussis | 0.09 |
| Proteobacteria | Burkholderiales | Bordetella | Bordetella petrii | 0.12 |
| Proteobacteria | Burkholderiales | Sutterella | Sutterella wadsworthensis | 0.03 |
| Proteobacteria | Burkholderiales | Taylorella | Taylorella equigenitalis | 0.01 |
| Proteobacteria | Burkholderiales | Burkholderia | Burkholderia ambifaria | 0.39 |
| Proteobacteria | Burkholderiales | Burkholderia | Burkholderia cenocepacia | 0.54 |
| Proteobacteria | Burkholderiales | Burkholderia | Burkholderia cepacia | 0.05 |
| Proteobacteria | Burkholderiales | Burkholderia | Burkholderia dolosa | 0.11 |
| Proteobacteria | Burkholderiales | Burkholderia | Burkholderia glumae | 0.08 |
| Proteobacteria | Burkholderiales | Burkholderia | Burkholderia graminis | 0.05 |
| Proteobacteria | Burkholderiales | Burkholderia | Burkholderia mallei | 0.07 |
| Proteobacteria | Burkholderiales | Burkholderia | Burkholderia multivorans | 0.24 |
| Proteobacteria | Burkholderiales | Burkholderia | Burkholderia oklahomensis | 0.06 |
| Proteobacteria | Burkholderiales | Burkholderia | Burkholderia phymatum | 0.10 |
| Proteobacteria | Burkholderiales | Burkholderia | Burkholderia phytofirmans | 0.04 |
| Proteobacteria | Burkholderiales | Burkholderia | Burkholderia pseudomallei | 0.27 |
| Proteobacteria | Burkholderiales | Burkholderia | Burkholderia rhizoxinica | 0.02 |
| Proteobacteria | Burkholderiales | Burkholderia | Burkholderia sp. | 0.34 |
| Proteobacteria | Burkholderiales | Burkholderia | Burkholderia sp. 383 | 0.23 |
| Proteobacteria | Burkholderiales | Burkholderia | Burkholderia sp. CCGE1001 | 0.02 |
| Proteobacteria | Burkholderiales | Burkholderia | Burkholderia sp. CCGE1002 | 0.05 |
| Proteobacteria | Burkholderiales | Burkholderia | Burkholderia sp. CCGE1003 | 0.03 |
| Proteobacteria | Burkholderiales | Burkholderia | Burkholderia sp. Ch1-1 | 0.04 |
| Proteobacteria | Burkholderiales | Burkholderia | Burkholderia sp. H160 | 0.03 |
| Proteobacteria | Burkholderiales | Burkholderia | Burkholderia thailandensis | 0.13 |
| Proteobacteria | Burkholderiales | Burkholderia | Burkholderia ubonensis | 0.05 |
| Proteobacteria | Burkholderiales | Burkholderia | Burkholderia vietnamiensis | 0.14 |
| Proteobacteria | Burkholderiales | Burkholderia | Burkholderia xenovorans | 0.12 |
| Proteobacteria | Burkholderiales | Cupriavidus | Cupriavidus metallidurans | 0.12 |
| Proteobacteria | Burkholderiales | Cupriavidus | Cupriavidus necator | 0.07 |
| Proteobacteria | Burkholderiales | Cupriavidus | Cupriavidus pinatubonensis | 0.08 |
| Proteobacteria | Burkholderiales | Cupriavidus | Cupriavidus taiwanensis | 0.07 |
| Proteobacteria | Burkholderiales | Lautropia | Lautropia mirabilis | 0.01 |
| Proteobacteria | Burkholderiales | Limnobacter | Limnobacter sp. MED105 | 0.03 |
| Proteobacteria | Burkholderiales | Polynucleobacter | Polynucleobacter necessarius | 0.02 |
| Proteobacteria | Burkholderiales | Ralstonia | Ralstonia pickettii | 0.06 |
| Proteobacteria | Burkholderiales | Ralstonia | Ralstonia solanacearum | 0.19 |
| Proteobacteria | Burkholderiales | Ralstonia | Ralstonia sp. | 0.02 |
| Proteobacteria | Burkholderiales | Ralstonia | Ralstonia sp. 5_7_47FAA | 0.02 |
| Proteobacteria | Burkholderiales | Acidovorax | Acidovorax avenae | 0.08 |
| Proteobacteria | Burkholderiales | Acidovorax | Acidovorax citrulli | 0.12 |
| Proteobacteria | Burkholderiales | Acidovorax | Acidovorax delafieldii | 0.04 |
| Proteobacteria | Burkholderiales | Acidovorax | Acidovorax ebreus | 0.02 |
| Proteobacteria | Burkholderiales | Acidovorax | Acidovorax sp. JS42 | 0.10 |
| Proteobacteria | Burkholderiales | Albidiferax | Albidiferax ferrireducens | 0.07 |
| Proteobacteria | Burkholderiales | Alicycliphilus | Alicycliphilus denitrificans | 0.06 |
| Proteobacteria | Burkholderiales | Comamonas | Comamonas testosteroni | 0.16 |
| Proteobacteria | Burkholderiales | Curvibacter | Curvibacter putative symbiont of Hydra magnipapillata | 0.02 |
| Proteobacteria | Burkholderiales | Delftia | Delftia acidovorans | 0.13 |
| Proteobacteria | Burkholderiales | Polaromonas | Polaromonas naphthalenivorans | 0.06 |
| Proteobacteria | Burkholderiales | Polaromonas | Polaromonas sp. JS666 | 0.07 |
| Proteobacteria | Burkholderiales | Variovorax | Variovorax paradoxus | 0.16 |
| Proteobacteria | Burkholderiales | Verminephrobacter | Verminephrobacter eiseniae | 0.12 |
| Proteobacteria | Burkholderiales | Herbaspirillum | Herbaspirillum seropedicae | 0.05 |
| Proteobacteria | Burkholderiales | Herminiimonas | Herminiimonas arsenicoxydans | 0.06 |
| Proteobacteria | Burkholderiales | Janthinobacterium | Janthinobacterium sp. Marseille | 0.11 |
| Proteobacteria | Burkholderiales | Oxalobacter | Oxalobacter formigenes | 0.04 |
| Proteobacteria | Burkholderiales | Leptothrix | Leptothrix cholodnii | 0.04 |
| Proteobacteria | Burkholderiales | Methylibium | Methylibium petroleiphilum | 0.07 |
| Proteobacteria | Burkholderiales | Thiomonas | Thiomonas intermedia | 0.02 |
| Proteobacteria | Burkholderiales | unclassified (derived from Burkholderiales) | Burkholderiales bacterium 1_1_47 | 0.02 |
| Proteobacteria | Gallionellales | Gallionella | Gallionella capsiferriformans | 0.01 |
| Proteobacteria | Gallionellales | Sideroxydans | Sideroxydans lithotrophicus | 0.02 |
| Proteobacteria | Hydrogenophilales | Thiobacillus | Thiobacillus denitrificans | 0.06 |
| Proteobacteria | Methylophilales | Methylobacillus | Methylobacillus flagellatus | 0.08 |
| Proteobacteria | Methylophilales | Methylotenera | Methylotenera mobilis | 0.02 |
| Proteobacteria | Methylophilales | Methylotenera | Methylotenera sp. 301 | 0.02 |
| Proteobacteria | Methylophilales | Methylovorus | Methylovorus sp. MP688 | 0.01 |
| Proteobacteria | Methylophilales | Methylovorus | Methylovorus sp. SIP3-4 | 0.01 |
| Proteobacteria | Neisseriales | Chromobacterium | Chromobacterium violaceum | 0.11 |
| Proteobacteria | Neisseriales | Eikenella | Eikenella corrodens | 0.01 |
| Proteobacteria | Neisseriales | Kingella | Kingella oralis | 0.01 |
| Proteobacteria | Neisseriales | Laribacter | Laribacter hongkongensis | 0.04 |
| Proteobacteria | Neisseriales | Lutiella | Lutiella nitroferrum | 0.05 |
| Proteobacteria | Neisseriales | Neisseria | Neisseria elongata | 0.01 |
| Proteobacteria | Neisseriales | Neisseria | Neisseria flavescens | 0.01 |
| Proteobacteria | Neisseriales | Neisseria | Neisseria gonorrhoeae | 0.01 |
| Proteobacteria | Neisseriales | Neisseria | Neisseria lactamica | 0.01 |
| Proteobacteria | Neisseriales | Neisseria | Neisseria meningitidis | 0.06 |
| Proteobacteria | Neisseriales | Neisseria | Neisseria mucosa | 0.01 |
| Proteobacteria | Neisseriales | Neisseria | Neisseria sicca | 0.01 |
| Proteobacteria | Neisseriales | Neisseria | Neisseria sp. | 0.01 |
| Proteobacteria | Neisseriales | Neisseria | Neisseria sp. oral taxon 014 | 0.01 |
| Proteobacteria | Neisseriales | Neisseria | Neisseria subflava | 0.01 |
| Proteobacteria | Nitrosomonadales | Nitrosomonas | Nitrosomonas europaea | 0.05 |
| Proteobacteria | Nitrosomonadales | Nitrosomonas | Nitrosomonas eutropha | 0.05 |
| Proteobacteria | Nitrosomonadales | Nitrosomonas | Nitrosomonas sp. | 0.01 |
| Proteobacteria | Nitrosomonadales | Nitrosomonas | Nitrosomonas sp. AL212 | 0.01 |
| Proteobacteria | Nitrosomonadales | Nitrosospira | Nitrosospira multiformis | 0.05 |
| Proteobacteria | Rhodocyclales | Aromatoleum | Aromatoleum aromaticum | 0.09 |
| Proteobacteria | Rhodocyclales | Azoarcus | Azoarcus sp. | 0.05 |
| Proteobacteria | Rhodocyclales | Azoarcus | Azoarcus sp. BH72 | 0.09 |
| Proteobacteria | Rhodocyclales | Dechloromonas | Dechloromonas aromatica | 0.08 |
| Proteobacteria | Rhodocyclales | Thauera | Thauera sp. MZ1T | 0.05 |
| Proteobacteria | unclassified (derived from Betaproteobacteria) | Candidatus Accumulibacter | Candidatus Accumulibacter phosphatis | 0.03 |
| Proteobacteria | Bdellovibrionales | Bdellovibrio | Bdellovibrio bacteriovorus | 0.02 |
| Proteobacteria | Desulfarculales | Desulfarculus | Desulfarculus baarsii | 0.02 |
| Proteobacteria | Desulfobacterales | Desulfatibacillum | Desulfatibacillum alkenivorans | 0.02 |
| Proteobacteria | Desulfobacterales | Desulfobacterium | Desulfobacterium autotrophicum | 0.03 |
| Proteobacteria | Desulfobacterales | Desulfococcus | Desulfococcus oleovorans | 0.01 |
| Proteobacteria | Desulfobacterales | Desulfobulbus | Desulfobulbus propionicus | 0.01 |
| Proteobacteria | Desulfobacterales | Desulfotalea | Desulfotalea psychrophila | 0.03 |
| Proteobacteria | Desulfobacterales | Desulfurivibrio | Desulfurivibrio alkaliphilus | 0.02 |
| Proteobacteria | Desulfovibrionales | Desulfohalobium | Desulfohalobium retbaense | 0.02 |
| Proteobacteria | Desulfovibrionales | Desulfonatronospira | Desulfonatronospira thiodismutans | 0.01 |
| Proteobacteria | Desulfovibrionales | Desulfomicrobium | Desulfomicrobium baculatum | 0.04 |
| Proteobacteria | Desulfovibrionales | Bilophila | Bilophila wadsworthia | 0.05 |
| Proteobacteria | Desulfovibrionales | Desulfovibrio | Desulfovibrio aespoeensis | 0.01 |
| Proteobacteria | Desulfovibrionales | Desulfovibrio | Desulfovibrio desulfuricans | 0.12 |
| Proteobacteria | Desulfovibrionales | Desulfovibrio | Desulfovibrio fructosovorans | 0.01 |
| Proteobacteria | Desulfovibrionales | Desulfovibrio | Desulfovibrio magneticus | 0.01 |
| Proteobacteria | Desulfovibrionales | Desulfovibrio | Desulfovibrio piger | 0.02 |
| Proteobacteria | Desulfovibrionales | Desulfovibrio | Desulfovibrio salexigens | 0.02 |
| Proteobacteria | Desulfovibrionales | Desulfovibrio | Desulfovibrio sp. | 0.03 |
| Proteobacteria | Desulfovibrionales | Desulfovibrio | Desulfovibrio sp. 3_1_syn3 | 0.02 |
| Proteobacteria | Desulfovibrionales | Desulfovibrio | Desulfovibrio sp. FW1012B | 0.01 |
| Proteobacteria | Desulfovibrionales | Desulfovibrio | Desulfovibrio sp. ND132 | 0.01 |
| Proteobacteria | Desulfovibrionales | Desulfovibrio | Desulfovibrio vulgaris | 0.09 |
| Proteobacteria | Desulfovibrionales | Lawsonia | Lawsonia intracellularis | 0.01 |
| Proteobacteria | Desulfuromonadales | Desulfuromonas | Desulfuromonas acetoxidans | 0.03 |
| Proteobacteria | Desulfuromonadales | Geobacter | Geobacter bemidjiensis | 0.04 |
| Proteobacteria | Desulfuromonadales | Geobacter | Geobacter lovleyi | 0.03 |
| Proteobacteria | Desulfuromonadales | Geobacter | Geobacter metallireducens | 0.05 |
| Proteobacteria | Desulfuromonadales | Geobacter | Geobacter sp. | 0.03 |
| Proteobacteria | Desulfuromonadales | Geobacter | Geobacter sp. FRC-32 | 0.02 |
| Proteobacteria | Desulfuromonadales | Geobacter | Geobacter sp. M18 | 0.01 |
| Proteobacteria | Desulfuromonadales | Geobacter | Geobacter sulfurreducens | 0.04 |
| Proteobacteria | Desulfuromonadales | Geobacter | Geobacter uraniireducens | 0.05 |
| Proteobacteria | Desulfuromonadales | Pelobacter | Pelobacter carbinolicus | 0.05 |
| Proteobacteria | Desulfuromonadales | Pelobacter | Pelobacter propionicus | 0.05 |
| Proteobacteria | Myxococcales | Stigmatella | Stigmatella aurantiaca | 0.04 |
| Proteobacteria | Myxococcales | Haliangium | Haliangium ochraceum | 0.04 |
| Proteobacteria | Myxococcales | Anaeromyxobacter | Anaeromyxobacter dehalogenans | 0.05 |
| Proteobacteria | Myxococcales | Anaeromyxobacter | Anaeromyxobacter sp. Fw109-5 | 0.05 |
| Proteobacteria | Myxococcales | Anaeromyxobacter | Anaeromyxobacter sp. K | 0.01 |
| Proteobacteria | Myxococcales | Myxococcus | Myxococcus xanthus | 0.08 |
| Proteobacteria | Myxococcales | Sorangium | Sorangium cellulosum | 0.07 |
| Proteobacteria | Syntrophobacterales | Syntrophus | Syntrophus aciditrophicus | 0.03 |
| Proteobacteria | Syntrophobacterales | Syntrophobacter | Syntrophobacter fumaroxidans | 0.05 |
| Proteobacteria | unclassified (derived from Deltaproteobacteria) | unclassified (derived from Deltaproteobacteria) | delta proteobacterium MLMS-1 | 0.02 |
| Proteobacteria | unclassified (derived from Deltaproteobacteria) | unclassified (derived from Deltaproteobacteria) | delta proteobacterium NaphS2 | 0.01 |
| Proteobacteria | Campylobacterales | Arcobacter | Arcobacter butzleri | 0.01 |
| Proteobacteria | Campylobacterales | Campylobacter | Campylobacter concisus | 0.01 |
| Proteobacteria | Campylobacterales | Campylobacter | Campylobacter curvus | 0.01 |
| Proteobacteria | Campylobacterales | Campylobacter | Campylobacter fetus | 0.01 |
| Proteobacteria | Campylobacterales | Campylobacter | Campylobacter hominis | 0.01 |
| Proteobacteria | Campylobacterales | Campylobacter | Campylobacter jejuni | 0.04 |
| Proteobacteria | Campylobacterales | Campylobacter | Campylobacter lari | 0.01 |
| Proteobacteria | Campylobacterales | Campylobacter | Campylobacter upsaliensis | 0.01 |
| Proteobacteria | Campylobacterales | Sulfurospirillum | Sulfurospirillum deleyianum | 0.01 |
| Proteobacteria | Campylobacterales | Helicobacter | Helicobacter cinaedi | 0.01 |
| Proteobacteria | Campylobacterales | Helicobacter | Helicobacter felis | 0.01 |
| Proteobacteria | Campylobacterales | Helicobacter | Helicobacter hepaticus | 0.02 |
| Proteobacteria | Campylobacterales | Helicobacter | Helicobacter mustelae | 0.01 |
| Proteobacteria | Campylobacterales | Helicobacter | Helicobacter pullorum | 0.01 |
| Proteobacteria | Campylobacterales | Helicobacter | Helicobacter pylori | 0.04 |
| Proteobacteria | Campylobacterales | Sulfurimonas | Sulfurimonas denitrificans | 0.01 |
| Proteobacteria | Campylobacterales | Wolinella | Wolinella succinogenes | 0.02 |
| Proteobacteria | unclassified (derived from Epsilonproteobacteria) | Nitratiruptor | Nitratiruptor sp. SB155-2 | 0.02 |
| Proteobacteria | unclassified (derived from Epsilonproteobacteria) | Sulfurovum | Sulfurovum sp. NBC37-1 | 0.01 |
| Proteobacteria | Acidithiobacillales | Acidithiobacillus | Acidithiobacillus caldus | 0.01 |
| Proteobacteria | Acidithiobacillales | Acidithiobacillus | Acidithiobacillus ferrooxidans | 0.02 |
| Proteobacteria | Aeromonadales | Aeromonas | Aeromonas hydrophila | 0.07 |
| Proteobacteria | Aeromonadales | Aeromonas | Aeromonas salmonicida | 0.05 |
| Proteobacteria | Aeromonadales | Tolumonas | Tolumonas auensis | 0.03 |
| Proteobacteria | Aeromonadales | Succinatimonas | Succinatimonas hippei | 0.01 |
| Proteobacteria | Alteromonadales | Alteromonas | Alteromonas macleodii | 0.07 |
| Proteobacteria | Alteromonadales | Glaciecola | Glaciecola sp. HTCC2999 | 0.01 |
| Proteobacteria | Alteromonadales | Marinobacter | Marinobacter algicola | 0.08 |
| Proteobacteria | Alteromonadales | Marinobacter | Marinobacter hydrocarbonoclasticus | 0.17 |
| Proteobacteria | Alteromonadales | Marinobacter | Marinobacter sp. | 0.05 |
| Proteobacteria | Alteromonadales | Marinobacter | Marinobacter sp. ELB17 | 0.07 |
| Proteobacteria | Alteromonadales | Marinobacter | marine bacterium HP15 | 0.05 |
| Proteobacteria | Alteromonadales | Saccharophagus | Saccharophagus degradans | 0.08 |
| Proteobacteria | Alteromonadales | Colwellia | Colwellia psychrerythraea | 0.05 |
| Proteobacteria | Alteromonadales | Ferrimonas | Ferrimonas balearica | 0.02 |
| Proteobacteria | Alteromonadales | Idiomarina | Idiomarina baltica | 0.03 |
| Proteobacteria | Alteromonadales | Idiomarina | Idiomarina loihiensis | 0.06 |
| Proteobacteria | Alteromonadales | Moritella | Moritella sp. | 0.02 |
| Proteobacteria | Alteromonadales | Moritella | Moritella sp. PE36 | 0.02 |
| Proteobacteria | Alteromonadales | Pseudoalteromonas | Pseudoalteromonas atlantica | 0.05 |
| Proteobacteria | Alteromonadales | Pseudoalteromonas | Pseudoalteromonas haloplanktis | 0.06 |
| Proteobacteria | Alteromonadales | Pseudoalteromonas | Pseudoalteromonas sp. | 0.01 |
| Proteobacteria | Alteromonadales | Pseudoalteromonas | Pseudoalteromonas sp. SM9913 | 0.02 |
| Proteobacteria | Alteromonadales | Pseudoalteromonas | Pseudoalteromonas tunicata | 0.02 |
| Proteobacteria | Alteromonadales | Psychromonas | Psychromonas ingrahamii | 0.03 |
| Proteobacteria | Alteromonadales | Psychromonas | Psychromonas sp. CNPT3 | 0.01 |
| Proteobacteria | Alteromonadales | Shewanella | Shewanella amazonensis | 0.04 |
| Proteobacteria | Alteromonadales | Shewanella | Shewanella baltica | 0.06 |
| Proteobacteria | Alteromonadales | Shewanella | Shewanella benthica | 0.01 |
| Proteobacteria | Alteromonadales | Shewanella | Shewanella denitrificans | 0.04 |
| Proteobacteria | Alteromonadales | Shewanella | Shewanella frigidimarina | 0.02 |
| Proteobacteria | Alteromonadales | Shewanella | Shewanella halifaxensis | 0.03 |
| Proteobacteria | Alteromonadales | Shewanella | Shewanella loihica | 0.03 |
| Proteobacteria | Alteromonadales | Shewanella | Shewanella oneidensis | 0.04 |
| Proteobacteria | Alteromonadales | Shewanella | Shewanella pealeana | 0.08 |
| Proteobacteria | Alteromonadales | Shewanella | Shewanella piezotolerans | 0.04 |
| Proteobacteria | Alteromonadales | Shewanella | Shewanella putrefaciens | 0.04 |
| Proteobacteria | Alteromonadales | Shewanella | Shewanella sediminis | 0.05 |
| Proteobacteria | Alteromonadales | Shewanella | Shewanella sp. | 0.06 |
| Proteobacteria | Alteromonadales | Shewanella | Shewanella sp. ANA-3 | 0.03 |
| Proteobacteria | Alteromonadales | Shewanella | Shewanella sp. MR-4 | 0.02 |
| Proteobacteria | Alteromonadales | Shewanella | Shewanella sp. MR-7 | 0.01 |
| Proteobacteria | Alteromonadales | Shewanella | Shewanella sp. W3-18-1 | 0.04 |
| Proteobacteria | Alteromonadales | Shewanella | Shewanella violacea | 0.01 |
| Proteobacteria | Alteromonadales | Shewanella | Shewanella woodyi | 0.03 |
| Proteobacteria | Alteromonadales | Teredinibacter | Teredinibacter turnerae | 0.05 |
| Proteobacteria | Alteromonadales | unclassified (derived from Alteromonadales) | Alteromonadales bacterium TW-7 | 0.01 |
| Proteobacteria | Cardiobacteriales | Cardiobacterium | Cardiobacterium hominis | 0.01 |
| Proteobacteria | Cardiobacteriales | Dichelobacter | Dichelobacter nodosus | 0.02 |
| Proteobacteria | Chromatiales | Allochromatium | Allochromatium vinosum | 0.05 |
| Proteobacteria | Chromatiales | Nitrosococcus | Nitrosococcus halophilus | 0.04 |
| Proteobacteria | Chromatiales | Nitrosococcus | Nitrosococcus oceani | 0.06 |
| Proteobacteria | Chromatiales | Nitrosococcus | Nitrosococcus watsoni | 0.01 |
| Proteobacteria | Chromatiales | Alkalilimnicola | Alkalilimnicola ehrlichii | 0.05 |
| Proteobacteria | Chromatiales | Halorhodospira | Halorhodospira halophila | 0.05 |
| Proteobacteria | Chromatiales | Nitrococcus | Nitrococcus mobilis | 0.04 |
| Proteobacteria | Chromatiales | Thioalkalivibrio | Thioalkalivibrio sp. HL-EbGR7 | 0.06 |
| Proteobacteria | Chromatiales | Thioalkalivibrio | Thioalkalivibrio sp. K90mix | 0.02 |
| Proteobacteria | Chromatiales | Halothiobacillus | Halothiobacillus neapolitanus | 0.02 |
| Proteobacteria | Enterobacteriales | Arsenophonus | Arsenophonus nasoniae | 0.01 |
| Proteobacteria | Enterobacteriales | Buchnera | Buchnera aphidicola | 0.01 |
| Proteobacteria | Enterobacteriales | Citrobacter | Citrobacter freundii | 0.01 |
| Proteobacteria | Enterobacteriales | Citrobacter | Citrobacter koseri | 0.11 |
| Proteobacteria | Enterobacteriales | Citrobacter | Citrobacter rodentium | 0.05 |
| Proteobacteria | Enterobacteriales | Citrobacter | Citrobacter sp. 30_2 | 0.03 |
| Proteobacteria | Enterobacteriales | Citrobacter | Citrobacter youngae | 0.05 |
| Proteobacteria | Enterobacteriales | Cronobacter | Cronobacter sakazakii | 0.04 |
| Proteobacteria | Enterobacteriales | Cronobacter | Cronobacter turicensis | 0.03 |
| Proteobacteria | Enterobacteriales | Dickeya | Dickeya dadantii | 0.11 |
| Proteobacteria | Enterobacteriales | Dickeya | Dickeya zeae | 0.03 |
| Proteobacteria | Enterobacteriales | Edwardsiella | Edwardsiella ictaluri | 0.02 |
| Proteobacteria | Enterobacteriales | Edwardsiella | Edwardsiella tarda | 0.06 |
| Proteobacteria | Enterobacteriales | Enterobacter | Enterobacter cancerogenus | 0.04 |
| Proteobacteria | Enterobacteriales | Enterobacter | Enterobacter cloacae | 0.11 |
| Proteobacteria | Enterobacteriales | Enterobacter | Enterobacter sp. | 0.06 |
| Proteobacteria | Enterobacteriales | Enterobacter | Enterobacter sp. 638 | 0.12 |
| Proteobacteria | Enterobacteriales | Erwinia | Erwinia amylovora | 0.02 |
| Proteobacteria | Enterobacteriales | Erwinia | Erwinia billingiae | 0.03 |
| Proteobacteria | Enterobacteriales | Erwinia | Erwinia pyrifoliae | 0.02 |
| Proteobacteria | Enterobacteriales | Erwinia | Erwinia sp. Ejp617 | 0.01 |
| Proteobacteria | Enterobacteriales | Erwinia | Erwinia tasmaniensis | 0.03 |
| Proteobacteria | Enterobacteriales | Escherichia | Escherichia albertii | 0.06 |
| Proteobacteria | Enterobacteriales | Escherichia | Escherichia coli | 2.78 |
| Proteobacteria | Enterobacteriales | Escherichia | Escherichia fergusonii | 0.09 |
| Proteobacteria | Enterobacteriales | Escherichia | Escherichia sp. 1_1_43 | 0.12 |
| Proteobacteria | Enterobacteriales | Escherichia | Escherichia sp. 3_2_53FAA | 0.16 |
| Proteobacteria | Enterobacteriales | Escherichia | Escherichia sp. 4_1_40B | 0.40 |
| Proteobacteria | Enterobacteriales | Klebsiella | Klebsiella pneumoniae | 0.25 |
| Proteobacteria | Enterobacteriales | Klebsiella | Klebsiella sp. | 0.04 |
| Proteobacteria | Enterobacteriales | Klebsiella | Klebsiella sp. 1_1_55 | 0.04 |
| Proteobacteria | Enterobacteriales | Klebsiella | Klebsiella variicola | 0.05 |
| Proteobacteria | Enterobacteriales | Pantoea | Pantoea ananatis | 0.04 |
| Proteobacteria | Enterobacteriales | Pantoea | Pantoea sp. | 0.04 |
| Proteobacteria | Enterobacteriales | Pantoea | Pantoea sp. At-9b | 0.04 |
| Proteobacteria | Enterobacteriales | Pantoea | Pantoea sp. aB | 0.02 |
| Proteobacteria | Enterobacteriales | Pantoea | Pantoea vagans | 0.03 |
| Proteobacteria | Enterobacteriales | Pectobacterium | Pectobacterium atrosepticum | 0.09 |
| Proteobacteria | Enterobacteriales | Pectobacterium | Pectobacterium carotovorum | 0.05 |
| Proteobacteria | Enterobacteriales | Pectobacterium | Pectobacterium wasabiae | 0.03 |
| Proteobacteria | Enterobacteriales | Photorhabdus | Photorhabdus asymbiotica | 0.03 |
| Proteobacteria | Enterobacteriales | Photorhabdus | Photorhabdus luminescens | 0.04 |
| Proteobacteria | Enterobacteriales | Proteus | Proteus mirabilis | 0.03 |
| Proteobacteria | Enterobacteriales | Proteus | Proteus penneri | 0.02 |
| Proteobacteria | Enterobacteriales | Providencia | Providencia alcalifaciens | 0.02 |
| Proteobacteria | Enterobacteriales | Providencia | Providencia rettgeri | 0.01 |
| Proteobacteria | Enterobacteriales | Providencia | Providencia rustigianii | 0.01 |
| Proteobacteria | Enterobacteriales | Providencia | Providencia stuartii | 0.02 |
| Proteobacteria | Enterobacteriales | Rahnella | Rahnella sp. Y9602 | 0.03 |
| Proteobacteria | Enterobacteriales | Salmonella | Salmonella bongori | 0.05 |
| Proteobacteria | Enterobacteriales | Salmonella | Salmonella enterica | 0.33 |
| Proteobacteria | Enterobacteriales | Serratia | Serratia marcescens | 0.07 |
| Proteobacteria | Enterobacteriales | Serratia | Serratia odorifera | 0.09 |
| Proteobacteria | Enterobacteriales | Serratia | Serratia proteamaculans | 0.13 |
| Proteobacteria | Enterobacteriales | Shigella | Shigella boydii | 0.31 |
| Proteobacteria | Enterobacteriales | Shigella | Shigella dysenteriae | 0.34 |
| Proteobacteria | Enterobacteriales | Shigella | Shigella flexneri | 0.48 |
| Proteobacteria | Enterobacteriales | Shigella | Shigella sonnei | 0.32 |
| Proteobacteria | Enterobacteriales | Shigella | Shigella sp. | 0.20 |
| Proteobacteria | Enterobacteriales | Shigella | Shigella sp. D9 | 0.23 |
| Proteobacteria | Enterobacteriales | Sodalis | Sodalis glossinidius | 0.02 |
| Proteobacteria | Enterobacteriales | Xenorhabdus | Xenorhabdus bovienii | 0.02 |
| Proteobacteria | Enterobacteriales | Xenorhabdus | Xenorhabdus nematophila | 0.02 |
| Proteobacteria | Enterobacteriales | Yersinia | Yersinia aldovae | 0.02 |
| Proteobacteria | Enterobacteriales | Yersinia | Yersinia bercovieri | 0.02 |
| Proteobacteria | Enterobacteriales | Yersinia | Yersinia enterocolitica | 0.09 |
| Proteobacteria | Enterobacteriales | Yersinia | Yersinia frederiksenii | 0.04 |
| Proteobacteria | Enterobacteriales | Yersinia | Yersinia intermedia | 0.04 |
| Proteobacteria | Enterobacteriales | Yersinia | Yersinia kristensenii | 0.02 |
| Proteobacteria | Enterobacteriales | Yersinia | Yersinia mollaretii | 0.03 |
| Proteobacteria | Enterobacteriales | Yersinia | Yersinia pestis | 0.12 |
| Proteobacteria | Enterobacteriales | Yersinia | Yersinia pseudotuberculosis | 0.11 |
| Proteobacteria | Enterobacteriales | Yersinia | Yersinia rohdei | 0.03 |
| Proteobacteria | Enterobacteriales | Yersinia | Yersinia ruckeri | 0.03 |
| Proteobacteria | Enterobacteriales | unclassified (derived from Enterobacteriaceae) | Enterobacteriaceae bacterium 9_2_54FAA | 0.01 |
| Proteobacteria | Legionellales | Coxiella | Coxiella burnetii | 0.02 |
| Proteobacteria | Legionellales | Legionella | Legionella longbeachae | 0.02 |
| Proteobacteria | Legionellales | Legionella | Legionella pneumophila | 0.05 |
| Proteobacteria | Methylococcales | Methylobacter | Methylobacter tundripaludum | 0.01 |
| Proteobacteria | Methylococcales | Methylococcus | Methylococcus capsulatus | 0.03 |
| Proteobacteria | Oceanospirillales | Alcanivorax | Alcanivorax borkumensis | 0.09 |
| Proteobacteria | Oceanospirillales | Alcanivorax | Alcanivorax sp. DG881 | 0.06 |
| Proteobacteria | Oceanospirillales | Kangiella | Kangiella koreensis | 0.03 |
| Proteobacteria | Oceanospirillales | Hahella | Hahella chejuensis | 0.15 |
| Proteobacteria | Oceanospirillales | Chromohalobacter | Chromohalobacter salexigens | 0.12 |
| Proteobacteria | Oceanospirillales | Halomonas | Halomonas elongata | 0.08 |
| Proteobacteria | Oceanospirillales | Bermanella | Bermanella marisrubri | 0.04 |
| Proteobacteria | Oceanospirillales | Marinomonas | Marinomonas sp. MED121 | 0.03 |
| Proteobacteria | Oceanospirillales | Marinomonas | Marinomonas sp. MWYL1 | 0.10 |
| Proteobacteria | Oceanospirillales | Neptuniibacter | Neptuniibacter caesariensis | 0.06 |
| Proteobacteria | Pasteurellales | Actinobacillus | Actinobacillus minor | 0.01 |
| Proteobacteria | Pasteurellales | Actinobacillus | Actinobacillus pleuropneumoniae | 0.04 |
| Proteobacteria | Pasteurellales | Actinobacillus | Actinobacillus succinogenes | 0.03 |
| Proteobacteria | Pasteurellales | Aggregatibacter | Aggregatibacter actinomycetemcomitans | 0.02 |
| Proteobacteria | Pasteurellales | Aggregatibacter | Aggregatibacter aphrophilus | 0.01 |
| Proteobacteria | Pasteurellales | Aggregatibacter | Aggregatibacter segnis | 0.01 |
| Proteobacteria | Pasteurellales | Basfia | Mannheimia succiniciproducens | 0.04 |
| Proteobacteria | Pasteurellales | Haemophilus | Haemophilus ducreyi | 0.01 |
| Proteobacteria | Pasteurellales | Haemophilus | Haemophilus haemoglobinophilus | 0.03 |
| Proteobacteria | Pasteurellales | Haemophilus | Haemophilus influenzae | 0.08 |
| Proteobacteria | Pasteurellales | Haemophilus | Haemophilus parainfluenzae | 0.01 |
| Proteobacteria | Pasteurellales | Haemophilus | Haemophilus parasuis | 0.02 |
| Proteobacteria | Pasteurellales | Histophilus | Histophilus somni | 0.02 |
| Proteobacteria | Pasteurellales | Mannheimia | Mannheimia haemolytica | 0.03 |
| Proteobacteria | Pasteurellales | Pasteurella | Pasteurella dagmatis | 0.01 |
| Proteobacteria | Pasteurellales | Pasteurella | Pasteurella multocida | 0.03 |
| Proteobacteria | Pseudomonadales | Acinetobacter | Acinetobacter baumannii | 1.84 |
| Proteobacteria | Pseudomonadales | Acinetobacter | Acinetobacter calcoaceticus | 0.17 |
| Proteobacteria | Pseudomonadales | Acinetobacter | Acinetobacter genomosp. 13TU | 0.11 |
| Proteobacteria | Pseudomonadales | Acinetobacter | Acinetobacter genomosp. 3 | 0.10 |
| Proteobacteria | Pseudomonadales | Acinetobacter | Acinetobacter haemolyticus | 0.18 |
| Proteobacteria | Pseudomonadales | Acinetobacter | Acinetobacter johnsonii | 0.30 |
| Proteobacteria | Pseudomonadales | Acinetobacter | Acinetobacter junii | 0.24 |
| Proteobacteria | Pseudomonadales | Acinetobacter | Acinetobacter lwoffii | 0.45 |
| Proteobacteria | Pseudomonadales | Acinetobacter | Acinetobacter radioresistens | 0.32 |
| Proteobacteria | Pseudomonadales | Acinetobacter | Acinetobacter sp. | 0.72 |
| Proteobacteria | Pseudomonadales | Acinetobacter | Acinetobacter sp. ADP1 | 0.68 |
| Proteobacteria | Pseudomonadales | Acinetobacter | Acinetobacter sp. ATCC 27244 | 0.12 |
| Proteobacteria | Pseudomonadales | Acinetobacter | Acinetobacter sp. DR1 | 0.31 |
| Proteobacteria | Pseudomonadales | Enhydrobacter | Enhydrobacter aerosaccus | 0.07 |
| Proteobacteria | Pseudomonadales | Moraxella | Moraxella catarrhalis | 0.08 |
| Proteobacteria | Pseudomonadales | Psychrobacter | Psychrobacter arcticus | 0.21 |
| Proteobacteria | Pseudomonadales | Psychrobacter | Psychrobacter cryohalolentis | 0.29 |
| Proteobacteria | Pseudomonadales | Psychrobacter | Psychrobacter sp. | 0.15 |
| Proteobacteria | Pseudomonadales | Psychrobacter | Psychrobacter sp. PRwf-1 | 0.25 |
| Proteobacteria | Pseudomonadales | Azotobacter | Azotobacter vinelandii | 0.63 |
| Proteobacteria | Pseudomonadales | Cellvibrio | Cellvibrio japonicus | 0.15 |
| Proteobacteria | Pseudomonadales | Pseudomonas | Pseudomonas aeruginosa | 2.34 |
| Proteobacteria | Pseudomonadales | Pseudomonas | Pseudomonas amygdali | 0.37 |
| Proteobacteria | Pseudomonadales | Pseudomonas | Pseudomonas coronafaciens | 0.26 |
| Proteobacteria | Pseudomonadales | Pseudomonas | Pseudomonas entomophila | 0.85 |
| Proteobacteria | Pseudomonadales | Pseudomonas | Pseudomonas fluorescens | 4.33 |
| Proteobacteria | Pseudomonadales | Pseudomonas | Pseudomonas fragi | 0.01 |
| Proteobacteria | Pseudomonadales | Pseudomonas | Pseudomonas mendocina | 1.01 |
| Proteobacteria | Pseudomonadales | Pseudomonas | Pseudomonas putida | 2.86 |
| Proteobacteria | Pseudomonadales | Pseudomonas | Pseudomonas resinovorans | 0.01 |
| Proteobacteria | Pseudomonadales | Pseudomonas | Pseudomonas savastanoi | 1.16 |
| Proteobacteria | Pseudomonadales | Pseudomonas | Pseudomonas stutzeri | 1.11 |
| Proteobacteria | Pseudomonadales | Pseudomonas | Pseudomonas syringae | 2.71 |
| Proteobacteria | Pseudomonadales | Pseudomonas | Pseudomonas syringae group genomosp. 3 | 1.18 |
| Proteobacteria | Pseudomonadales | Pseudomonas | Pseudomonas viridiflava | 0.01 |
| Proteobacteria | Thiotrichales | Francisella | Francisella philomiragia | 0.01 |
| Proteobacteria | Thiotrichales | Francisella | Francisella tularensis | 0.01 |
| Proteobacteria | Thiotrichales | Methylophaga | Methylophaga thiooxydans | 0.02 |
| Proteobacteria | Thiotrichales | Thiomicrospira | Thiomicrospira crunogena | 0.03 |
| Proteobacteria | Thiotrichales | Beggiatoa | Beggiatoa sp. PS | 0.01 |
| Proteobacteria | Vibrionales | Aliivibrio | Aliivibrio fischeri | 0.04 |
| Proteobacteria | Vibrionales | Aliivibrio | Aliivibrio salmonicida | 0.02 |
| Proteobacteria | Vibrionales | Photobacterium | Photobacterium angustum | 0.02 |
| Proteobacteria | Vibrionales | Photobacterium | Photobacterium damselae | 0.01 |
| Proteobacteria | Vibrionales | Photobacterium | Photobacterium profundum | 0.08 |
| Proteobacteria | Vibrionales | Vibrio | Vibrio alginolyticus | 0.02 |
| Proteobacteria | Vibrionales | Vibrio | Vibrio caribbenthicus | 0.01 |
| Proteobacteria | Vibrionales | Vibrio | Vibrio cholerae | 0.08 |
| Proteobacteria | Vibrionales | Vibrio | Vibrio coralliilyticus | 0.02 |
| Proteobacteria | Vibrionales | Vibrio | Vibrio furnissii | 0.01 |
| Proteobacteria | Vibrionales | Vibrio | Vibrio harveyi | 0.04 |
| Proteobacteria | Vibrionales | Vibrio | Vibrio metschnikovii | 0.01 |
| Proteobacteria | Vibrionales | Vibrio | Vibrio orientalis | 0.01 |
| Proteobacteria | Vibrionales | Vibrio | Vibrio parahaemolyticus | 0.05 |
| Proteobacteria | Vibrionales | Vibrio | Vibrio shilonii | 0.01 |
| Proteobacteria | Vibrionales | Vibrio | Vibrio sp. | 0.03 |
| Proteobacteria | Vibrionales | Vibrio | Vibrio sp. Ex25 | 0.03 |
| Proteobacteria | Vibrionales | Vibrio | Vibrio sp. MED222 | 0.01 |
| Proteobacteria | Vibrionales | Vibrio | Vibrio splendidus | 0.06 |
| Proteobacteria | Vibrionales | Vibrio | Vibrio vulnificus | 0.06 |
| Proteobacteria | Vibrionales | unclassified (derived from Vibrionaceae) | Grimontia hollisae | 0.01 |
| Proteobacteria | Xanthomonadales | Pseudoxanthomonas | Pseudoxanthomonas suwonensis | 0.14 |
| Proteobacteria | Xanthomonadales | Stenotrophomonas | Stenotrophomonas maltophilia | 0.33 |
| Proteobacteria | Xanthomonadales | Stenotrophomonas | Stenotrophomonas sp. | 0.05 |
| Proteobacteria | Xanthomonadales | Stenotrophomonas | Stenotrophomonas sp. SKA14 | 0.08 |
| Proteobacteria | Xanthomonadales | Xanthomonas | Xanthomonas albilineans | 0.11 |
| Proteobacteria | Xanthomonadales | Xanthomonas | Xanthomonas axonopodis | 0.22 |
| Proteobacteria | Xanthomonadales | Xanthomonas | Xanthomonas campestris | 0.38 |
| Proteobacteria | Xanthomonadales | Xanthomonas | Xanthomonas euvesicatoria | 0.15 |
| Proteobacteria | Xanthomonadales | Xanthomonas | Xanthomonas fuscans | 0.11 |
| Proteobacteria | Xanthomonadales | Xanthomonas | Xanthomonas oryzae | 0.31 |
| Proteobacteria | Xanthomonadales | Xylella | Xylella fastidiosa | 0.20 |
| Proteobacteria | unclassified (derived from Gammaproteobacteria) | Congregibacter | Congregibacter litoralis | 0.05 |
| Proteobacteria | unclassified (derived from Gammaproteobacteria) | Reinekea | Reinekea blandensis | 0.06 |
| Proteobacteria | unclassified (derived from Gammaproteobacteria) | unclassified (derived from Gammaproteobacteria) | Candidatus Ruthia magnifica | 0.01 |
| Proteobacteria | unclassified (derived from Gammaproteobacteria) | unclassified (derived from Gammaproteobacteria) | gamma proteobacterium HTCC2207 | 0.02 |
| Proteobacteria | unclassified (derived from Gammaproteobacteria) | unclassified (derived from Gammaproteobacteria) | gamma proteobacterium HTCC5015 | 0.01 |
| Proteobacteria | unclassified (derived from Gammaproteobacteria) | unclassified (derived from Gammaproteobacteria) | gamma proteobacterium HdN1 | 0.04 |
| Proteobacteria | unclassified (derived from Gammaproteobacteria) | unclassified (derived from Gammaproteobacteria) | gamma proteobacterium NOR5-3 | 0.02 |
| Proteobacteria | unclassified (derived from Gammaproteobacteria) | unclassified (derived from Gammaproteobacteria) | gamma proteobacterium NOR51-B | 0.02 |
| Proteobacteria | unclassified (derived from Gammaproteobacteria) | unclassified (derived from Gammaproteobacteria) | marine gamma proteobacterium HTCC2080 | 0.02 |
| Proteobacteria | unclassified (derived from Gammaproteobacteria) | unclassified (derived from Gammaproteobacteria) | marine gamma proteobacterium HTCC2143 | 0.03 |
| Proteobacteria | unclassified (derived from Gammaproteobacteria) | unclassified (derived from Gammaproteobacteria) | marine gamma proteobacterium HTCC2148 | 0.03 |
| Proteobacteria | Mariprofundales | Mariprofundus | Mariprofundus ferrooxydans | 0.01 |
| Proteobacteria | unclassified (derived from Proteobacteria) | Magnetococcus | Magnetococcus sp. MC-1 | 0.01 |
| Proteobacteria | unclassified (derived from Proteobacteria) | unclassified (derived from Proteobacteria) | Pseudomonas sp. | 0.01 |
| Spirochaetes | Spirochaetales | Brachyspira | Brachyspira hyodysenteriae | 0.03 |
| Spirochaetes | Spirochaetales | Brachyspira | Brachyspira murdochii | 0.03 |
| Spirochaetes | Spirochaetales | Brachyspira | Brachyspira pilosicoli | 0.02 |
| Spirochaetes | Spirochaetales | Leptospira | Leptospira borgpetersenii | 0.01 |
| Spirochaetes | Spirochaetales | Leptospira | Leptospira interrogans | 0.03 |
| Spirochaetes | Spirochaetales | Borrelia | Borrelia afzelii | 0.02 |
| Spirochaetes | Spirochaetales | Borrelia | Borrelia bavariensis | 0.01 |
| Spirochaetes | Spirochaetales | Borrelia | Borrelia burgdorferi | 0.04 |
| Spirochaetes | Spirochaetales | Borrelia | Borrelia garinii | 0.01 |
| Spirochaetes | Spirochaetales | Borrelia | Borrelia hermsii | 0.02 |
| Spirochaetes | Spirochaetales | Borrelia | Borrelia turicatae | 0.02 |
| Spirochaetes | Spirochaetales | Spirochaeta | Spirochaeta smaragdinae | 0.15 |
| Spirochaetes | Spirochaetales | Spirochaeta | Spirochaeta thermophila | 0.06 |
| Spirochaetes | Spirochaetales | Treponema | Treponema denticola | 0.12 |
| Spirochaetes | Spirochaetales | Treponema | Treponema pallidum | 0.07 |
| Spirochaetes | Spirochaetales | Treponema | Treponema phagedenis | 0.03 |
| Spirochaetes | Spirochaetales | Treponema | Treponema vincentii | 0.05 |
| Synergistetes | Synergistales | Aminobacterium | Aminobacterium colombiense | 0.03 |
| Synergistetes | Synergistales | Anaerobaculum | Anaerobaculum hydrogeniformans | 0.01 |
| Synergistetes | Synergistales | Dethiosulfovibrio | Dethiosulfovibrio peptidovorans | 0.05 |
| Synergistetes | Synergistales | Pyramidobacter | Pyramidobacter piscolens | 0.03 |
| Synergistetes | Synergistales | Thermanaerovibrio | Thermanaerovibrio acidaminovorans | 0.01 |
| Synergistetes | unclassified (derived from Synergistetes) | unclassified (derived from Synergistetes) | Synergistetes bacterium SGP1 | 0.01 |
| Tenericutes | Acholeplasmatales | Acholeplasma | Acholeplasma laidlawii | 0.02 |
| Tenericutes | Acholeplasmatales | Candidatus Phytoplasma | Onion yellows phytoplasma | 0.01 |
| Tenericutes | Mycoplasmatales | Mycoplasma | Mycoplasma capricolum | 0.01 |
| Tenericutes | Mycoplasmatales | Mycoplasma | Mycoplasma fermentans | 0.01 |
| Tenericutes | Mycoplasmatales | Mycoplasma | Mycoplasma mycoides | 0.01 |
| Tenericutes | Mycoplasmatales | Mycoplasma | Mycoplasma pneumoniae | 0.01 |
| Thermotogae | Thermotogales | Fervidobacterium | Fervidobacterium nodosum | 0.02 |
| Thermotogae | Thermotogales | Petrotoga | Petrotoga mobilis | 0.02 |
| Thermotogae | Thermotogales | Thermosipho | Thermosipho africanus | 0.01 |
| Thermotogae | Thermotogales | Thermosipho | Thermosipho melanesiensis | 0.01 |
| Thermotogae | Thermotogales | Thermotoga | Thermotoga lettingae | 0.02 |
| Thermotogae | Thermotogales | Thermotoga | Thermotoga maritima | 0.02 |
| Thermotogae | Thermotogales | Thermotoga | Thermotoga petrophila | 0.02 |
| Thermotogae | Thermotogales | Thermotoga | Thermotoga sp. RQ2 | 0.01 |
| Thermotogae | Thermotogales | unclassified (derived from Thermotogales) | Thermotogales bacterium mesG1.Ag.4.2 | 0.01 |
| Verrucomicrobia | Puniceicoccales | Coraliomargarita | Coraliomargarita akajimensis | 0.03 |
| Verrucomicrobia | unclassified (derived from Opitutae) | Opitutus | Opitutus terrae | 0.10 |
| Verrucomicrobia | unclassified (derived from Opitutae) | unclassified (derived from Opitutaceae) | Opitutaceae bacterium TAV2 | 0.03 |
| Verrucomicrobia | unclassified (derived from Spartobacteria) | Chthoniobacter | Chthoniobacter flavus | 0.05 |
| Verrucomicrobia | Verrucomicrobiales | unclassified (derived from Verrucomicrobia subdivision 3) | bacterium Ellin514 | 0.03 |
| Verrucomicrobia | Verrucomicrobiales | Akkermansia | Akkermansia muciniphila | 1.44 |
| Verrucomicrobia | Verrucomicrobiales | Verrucomicrobium | Verrucomicrobium spinosum | 0.07 |
| Verrucomicrobia | Verrucomicrobiales | unclassified (derived from Verrucomicrobiales) | Verrucomicrobiae bacterium DG1235 | 0.02 |
| Verrucomicrobia | Methylacidiphilales | Methylacidiphilum | Methylacidiphilum infernorum | 0.02 |
