# Supplementary material for: Metagenomic Analysis of the Pygmy Loris Fecal Microbiome Reveals Unique Functional Capacity Related to Metabolism of Aromatic Compounds
Source: PLoS One. 2013 Feb 15;8(2):e56565. doi: 10.1371/journal.pone.0056565 (PMC3574064; doi:10.1371/journal.pone.0056565)
Supplement: Table S3 — Phylogenetic classification of fungi in the pygmy loris metagenome. (DOCX) [file pone.0056565.s006.docx]

**Table S3. Phylogenetic classification of fungi in the pygmy loris metagenome**

| phylum | class | order | genus | species | WFH (%) |
| --- | --- | --- | --- | --- | --- |
| Ascomycota | Dothideomycetes | Pleosporales | Leptosphaeria | Leptosphaeria maculans | 0.01 |
|  |  |  | Phaeosphaeria | Phaeosphaeria nodorum | 0.01 |
|  |  |  | Pyrenophora | Pyrenophora tritici-repentis | 0.01 |
|  | Eurotiomycetes | Eurotiales | Aspergillus | Aspergillus flavus | 0.01 |
|  |  |  |  | Aspergillus niger | 0.01 |
|  |  |  |  | Aspergillus oryzae | 0.01 |
|  |  |  |  | Aspergillus terreus | 0.01 |
|  |  |  | Emericella | Emericella nidulans | 0.01 |
|  |  |  | Neosartorya | Neosartorya fumigata | 0.03 |
|  |  |  | Penicillium | Penicillium chrysogenum | 0.01 |
|  |  |  | Talaromyces | Talaromyces stipitatus | 0.01 |
|  |  | Onygenales | Ajellomyces | Ajellomyces capsulatus | 0.01 |
|  |  |  | Trichophyton | Trichophyton verrucosum | 0.01 |
|  | Leotiomycetes | Helotiales | Botryotinia | Botryotinia fuckeliana | 0.01 |
|  |  |  | Sclerotinia | Sclerotinia sclerotiorum | 0.02 |
|  | Pezizomycetes | Pezizales | Tuber | Tuber melanosporum | 0.01 |
|  | Saccharomycetes | Saccharomycetales | Scheffersomyces | Scheffersomyces stipitis | 0.01 |
|  |  |  | Lachancea | Lachancea thermotolerans | 0.01 |
|  |  |  | Saccharomyces | Saccharomyces cerevisiae | 0.02 |
|  | Sordariomycetes | Hypocreales | Metarhizium | Metarhizium anisopliae | 0.01 |
|  |  |  | Gibberella | Gibberella zeae | 0.03 |
|  |  |  | Nectria | Nectria haematococca | 0.01 |
|  |  | Magnaporthales | Magnaporthe | Magnaporthe oryzae | 0.02 |
|  |  | Ophiostomatales | Grosmannia | Grosmannia clavigera | 0.01 |
|  |  | Phyllachorales | Verticillium | Verticillium albo-atrum | 0.01 |
|  |  | Sordariales | Chaetomium | Chaetomium globosum | 0.01 |
|  |  |  | Podospora | Podospora anserina | 0.01 |
|  |  |  | Neurospora | Neurospora crassa | 0.03 |
|  |  | unclassified (derived from Sordariomycetes) | Glomerella | Glomerella graminicola | 0.01 |
| Basidiomycota | Tremellomycetes | Tremellales | Filobasidiella | Cryptococcus neoformans | 0.01 |
|  | Ustilaginomycetes | Ustilaginales | Ustilago | Ustilago maydis | 0.01 |
| Microsporidia | unclassified (derived from Microsporidia) | unclassified (derived from Microsporidia) | Enterocytozoon | Enterocytozoon bieneusi | 0.01 |
