# Supplementary material for: Metagenomic Analysis of the Pygmy Loris Fecal Microbiome Reveals Unique Functional Capacity Related to Metabolism of Aromatic Compounds
Source: PLoS One. 2013 Feb 15;8(2):e56565. doi: 10.1371/journal.pone.0056565 (PMC3574064; doi:10.1371/journal.pone.0056565)
Supplement: Table S4 — Phylogenetic classification of archaea in the pygmy loris metagenome. (DOCX) [file pone.0056565.s007.docx]

**Table S4. Phylogenetic classification of archaea in the pygmy loris metagenome**

| phylum | class | order | strain | WFH (%) |
| --- | --- | --- | --- | --- |
| Crenarchaeota | Thermoprotei | Desulfurococcales | Staphylothermus marinus F1 | 0.01 |
| Euryarchaeota | Archaeoglobi | Archaeoglobales | Archaeoglobus fulgidus DSM 4304 | 0.01 |
|  |  |  | Archaeoglobus fulgidus DSM 4304 (VC-16) | 0.01 |
|  | Halobacteria | Halobacteriales | Halobacterium sp. NRC-1 | 0.01 |
|  |  |  | Halogeometricum borinquense DSM 11551 | 0.01 |
|  |  |  | Haloquadratum walsbyi DSM 16790 | 0.01 |
|  |  |  | Halorhabdus utahensis DSM 12940 | 0.01 |
|  |  |  | Haloterrigena turkmenica DSM 5511 | 0.01 |
|  | Methanobacteria | Methanobacteriales | Methanobrevibacter ruminantium M1 | 0.01 |
|  |  |  | Methanobrevibacter smithii ATCC 35061 | 0.02 |
|  |  |  | Methanosphaera stadtmanae DSM 3091 | 0.01 |
|  |  |  | Methanobacterium thermoautotrophicum | 0.01 |
|  | Methanococci | Methanococcales | Methanocaldococcus jannaschii (Methanococcus jannaschii) | 0.01 |
|  |  |  | Methanocaldococcus jannaschii DSM 2661 | 0.01 |
|  |  |  | Methanocaldococcus jannaschii DSM 2661 | 0.01 |
|  |  |  | Methanococcus maripaludis C7 | 0.01 |
|  |  |  | Methanococcus maripaludis S2 | 0.01 |
|  |  |  | Methanococcus vannielii SB | 0.01 |
|  | Methanomicrobia | Methanomicrobiales | Methanocorpusculum labreanum Z | 0.03 |
|  |  |  | Methanoculleus marisnigri JR1 | 0.01 |
|  |  |  | Methanospirillum hungatei (strain JF-1 / DSM 864) | 0.01 |
|  |  |  | Methanospirillum hungatei JF-1 | 0.01 |
|  |  |  | Candidatus Methanoregula boonei 6A8 | 0.01 |
|  |  |  | Methanosphaerula palustris E1-9c | 0.01 |
|  |  | Methanosarcinales | Methanosaeta thermophila PT | 0.01 |
|  |  |  | Methanococcoides burtonii DSM 6242 | 0.01 |
|  |  |  | Methanosarcina acetivorans C2A | 0.02 |
|  |  |  | Methanosarcina barkeri (strain Fusaro / DSM 804) | 0.01 |
|  |  |  | Methanosarcina barkeri fusaro | 0.01 |
|  |  |  | Methanosarcina barkeri fusaro, chromosome 1 | 0.01 |
|  |  |  | Methanosarcina barkeri str. Fusaro | 0.02 |
|  |  |  | Methanosarcina barkeri str. fusaro | 0.01 |
|  |  |  | Methanosarcina mazei Go1 | 0.01 |
|  | Thermococci | Thermococcales | Pyrococcus abyssi GE5 | 0.01 |
|  |  |  | Pyrococcus furiosus DSM 3638 | 0.01 |
