# Supplementary material for: Metagenomic Analysis of the Pygmy Loris Fecal Microbiome Reveals Unique Functional Capacity Related to Metabolism of Aromatic Compounds
Source: PLoS One. 2013 Feb 15;8(2):e56565. doi: 10.1371/journal.pone.0056565 (PMC3574064; doi:10.1371/journal.pone.0056565)
Supplement: Table S5 — Phylogenetic classification of viruses in the pygmy loris metagenome. (DOCX) [file pone.0056565.s008.docx]

**Table S5. Phylogenetic classification of viruses in the pygmy loris metagenome**

| group | order | family | genus | strain | WFH (%) |
| --- | --- | --- | --- | --- | --- |
| dsDNA viruses, no RNA stage | Caudovirales | Myoviridae | P1-like viruses | Enterobacteria phage P1 | 0.01 |
|  |  | Siphoviridae | Lambda-like viruses | Bacteriophage lambda | 0.01 |
|  |  |  |  | Enterobacteria phage DE3 | 0.01 |
|  |  |  |  | Enterobacteria phage lambda | 0.01 |
