# Supplementary material for: Metagenomic Analysis of the Pygmy Loris Fecal Microbiome Reveals Unique Functional Capacity Related to Metabolism of Aromatic Compounds
Source: PLoS One. 2013 Feb 15;8(2):e56565. doi: 10.1371/journal.pone.0056565 (PMC3574064; doi:10.1371/journal.pone.0056565)
Supplement: Table S6 — Presence of carbohydrate active enzyme families in the pygmy loris metagenome. (DOCX) [file pone.0056565.s009.docx]

Table S6. Presence of carbohydrate active enzyme families in the pygmy loris metagenome

| GH family* | Sequence # | % of total GH |  | GT family* | Sequence # | % of total GT |
| --- | --- | --- | --- | --- | --- | --- |
| GH1 | 9 | 0.57% |  | GT1 | 9 | 2.14% |
| GH2 | 134 | 8.45% |  | GT2 | 148 | 35.24% |
| GH3 | 142 | 8.96% |  | GT3 | 9 | 2.14% |
| GH4 | 4 | 0.25% |  | GT4 | 96 | 22.86% |
| GH5 | 21 | 1.32% |  | GT5 | 6 | 1.43% |
| GH6 | 2 | 0.13% |  | GT8 | 4 | 0.95% |
| GH8 | 9 | 0.57% |  | GT9 | 13 | 3.10% |
| GH9 | 28 | 1.77% |  | GT11 | 2 | 0.48% |
| GH10 | 17 | 1.07% |  | GT14 | 1 | 0.24% |
| GH13 | 88 | 5.55% |  | GT19 | 13 | 3.10% |
| GH14 | 1 | 0.06% |  | GT20 | 11 | 2.62% |
| GH15 | 6 | 0.38% |  | GT21 | 1 | 0.24% |
| GH16 | 25 | 1.58% |  | GT23 | 1 | 0.24% |
| GH17 | 2 | 0.13% |  | GT25 | 1 | 0.24% |
| GH18 | 18 | 1.14% |  | GT28 | 22 | 5.24% |
| GH19 | 4 | 0.25% |  | GT30 | 11 | 2.62% |
| GH20 | 55 | 3.47% |  | GT32 | 3 | 0.71% |
| GH23 | 27 | 1.70% |  | GT35 | 37 | 8.81% |
| GH24 | 4 | 0.25% |  | GT41 | 1 | 0.24% |
| GH25 | 5 | 0.32% |  | GT51 | 16 | 3.81% |
| GH26 | 4 | 0.25% |  | GT53 | 1 | 0.24% |
| GH27 | 13 | 0.82% |  | GT66 | 2 | 0.48% |
| GH28 | 71 | 4.48% |  | GT81 | 2 | 0.48% |
| GH29 | 33 | 2.08% |  | GT83 | 6 | 1.43% |
| GH30 | 29 | 1.83% |  | GT85 | 2 | 0.48% |
| GH31 | 34 | 2.15% |  | GT87 | 1 | 0.24% |
| GH32 | 10 | 0.63% |  | GT89 | 1 | 0.24% |
| GH33 | 16 | 1.01% |  | **Totle GT** | **420** | **100.00%** |
| GH35 | 11 | 0.69% |  |  |  |  |
| GH36 | 20 | 1.26% |  | **CBM family*** | **Sequence #** | **% of total CBM** |
| GH37 | 1 | 0.06% |  | CBM9 | 2 | 3.70% |
| GH38 | 13 | 0.82% |  | CBM13 | 2 | 3.70% |
| GH39 | 3 | 0.19% |  | CBM26 | 2 | 3.70% |
| GH42 | 11 | 0.69% |  | CBM32 | 22 | 40.74% |
| GH43 | 103 | 6.50% |  | CBM33 | 2 | 3.70% |
| GH48 | 1 | 0.06% |  | CBM35 | 4 | 7.41% |
| GH50 | 6 | 0.38% |  | CBM43 | 1 | 1.85% |
| GH51 | 23 | 1.45% |  | CBM48 | 8 | 14.81% |
| GH53 | 6 | 0.38% |  | CBM50 | 7 | 12.96% |
| GH55 | 9 | 0.57% |  | CBM54 | 3 | 5.56% |
| GH57 | 3 | 0.19% |  | CBM57 | 1 | 1.85% |
| GH63 | 10 | 0.63% |  | **Totle CBM** | **54** | **100.00%** |
| GH65 | 12 | 0.76% |  |  |  |  |
| GH66 | 2 | 0.13% |  | **CE family*** | **Sequence #** | **% of total CE** |
| GH67 | 2 | 0.13% |  | CE1 | 25 | 21.19% |
| GH73 | 8 | 0.50% |  | CE2 | 2 | 1.69% |
| GH74 | 3 | 0.19% |  | CE4 | 15 | 12.71% |
| GH76 | 10 | 0.63% |  | CE6 | 2 | 1.69% |
| GH77 | 24 | 1.51% |  | CE7 | 8 | 6.78% |
| GH78 | 29 | 1.83% |  | CE8 | 13 | 11.02% |
| GH81 | 2 | 0.13% |  | CE9 | 19 | 16.10% |
| GH84 | 3 | 0.19% |  | CE11 | 14 | 11.86% |
| GH87 | 1 | 0.06% |  | CE12 | 10 | 8.47% |
| GH88 | 26 | 1.64% |  | CE14 | 5 | 4.24% |
| GH89 | 13 | 0.82% |  | CE15 | 5 | 4.24% |
| GH92 | 94 | 5.93% |  | **Totle CE** | **118** | **100.00%** |
| GH93 | 1 | 0.06% |  |  |  |  |
| GH94 | 6 | 0.38% |  | **PL family*** | **Sequence #** | **% of total PL** |
| GH95 | 28 | 1.77% |  | PL1 | 27 | 32.53% |
| GH97 | 89 | 5.62% |  | PL8 | 11 | 13.25% |
| GH103 | 6 | 0.38% |  | PL9 | 1 | 1.20% |
| GH104 | 1 | 0.06% |  | PL10 | 7 | 8.43% |
| GH105 | 42 | 2.65% |  | PL11 | 18 | 21.69% |
| GH106 | 21 | 1.32% |  | PL12 | 6 | 7.23% |
| GH108 | 1 | 0.06% |  | PL13 | 4 | 4.82% |
| GH109 | 15 | 0.95% |  | PL15 | 8 | 9.64% |
| GH110 | 8 | 0.50% |  | PL22 | 1 | 1.20% |
| GH111 | 2 | 0.13% |  | **Totle PL** | **83** | **100.00%** |
| GH112 | 3 | 0.19% |  |  |  |  |
| GH115 | 27 | 1.70% |  |  |  |  |
| GH116 | 5 | 0.32% |  |  |  |  |
| GH117 | 2 | 0.13% |  |  |  |  |
| GH120 | 1 | 0.06% |  |  |  |  |
| GH121 | 1 | 0.06% |  |  |  |  |
| GH123 | 7 | 0.44% |  |  |  |  |
| GH125 | 10 | 0.63% |  |  |  |  |
| GH127 | 31 | 1.96% |  |  |  |  |
| GH128 | 1 | 0.06% |  |  |  |  |
| GH130 | 17 | 1.07% |  |  |  |  |
| Totle GH | **1585** | **100.00%** |  |  |  |  |

* Based on CAZy database (www.cazy.org)
