# Supplementary material for: Metagenomic Analysis of the Pygmy Loris Fecal Microbiome Reveals Unique Functional Capacity Related to Metabolism of Aromatic Compounds
Source: PLoS One. 2013 Feb 15;8(2):e56565. doi: 10.1371/journal.pone.0056565 (PMC3574064; doi:10.1371/journal.pone.0056565)
Supplement: Table S7 — Overview of the MG-RAST metagenomes chosen for comparison. (DOCX) [file pone.0056565.s010.docx]

Table S7. Overview of the MG-RAST metagenomes chosen for comparison

| Metagenome  (MG-RAST accession) | Host | Sequence number | Total Size  MB | Shortest | Longest | Average |
| --- | --- | --- | --- | --- | --- | --- |
| WFH (4476304.3) | Pygmy loris | 61281 | 27.2 | 49 | 1201 | 443 |
| LMC (4440463.3) | Lean mouse | 10845 | 8.4 | 77 | 1307 | 781.8 |
| OMC (4440464.3) | Obese mouse | 11857 | 9.1 | 112 | 1187 | 764.7 |
| F1S (4440939.3) | Human | 28900 | 38 | 92 | 16490 | 1315 |
| HSM (4444130.3) | Malnourished Human | 108486 | 74.2 | 93 | 160132 | 684 |
| CCA (4440283.3) | Chicken | 310801 | 32.3 | 39 | 258 | 103 |
| CCB (4440284.3) | Chicken | 254712 | 26.4 | 40 | 249 | 103 |
| K9C (4444164.3) | Dog (low-fiber diet) | 66969 | 53.2 | 44 | 36188 | 794 |
| K9BP (4444165.3) | Dog (beet pulp diet) | 67761 | 43.6 | 41 | 14401 | 642 |
| CRP (4441682.3) | Cow | 218460 | 22.5 | 35 | 193 | 102 |
